# Development of a virtual source model for Monte Carlo‐based independent dose calculation for varian linac

**DOI:** 10.1002/acm2.13556

**Published:** 2022-02-09

**Authors:** James R. Castle, Jingwei Duan, Xue Feng, Quan Chen

**Affiliations:** ^1^ Carina Medical LLC Lexington Kentucky USA; ^2^ Department of Radiation Medicine University of Kentucky School of Medicine Lexington Kentucky USA

**Keywords:** Monte Carlo independent dose calculation, virtual source model, Varian Linac

## Abstract

Monte Carlo (MC) independent dose calculations are often based on phase‐space files (PSF), as they can accurately represent particle characteristics. PSF generally are large and create a bottleneck in computation time. In addition, the number of independent particles is limited by the PSF, preventing further reduction of statistical uncertainty. The purpose of this study is to develop and validate a virtual source model (VSM) to address these limitations. Particles from existing PSF for the Varian TrueBeam medical linear accelerator 6X, 6XFFF, 10X, and 10XFFF beam configurations were tallied, analyzed, and used to generate a dual‐source photon VSM that includes electron contamination. The particle density distribution, kinetic energy spectrum, particle direction, and the correlations between characteristics were computed. The VSM models for each beam configuration were validated with water phantom measurements as well as clinical test cases against the original PSF. The new VSM requires 67 MB of disk space for each beam configuration, compared to 50 GB for the PSF from which they are based and effectively remove the bottleneck set by the PSF. At 3% MC uncertainty, the VSM approach reduces the calculation time by a factor of 14 on our server. MC doses obtained using the VSM approach were compared against PSF‐generated doses in clinical test cases and measurements in a water phantom using a gamma index analysis. For all tests, the VSMs were in excellent agreement with PSF doses and measurements (>90% passing voxels between doses and measurements). Results of this study indicate the successful derivation and implementation of a VSM model for Varian Linac that significantly saves computation time without sacrificing accuracy for independent dose calculation.

## 1 INTRODUCTION

Monte Carlo (MC) simulation is regarded as the gold standard for estimating the amount of energy deposited into a medium by ionizing radiation.^1^, ^2^ The clinical utility of using MC simulations for independent dose calculations is often hindered by the computational resources and time required to run these simulations.^3^, ^4^ Often, MC simulations begin with phase‐space files (PSF), which are derived from full simulations of primary electron interactions within the treatment head and store the resulting particles at a reference surface a set distance from the treatment head. Given an accurate representation of the geometries of the treatment head, PSF will contain the most accurate description of the resulting beam. MC dose calculations must be run with a large number of simulated particles to achieve clinically acceptable accuracy. This, in turn, requires input PSF that contain a large (∼billions), but finite, number of particles that take up significant amounts of disk space to store (∼GB). A major bottleneck in MC calculations arises from the input/output (I/O) time required for reading large PSF from the disk. Hard disk drives (HDDs) have buffered file read rates up to ∼150 MB/s, while solid state drives (SSDs) have buffered file read rates up to ∼500 MB/s. Even with the increased read rate for an SSD, a best‐case scenario for reading a 50 GB PSF will take ∼100 s just to load the file. This I/O burden becomes more significant with methods that use multicore processing, as the multiple processes will compete over the same I/O bandwidth.

A common solution to ease the I/O burden is to develop so‐called virtual source models (VSMs),^5^, ^6^, ^7^, ^8^, ^9^, ^10^, ^11^, ^12^, ^13^, ^14^, ^15^, ^16^, ^17^, ^18^, ^19^, ^20^, ^21^ which approximate the behavior of particles at the phase‐space surface. VSMs have two distinct advantages: they take up significantly less disk space and allow for infinite sampling. Particle parameters from the PSF must be modeled precisely for the VSM to yield quality simulations. Numerous studies have been performed to generate VSM for a wide variety of radiotherapy treatment options; each was derived using either experimental measurements^7^, ^9^ or previously‐generated PSF files.^5^, ^6^, ^8^, ^10^, ^11^, ^12^, ^13^, ^14^, ^15^, ^16^, ^17^, ^18^, ^19^, ^20^, ^21^

In this study, a generalized method to derive VSM from International Atomic Energy Agency (IAEA)‐compliant PSF is introduced. Several VSMs were generated from PSF for the Varian (Varian Medical Systems, Palo Alto, CA) TrueBeam medical linear accelerator (Linac) for varying electron beam energies and treatment beam flattening configurations. Each VSM utilizes a dual‐source approach to model photons and includes electron contamination. The validity of this method is demonstrated in three studies. First, comparisons were made between PSF‐ and VSM‐sampled particles at the phase‐space surface and at the treatment isocenter to validate the VSM sampling method. Next, test patient cases were copied and delivered to a solid water phantom from which 2D coronal dose distributions were measured. MC doses obtained using the VSM method were compared to measured doses to validate the MC algorithm. Last, comparisons between the VSM‐ and PSF‐generated MC doses in clinical test cases were made to further validate the VSM method as well as to illustrate the computation time savings afforded by the VSM.

## 2 METHODS

### 2.1 Varian PSF

We obtained IAEA‐compliant PSF from Varian for 6 MV (6X) and 10 MV (10X) electron beam energies. For 6X and 10X beam energies, separate PSF were obtained for the flattening‐filter‐free (FFF) operation mode (6XFFF and 10XFFF, respectively). The PSF contain roughly 2.5 billion particles (photons, electrons, and positrons) per beam configuration. These PSF correspond to “version 2″ from Varian.^22^, ^23^ In short, these files were generated by first simulating electron beams in the accelerator using the code package Parmela^24^ and then transporting through the bending magnet. The electron beam exiting the bend magnet parametrized energy spectrum, spot size, and beam divergence using Gaussian distributions, which were tuned to reference gold beam data.^22^, ^23^ The resulting electrons were then passed to the code package Geant4^25^ to simulate transport through the treatment head.

The results of the Geant4 simulation were tallied at a distance of 73.3 cm from the isocenter (26.7 cm from the focal spot) and saved in a format recommended by the IAEA.^26^ The files contain six parameters for each particle: particle type (photon, electron, or positron), kinetic energy, crossplane, and inplane positions (X and Y, respectively), and crossplane and inplane direction cosines (U and V, respectively). The third direction cosine *W* (*Z*) was set to 1 for all particles.

### 2.2 Dual‐source treatment of photons

We assume that photons in the PSF arise from two sources. We consider “primary photons” as those that originated from the Bremsstrahlung radiation in the treatment head. We consider “secondary” or “scattered photons” as photons that have scattered from interactions in the collimator or in the flattening filter, if present. The PSF do not contain information on the origin of the photons. To come up with a criterion to discriminate between primary and scattered photons, we analyzed the distributions of the position of photons at the focal spot by reverse transporting photons to the focal spot plane (Z=0). Given that the electron beam spot size entering the tungsten target is parameterized by a Gaussian, we performed Gaussian fits to the X and Y profiles around X=Y=0. Using the means and standard deviations from these fits, we defined a primary photon as a photon that has a position at the focal spot plane within the window $X\;=\mu_{x}\;\pm 3\sigma_{x}$ and $Y\;=\mu_{y}\;\pm 3\sigma_{y}$. All photons outside this window were considered scattered photons. The fraction of primary to scattered photons was saved for downstream sampling in MC simulations. The means and standard deviations of these fits are adjustable parameters in our final model in case additional fine tuning is needed.

### 2.3 VSM

Particles were scored at the phase‐space surface (Z=26.7 cm) and split into groups based on particle type. Photons were split into two groups based on primary/scatter classification, while electrons and positrons were grouped together and considered purely scatter to maximize count statistics, as >99% of PSF particles are photons. For each of the three groups, histograms were used to generate probability density functions for the following parameters: particle X position, particle Y position based on X position, particle kinetic energy based on radial position (R), particle U based on X position, and particle V based on Y position. The following subsections outline exactly how each parameter is modeled.

#### 2.3.1 Particle crossplane and inplane positions

The X positions for each particle were counted separately for primary photons, scattered photons, and electrons/positrons. Histograms for photons used a bin width of 0.1 cm, while the electron/positron histogram had a bin width of 0.2 cm to reduce noise. The Y positions of each particle were counted in histograms using the exact grouping and binning as particle X positions. Further, Y position histograms were generated for every X bin to preserve the correlation between particle X and Y position.

#### 2.3.2 Particle kinetic energy

The kinetic energies of each particle were counted separately for primary photons, scattered photons, and electrons/positrons. Histograms for photons used a bin width of 0.02 MeV, while the electron/positron histogram had a bin width of 0.05 MeV to reduce noise. Further, separate kinetic energy histograms were counted based on radial position. A kinetic energy histogram was generated every 0.5 cm in R up to R=5.5 cm. Particles with R>5.5 cm were counted in a single histogram.

#### 2.3.3 Particle crossplane and inplane directions

The U and V direction cosines for each particle were counted separately for primary photons, scattered photons, and electrons/positrons. Instead of counting U and V values directly, we counted the quantities $U^{\prime }-U$ and $V^{\prime }-V$, where $U^{\prime }\equiv X/Z$ and $V^{\prime }\equiv Y/Z$. Our rationale was that most particles direction will, on average, follow their geometric position with respect to the origin at the treatment head with some fluctuations around that vector. This is indeed relevant for primary photons, where such histograms show a peak around 0 with very small Gaussian spread. The relationship is less relevant for scattered photons and electrons/positrons, as the scattering processes do not preserve information on particle origin. Histograms for direction cosine difference used a bin width of 0.0005 to capture the small fluctuation behavior, while the electron/positron histograms used a bin width of 0.02 to reduce noise. Further, $U^{\prime }-U$ histograms were generated for every X bin, and $V^{\prime }-V$ histograms were generated for every Y bin, as fluctuation size was dependent on distance from the origin.

### 2.4 MC simulation for independent dose verification

**FIGURE 1:**
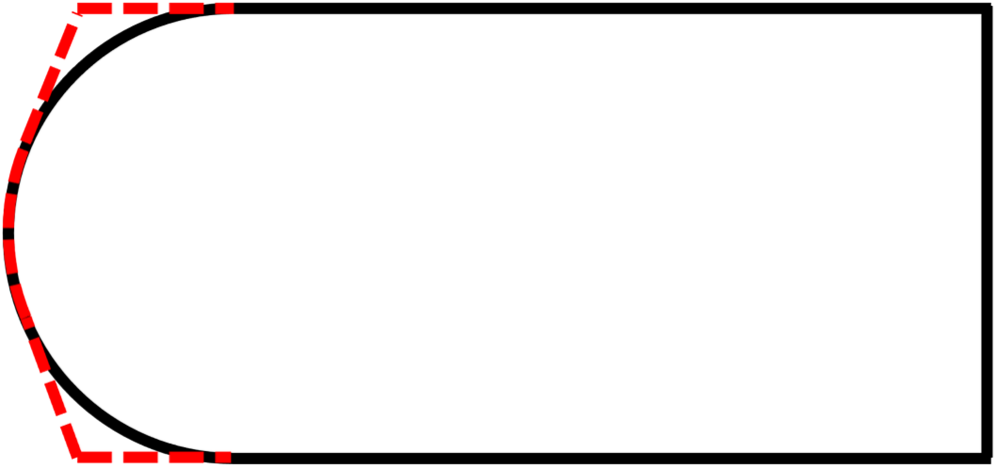
Schematic highlighting the differences in how multi‐leaf collimator leaf tips are modeled in the Monte Carlo framework (solid black line) versus the leaf‐tip design used by the Varian Millennium 120 multi‐leaf collimator (dashed red line). Leaf tips are not drawn to scale

This study is an extension of a previously‐established, cloud‐based tool for MC independent dose calculation.^27^ In short, this tool uses a modified version of the PENELOPE MC software^28^ to include a message‐passing‐interface for parallel computing.^29^ Transport in the jaws and multi‐leaf collimators (MLC) were modeled using first‐order approximations following the Siebers–Keall method.^30^ Specifically, only attenuation and first Compton interactions were considered for primary photons, and only attenuation was considered for scattered photons. Clinical cases used in this study were delivered using a machine with the Varian Millennium 120 MLC, the leaf tips of which are rounded with an 11.3° tip angle; however, within the MC framework, MLC leaf tips are modeled as simply rounded without the tip angle, as illustrated in Figure 1.

**FIGURE 2:**
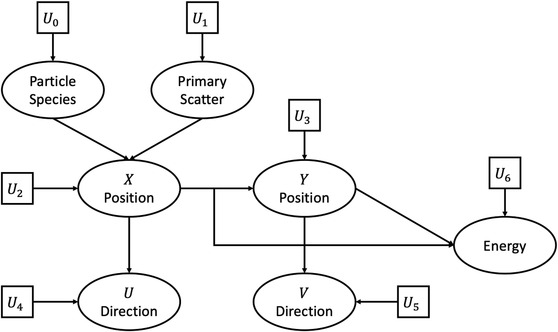
Flow chart illustrating the sampling process utilized in the virtual source model. Seven random uniform numbers ($U_{0}\text{…}U_{6}$) are used to sample particle species, primary/scatter classification, inplane and crossplane position, kinetic energy, and inplane and crossplane direction

To reduce the I/O stress from reading in the large PSF, the histograms for particle X and Y position, kinetic energy, and U and V direction cosines described above were converted into cumulative distribution functions (CDF). As a result, the reading of particle parameters from the PSF was replaced by inverse transform sampling from these CDF. The procedure for sampling particles is illustrated in a flow chart in Figure 2 and is briefly explained here. First, seven random uniform numbers are generated $\left(U_{0}\text{…}U_{6}\right)$. Random numbers $U_{0}$ and $U_{1}$ are used to determine particle species and (if a photon is selected) primary/scatter classification, respectively. This information is used to determine from which inverse CDF to sample for the remaining particles. Random number $U_{2}$ is used to sample particle *X* position, which is then used to determine from which Y position and $U^{\prime }-U$ CDF to sample. Random number $U_{3}$ is used to sample particle Y position, which is then used to determine from which $V^{\prime }-V$ CDF to sample. Random numbers $U_{4}$ and $U_{5}$, in conjunction with particle X and Y position, are used to sample the U and V direction cosines, respectively. Lastly, random number $U_{6}$, in conjunction with particle X and Y position, is used to sample particle kinetic energy. The set of particle species, position, kinetic energy, and direction is used in downstream analysis to transport through the jaws, MLC, and the patient volume.

Previously, the MC independent dose calculation tool read in the PSF directly and implemented a rotational augmentation to re‐use the particle to save I/O overhead. Each particle read was rotated by a random angle four times, which reduced uncertainty in the phase‐space surface by a factor of two at all points except near the central axis. With this new VSM, the rotational augmentation is no longer necessary and results in a uniform statistical uncertainty.

### 2.5 VSM validation data

Radiotherapy cases were collected from the clinical practice at the University of Kentucky Department of Radiation Medicine. The cases used for testing comprise patients treated at University of Kentucky between 2015 and 2021 using a Varian TrueBeam Linac. Radiotherapy plan data sets were generated using the Eclipse V15.6 (Varian Medical System, Palo Alto, CA 94304) TPS. The imaging data sets for each patient were acquired using computed tomography (CT) systems (GE Healthcare, Waukesha, Wisconsin) with 120 kV tube voltage and 2–3 mm slice reconstruction under an in‐house imaging protocol. All data were anonymized to remove the protected health information of human subjects.

### 2.6 VSM validation

The VSM approach was validated in three studies. First, particles were simulated using the VSM outside of the MC dose framework and transported to distances 0, 30, 50, 70, and 100 cm from the phase‐space surface. Global distributions of particle X and Y were generated for each transport distance. In addition, since particle U, V, and kinetic energy do not vary with transport distance, a single global distribution was generated for each. Similar distributions were generated for particles read from the PSF. The two distributions were compared using a $\chi^{2}\;\mathrm{test}$ for two histograms^31^ at the phase‐space surface (*Z* = 26.7 cm) and at the isocenter (≈70 cm transported from the phase‐space surface). A *p*‐value less than 0.05 indicates significant differences between the VSM and PSF histograms.

For the second study, five patient plans were selected for each beam configuration and were copied to a solid water phantom using a TPS for a total of 20 cases. The OCTAVIUS II (PTW, Frieburg, Germany) dose verification system was used. Patient plans were delivered to the water phantom, and measurements of the 2D coronal dose distribution were obtained. The same patient plans and phantom CT images were exported to our MC framework for second check simulation using a 3% statistical uncertainty using the VSM. The CT, TPS dose, and measured dose images are read in DICOM files. Prior to simulation, all dose grids are resampled to have the same grid size and resolution as the CT image. To conserve memory, all images are then downsized to a 256 × 256 axial grid. The MC dose is then calculated using the CT volume. This ensures that all comparisons between doses are using the same resolution. Two‐dimensional slices corresponding to the coronal measurement plane from the MC calculation were exported to the MapCheck software (SunNuclear), which performed a 2D gamma analysis using 3%/3 mm criteria. In both studies, a gamma index passing rate above 90% for voxels receiving greater than 10% of the prescription dose was considered passing in the comparison between the MC and measured doses, which follows the clinical quality assurance procedures at the University of Kentucky.

For the last study, five patient plans, from different patients from previous study, were selected for each beam configuration for a total of 20 test cases. Plans were exported to our MC framework for second check simulation using a 3% statistical uncertainty using both the PSF and VSM models. The CT, TPS dose, and measured dose images are read in DICOM files. Prior to simulation, all dose grids are resampled to have the same grid size and resolution as the CT image. To conserve memory, all images are then downsized to a 256 × 256 axial grid. The MC dose is then calculated using the CT volume. This ensures that all comparisons between doses are using the same resolution. VSM doses were compared against PSF doses through a 3D gamma analysis using 3%/3 mm criteria, which are common criteria used in clinical practice.^32^, ^33^ Three dimensional gamma index passing rates were calculated using the “gamma” function from the python module PyMedPhys.^34^ The computation time between the two MC methods was also assessed. Tests were run on a server with 64 gigabytes (GB) of random access memory (RAM) and a 2.3 GHz central processing unit (CPU). Files were read from an HDD with a buffered disk read rate of ≈250 megabytes per second (MB/s) and a cached memory read rate of ≈10,000 MB/s. The speed of PSF calculations is highly dependent on whether files were read from the disk or were cached from a previous simulation using the same PSF. Calculations for each case were run five times to determine the mean computation time for both cached and non‐cached reads and illustrate best‐ and worst‐case scenarios, respectively. The same number of threads (12) was used in parallel computations for both the VSM and PSF calculations. In addition, the rotational augmentation in the old PSF implementation was removed to allow for a direct comparison between the two methods. Completion times for each case using the VSM method were compared against raw and cached PSF completion times over all cases using paired *t*‐tests. Here, a *p*‐value less than 0.05 indicates a significant difference in the mean computation time between approaches (VSM vs. raw PSF calculation time and VSM vs. cached PSF calculation time).

## 3 RESULTS AND DISCUSSION

### 3.1 VSM

Using the information stored within the Varian PSF, VSMs were generated for four beam configurations. Inverse CDF were stored as binary files containing floating‐point precision lookup tables using probability bin widths of 0.00005. For each beam configuration, the collection of inverse CDF lookup tables uses ∼67 MB of disk space, compared to ∼50 GB for respective PSF. This ∼1000 times reduction in disk space usage is well below the standard I/O disk read rates for HDD and SSD and significantly eases latency in MC calculations. Particles generated from these VSM are sampled in a field‐independent region and do not depend on the positions of the jaws and MLC leaves.

The greatest distinction between the VSMs outlined in this paper with other VSM in the literature is its binned approach to modeling PSF behavior. No fits are performed to the histograms that score particle behavior at the phase‐space surface, rather the histograms are used directly to sample particles for each MC simulation. Compared with most VSM that use functional approaches,^5^, ^6^, ^7^, ^8^, ^9^, ^10^, ^11^, ^12^, ^16^, ^18^, ^20^ our VSM provide a simplified approach that is easy to implement and requires little tuning and commissioning; however, this approach is limited in that it is fully dependent on the results of full simulations of the treatment head. If the PSF resulting from the full simulation do not describe the treatment machine well, neither will our VSM, and new simulations will need to be performed or new PSF will need to be obtained from the IAEA or vendor. Further, we model our VSM using two sources for photons, whereas other VSM may use one^19^, ^20^ or three sources.^21^ Our dual‐source approach is a tradeoff between model complexity and accuracy that are observed in single‐source and triple‐source models, respectively.

### 3.2 Primary photon classification

**FIGURE 3:**
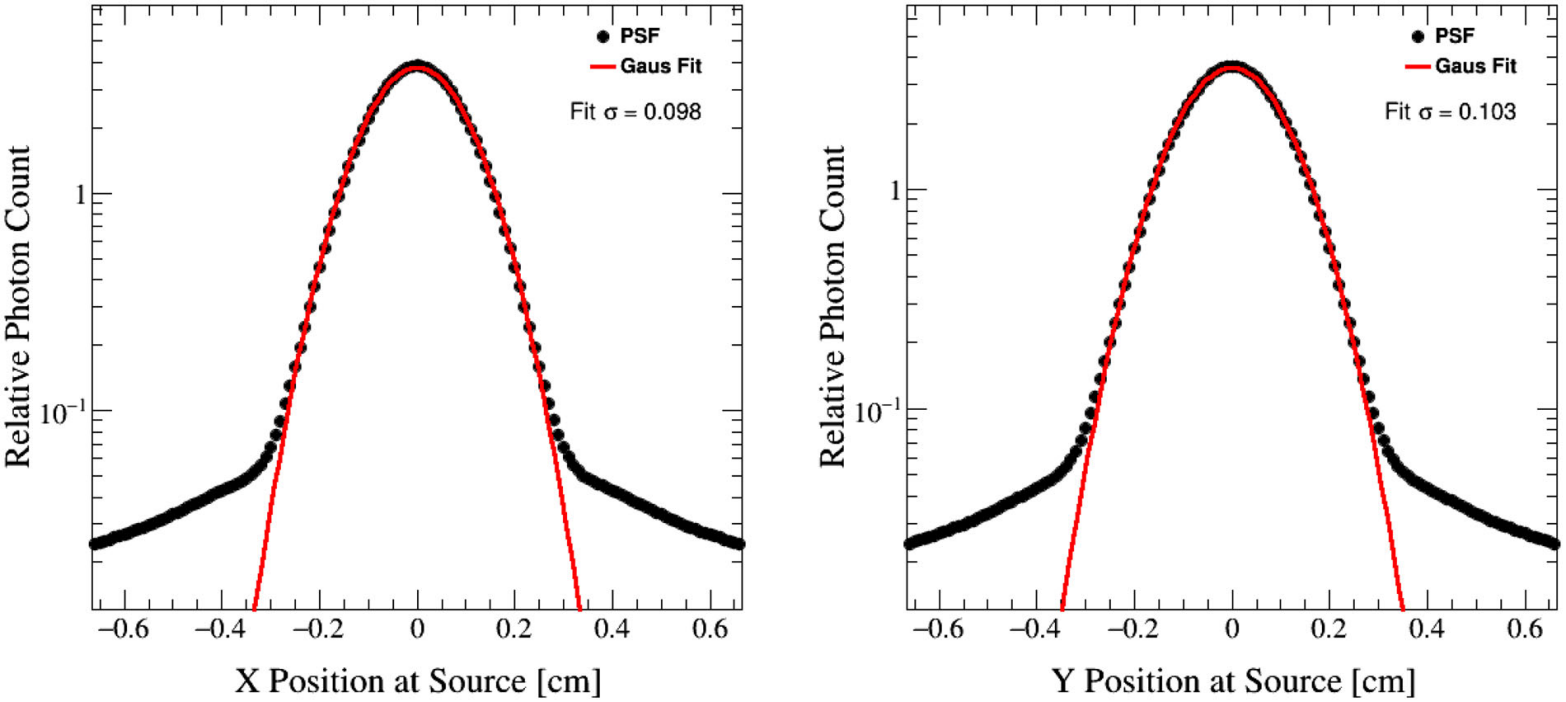
Gaussian fits (red lines) to the positions of photons that were reverse transported from the phase‐space sampling surface to the treatment head (black dots) for the 6X beam configuration

This method utilizes a dual‐source approach for modeling photons as either primary, resulting from the Bremsstrahlung radiation generated in the treatment head, or scattered, resulting from interactions in the collimator or in the flattening filter. The crossplane and inplane positions of photons reverse‐transported to the treatment head are illustrated in Figure 3 for the 6X beam configuration. A prominent Gaussian peak is observed around X=Y=0. This behavior is observed for all beam energies. The PSF do not contain information on the origin of the photons, so we assume this Gaussian peak arises from the shape of the beam focal spot on the target. Therefore, to classify photons as either primary or scattered, Gaussian fits were performed to these central regions. A photon was classified as primary if its position at the treatment head was within a window of three standard deviations of the Gaussian peak ($X\;=\mu_{x}\;\pm 3\sigma_{x}$ and $Y\;=\mu_{y}\;\pm 3\sigma_{y}$). All remaining photons were considered as scattered. Primary and scattered photons parameters were scored separately at the phase‐space surface. Following these criteria, the fraction of primary to scattered photons was also obtained. The results of the Gaussian fit parameters and primary photon fraction are shown in Table 1.

**TABLE 1:** Results of Gaussian fits to the positions of photons that were reverse transported to the treatment head. The fraction of primary photons is determined by counting the number of photons with positions within three standard deviations of the fits

Beam configuration	Fit $\mu_{x}$	Fit $\sigma_{x}$	Fit $\mu_{y}$	Fit $\sigma_{y}$	Primary fraction
6XFFF	−7.2 × 10^−8^	0.092	−1.2 × 10^−6^	0.096	91%
6X	1.8 × 10^−6^	0.098	−1.2 × 10^−6^	0.103	84%
10XFFF	1.9 × 10^−6^	0.106	5.2 × 10^−6^	0.105	90%
10X	−3.6 × 10^−7^	0.112	−3.0 × 10^−7^	0.115	79%
15X	−4.8 × 10^−7^	0.102	−3.5 × 10^−7^	0.098	79%

### 3.3 VSM sampling method validation

**FIGURE 4:**
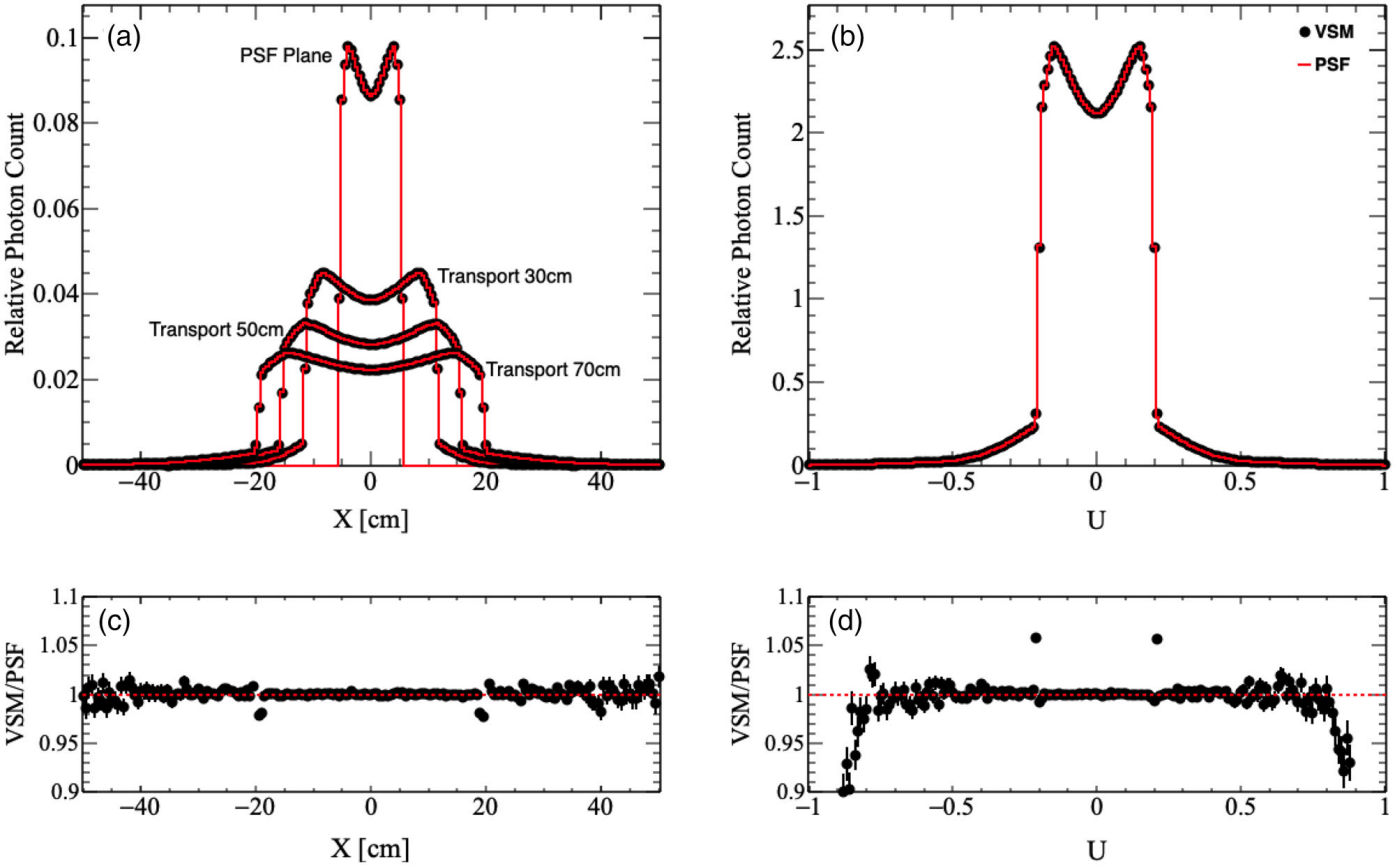
Comparison of (a) particle position and (b) direction for particles sampled using the virtual source model (VSM, black dots) to those read from phase‐space files (PSF, red lines) for the 10X beam configuration. Particles were sampled at the phase‐space surface and were then transported 30, 50, and 70 cm. Ratios of the VSM to the PSF distributions for (c) particle position at the isocenter (70 cm) and (d) particle direction are shown below respective distributions

**FIGURE 5:**
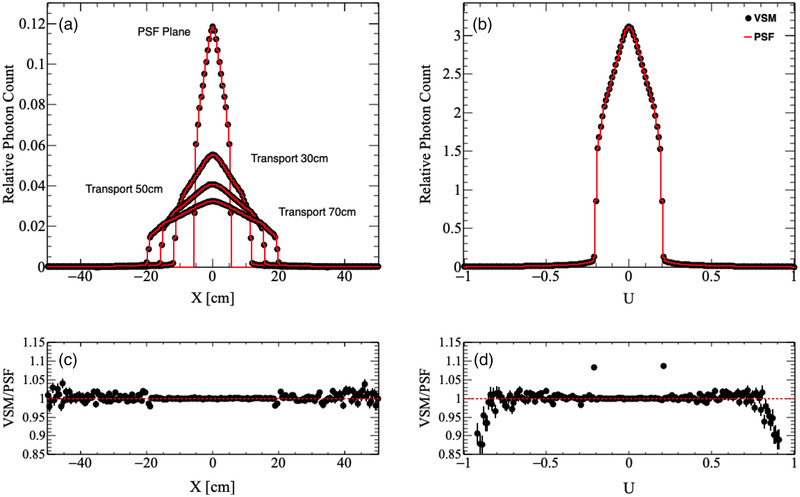
Comparison of (a) particle position and (b) direction for particles sampled using the virtual source model (VSM, black dots) to those read from phase‐space files (PSF, red lines) for the 10XFFF beam configuration. Particles were sampled at the phase‐space surface and were then transported 30, 50, and 70 cm. Ratios of the VSM to the PSF distributions for (c) particle position at the isocenter (70 cm) and (d) particle direction are shown below respective distributions

**FIGURE 6:**
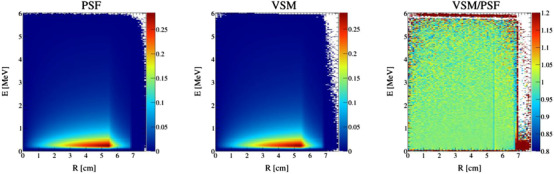
Particle energy density versus radial position for particles read from the phase‐space files (PSF, left), particles sampled from the virtual source model (VSM, middle), and the ratio of the VSM to the PSF distribution (right) for the 6XFFF beam configuration

The VSMs were first validated by simulating 100 million particles and comparing the particle distributions at the phase‐space surface (Z=26.7 cm) and isocenter (Z=100 cm) from the VSM to the particle distributions read directly from the PSF at the same locations. These tests were run outside of the MC dose framework to independently verify the quality of the simulated particles. We chose to model particle X and Y position directly over particle R with a random azimuthal angle because we observed that the particle density at the phase‐space surface was square for all beam configurations. Sampling X and Y position using the radial position with a random azimuthal angle did not capture this behavior. The results of the particle distribution comparisons for each beam configuration are shown in Table 2. Here, the *p*‐values for particle position at the phase‐space surface were larger than 0.05, but all other particle parameters had *p*‐values $<2.2\;\times 10^{-16}$, indicating significant deviations between the VSM and PSF distributions. Particle position and direction are illustrated in Figures 4 and 5 for the 10X and 10XFFF beam configurations, respectively, and particle energy density versus radial position is illustrated in Figure 6 for the 6XFFF beam configuration. We performed an analysis of the residuals between the respective VSM and PSF histograms, as seen in Figures 4, 5, 6, and found that the most significant deviations occurred at the tails of each distribution, or where the distribution is rapidly changing. These deviations, while small in magnitude (∼2%), were statistically significant. To find the regions, which were consistent between the two methods, we truncated each distribution and performed $\chi^{2}$ tests using the truncated distributions. The truncation points and fractional probability density remaining for each parameter are outlined in Table 3. The results of the truncated histogram comparisons for each beam configuration are shown in Table 4. Here, we see that for all particle parameters $\chi^{2}$ test *p*‐values are well above 0.05, indicating agreement between the VSM and PSF distributions.

**TABLE 2:** Comparison of virtual source model sampled particle parameters to phase‐space file read particle parameters. Particle positions were compared at the phase‐space surface (PSS) as well as at the treatment isocenter (ISO). Distributions over all sampled/read particles were compared using a $\chi^{2}$ test, *p*‐values from which are shown for each parameter and beam configuration. A *p*‐value less than 0.05 indicates statistically significant deviations between the two distributions

Parameter	6XFFF	6X	10XFFF	10X
X (PSS)	0.52	0.28	0.98	0.30
X (ISO)	$<2.2\times 10^{-16}$	$<2.2\times 10^{-16}$	$<2.2\times 10^{-16}$	$<2.2\times 10^{-16}$
Y (PSS)	0.44	0.41	0.98	0.41
Y (ISO)	$<2.2\times 10^{-16}$	$<2.2\times 10^{-16}$	$<2.2\times 10^{-16}$	$<2.2\times 10^{-16}$
U	$<2.2\times 10^{-16}$	$<2.2\times 10^{-16}$	$<2.2\times 10^{-16}$	$<2.2\times 10^{-16}$
V	$<2.2\times 10^{-16}$	$<2.2\times 10^{-16}$	$<2.2\times 10^{-16}$	$<2.2\times 10^{-16}$
E	$<2.2\times 10^{-16}$	$<2.2\times 10^{-16}$	$<2.2\times 10^{-16}$	$<2.2\times 10^{-16}$

**TABLE 3:** Sampling ranges for each particle parameter before and after truncation. Energy range and truncation points for 10X and 10XFFF beams are shown in brackets. The fraction of the probability density remaining after truncation is provided for each beam configuration

Parameter	Original range	Truncated range	6XFFF	6X	10XFFF	10X
X, Y (PS)	(−5.5, 5.5) cm	(−5.5, 5.5) cm	100%	100%	100%	100%
X, Y (ISO)	(−50.0, 50.5) cm	(−18.5, 18.5) cm	95%	92%	95%	90%
U, V	(−1, 1)	(−0.19, 0.19)	94%	90%	94%	88%
E	(0, 6 [10]) MeV	(0.1, 5.7 [10]) MeV	99%	99%	99%	99%

Abbreviation: ISO, isocenter.

**TABLE 4:** Comparison of truncated virtual source model sampled particle parameters to phase‐space file read particle parameters. Particle positions were compared at the phase‐space surface (PSS) as well as at the treatment isocenter (ISO). Distributions over all sampled/read particles were compared using a $\chi^{2}$ test, *p*‐values from which are shown for each parameter and beam configuration. A *p*‐value less than 0.05 indicates statistically significant deviations between the two distributions

Parameter	6XFFF	6X	10XFFF	10X
X (PSS)	0.53	0.28	0.98	0.30
X (ISO)	0.86	0.41	0.98	0.61
Y (PSS)	0.44	0.41	0.98	0.42
Y (ISO)	0.95	0.97	0.98	0.71
U	0.40	0.89	0.26	0.42
V	0.29	0.30	0.79	0.68
E	0.89	0.62	0.14	0.40

Overall, we see excellent agreement between the VSM and PSF approaches in the central regions for each particle distribution (>90% of the probability density) and deviations toward the tails of each distribution. Despite their statistical significance, the magnitude of these deviations is small and will not have a significant effect on the final dose. Further, for particle position and direction, it is likely that the small fraction of particles sampled with these extreme values will be blocked by the Linac jaws and/or MLC, and deviations to the final dose will be negligible. Particle energy distributions agree for >99% of the probability density and particles sampled with these extreme values will also have a negligible effect on the final dose. The results of the following studies will confirm these assertions.

### 3.4 VSM MC dose versus measurement validation

With the quality of the simulated particles established, the VSM were then implemented into the MC dose framework to evaluate their performance against measurements in a water phantom. Five water phantom cases were used to validate each beam configuration for a total of 20 test cases. For each case, an MC dose was calculated using the VSM method and was compared to 2D measurements in a water phantom using a gamma index analysis (3%/3 mm). The gamma index passing rates between MC and measured doses, as well as between the TPS and measured dose, for each case are shown in Table 5. Each case exhibited a gamma index pass rate >97%, indicating excellent agreement between simulation and measurement. Further, The VSM MC dose performed similarly, and occasionally better, than the TPS dose to capture the measured dose for all cases. Sample reports comparing MC dose with measurements from the MapCheck software for each case are provided in the supplemental material.

**TABLE 5:** Two‐dimensional gamma index analysis (3%/3 mm) of the virtual source model Monte Carlo (MC) and treatment planning system (TPS) doses versus measured doses for water phantom plans

Case	MC versus measured γ rate (%)	TPS versus measured γ rate (%)
Phantom 6X‐1	99.2	100
Phantom 6X‐2	98.9	99.3
Phantom 6X‐3	99.6	99.6
Phantom 6X‐4	100.0	100.0
Phantom 6X‐5	99.3	99.9
Phantom 6XFFF‐1	99.3	98.9
Phantom 6XFFF‐2	100.0	100.0
Phantom 6XFFF‐3	97.5	99.5
Phantom 6XFFF‐4	99.3	97.9
Phantom 6XFFF‐5	98.8	98.3
Phantom 10X‐1	98.7	94.4
Phantom 10X‐2	99.6	99.6
Phantom 10X‐3	99.3	99.3
Phantom 10X‐4	100.0	100.0
Phantom 10X‐5	100.0	100.0
Phantom 10XFFF‐1	97.1	98.9
Phantom 10XFFF‐2	98.2	100.0
Phantom 10XFFF‐3	99.5	97.3
Phantom 10XFFF‐4	100.0	100.0
Phantom 10XFFF‐5	100.0	100.0

### 3.5 VSM versus PSF method performance comparison

With the validity of the VSM established from water phantom measurements, studies were performed to compare the performance of the VSM method against the PSF method in speed and accuracy. Five clinical test cases were used to assess each beam configuration for a total of 20 test cases. For each case, an MC dose was calculated using both the VSM and the PSF methods, and doses were compared against each other using a 3D gamma index analysis (3%/3 mm). In addition, we performed timing tests to evaluate how the reduced file size of the VSM improved simulation time. The results of these studies are shown in Table 6. Here, it is seen there is a dramatic decrease in computation time for the VSM compared to the raw PSF reads, which were on average ∼14 times faster than the raw PSF reads, (mean time difference Δt=−1534 s, $p<2.2\;\times 10^{-16}$). Compared to cached memory reads of the PSF, the computation time of the VSM was ∼1.9 times slower (mean time difference Δt=57 s, $p<2.2\;\times 10^{-16}$). This result is not unexpected, as in this situation, the overhead from repeatedly sampling particle parameters is competing with the immense speed of cached memory reads. Further, the fully cached scenario will be extremely improbable in practice, given the large PSF size and other processes sharing resources on the machine. Future studies will be directed toward further optimizing the sampling process.

**TABLE 6:** Comparison of the performance of the virtual source model (VSM) and phase‐space file (PSF) implementations for Monte Carlo dose calculation in clinical cases. Computation time is reported for both raw file reads as well as cached memory reads for the phase‐space file implementation. Three‐dimensional gamma index passing rates were calculated between the PSF‐ and VSM‐generated Monte Carlo doses using 3%/3 mm criteria

Case	Target region	PSF time (s)	PSF cached time (s)	VSM time (s)	VSM versus PSF γ rate (%)
Clinical 6X‐1	Thoracic	1879	70	136	99.1
Clinical 6X‐2	Head/Neck	1740	54	115	98.9
Clinical 6X‐3	Thoracic	2114	69	133	94.9
Clinical 6X‐4	Head/Neck	1882	85	155	98.9
Clinical 6X‐5	Head/Neck	2166	59	121	90.8
Clinical 6XFFF‐1	Thoracic	1773	42	89	92.8
Clinical 6XFFF‐2	Head	333	19	29	100.0
Clinical 6XFFF‐3	Thoracic	1589	55	105	96.6
Clinical 6XFFF‐4	Thoracic	1595	35	81	98.2
Clinical 6XFFF‐5	Thoracic	1738	41	86	95.9
Clinical 10X‐1	Thoracic	2140	124	197	97.8
Clinical 10X‐2	Thoracic	1564	40	100	93.7
Clinical 10X‐3	Pelvic	1664	46	107	96.1
Clinical 10X‐4	Abdominal	1621	114	190	96.3
Clinical 10X‐5	Pelvic	1499	78	145	94.3
Clinical 10XFFF‐1	Thoracic	1822	42	93	99.5
Clinical 10XFFF‐2	Abdominal	1682	180	248	99.7
Clinical 10XFFF‐3	Pelvic	1750	56	110	98.9
Clinical 10XFFF‐4	Abdominal	1700	96	155	97.7
Clinical 10XFFF‐5	Abdominal	906	34	83	93.3

**FIGURE 7:**
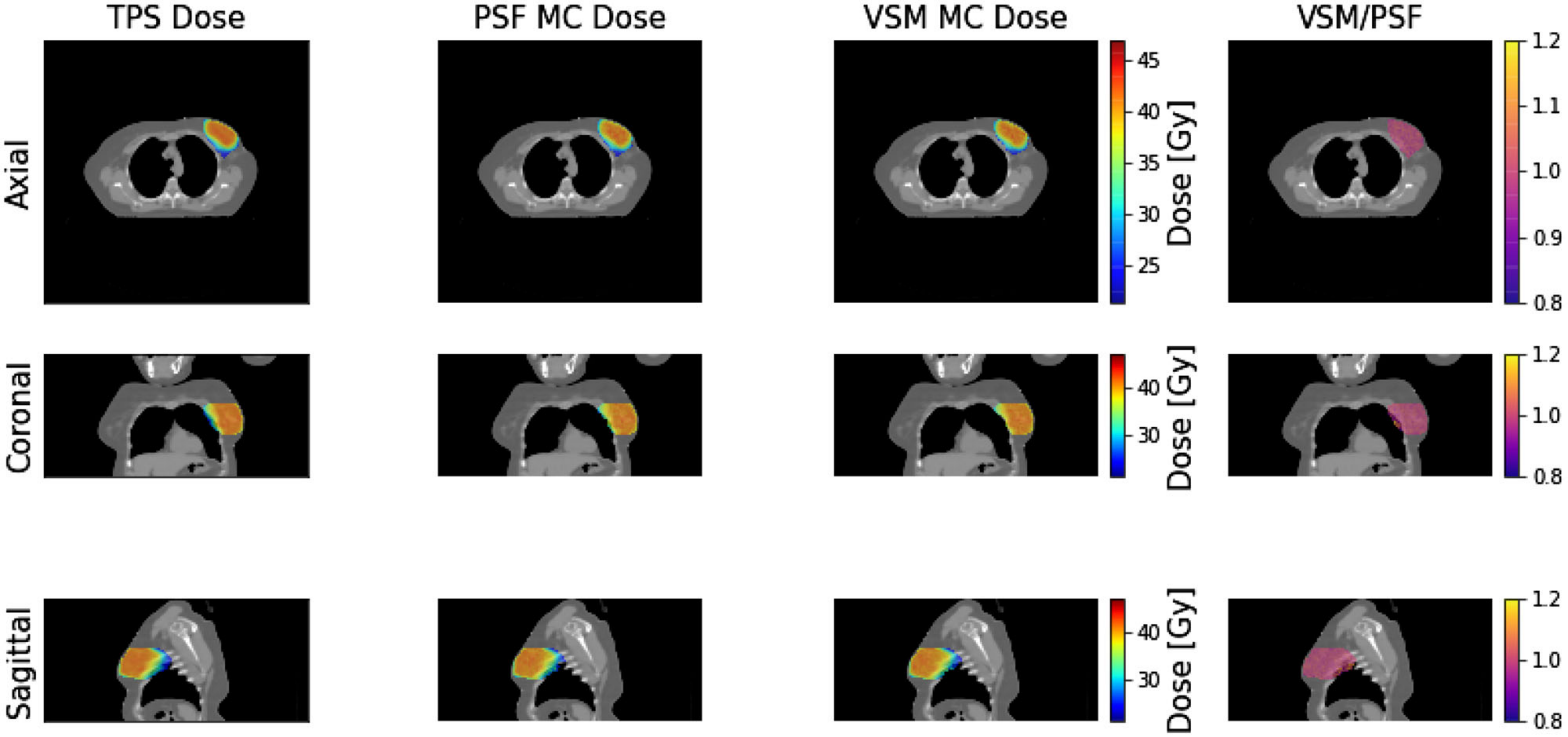
Axial, coronal, and sagittal Monte Carlo dose profiles for the “Clinical 6X‐3″ test case. The first column shows the profiles for the planned (treatment planning system [TPS]) dose, the second column shows the profiles for the phase‐space file (PSF) Monte Carlo dose, the third column shows the profiles for the virtual source model (VSM) Monte Carlo dose, and the fourth column shows the ratio of the VSM to the PSF dose

**FIGURE 8:**
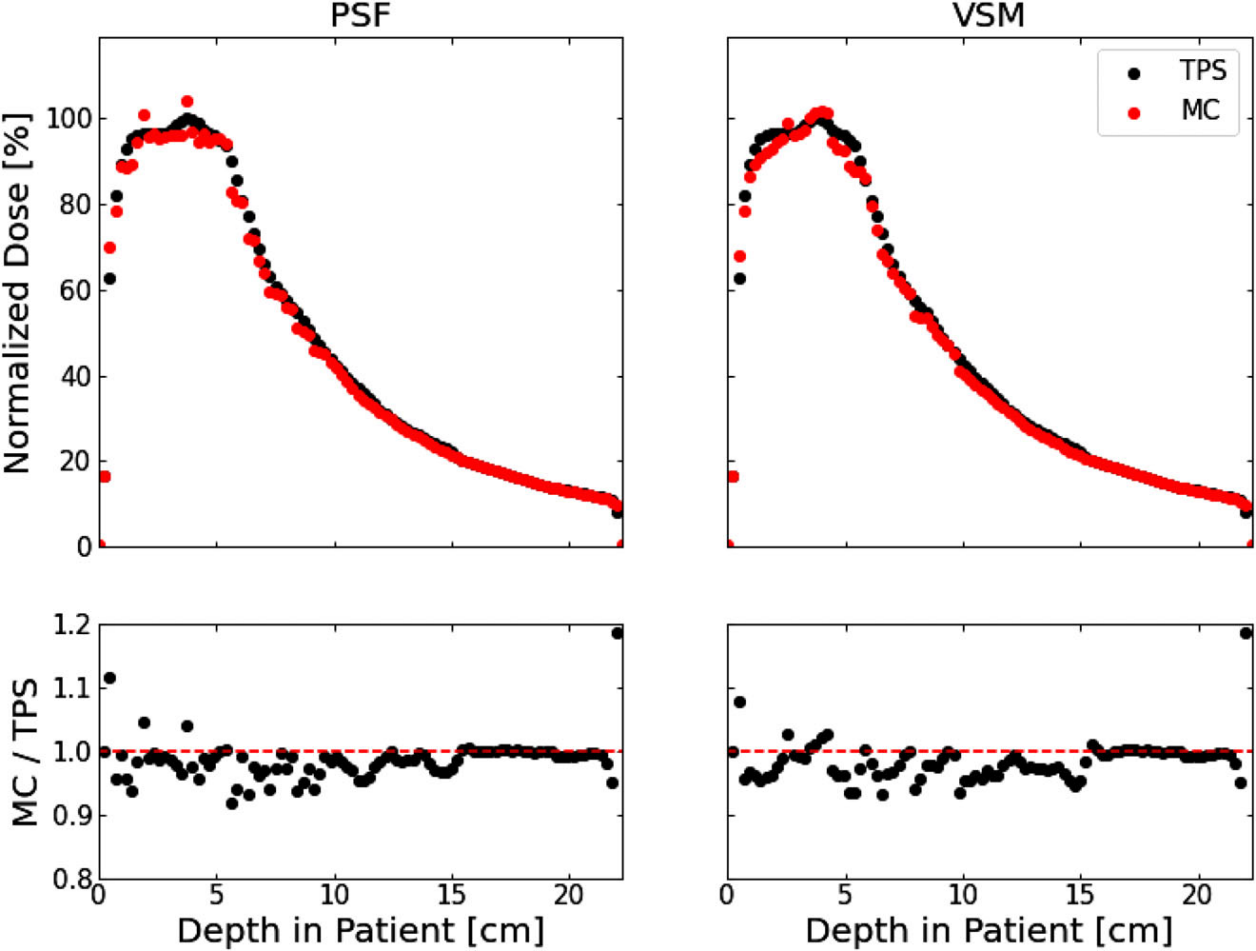
Normalized 1D dose profiles for Monte Carlo (MC) doses obtained using a phase‐space file (PSF, left) and a virtual source model (VSM, right) for the “Clinical 6X‐3″ test case. Dose profiles are presented for both the planned dose (black dots) as well as the Monte Carlo doses (red dots)

A visual comparison between MC doses obtained using the VSM and PSF implementations for the 6X beam configuration (“clinical 6X‐3″ in Table 6) is shown in Figure 7 for a clinical test case. One dimensional normalized dose profiles along the beam axis were generated for each case as well, an example of which is shown in Figure 8 for the same 6X clinical test case as in Figure 7. As expected, the MC doses calculated using the VSM and PSF implementations are very similar. Gamma index passing rates were used to quantify deviations between the two methods and were >90% for all cases, which indicates excellent agreement between the two methods. In addition, we performed an analysis of the residuals between the respective VSM and PSF doses, as seen in the fourth column of Figure 7. For cases with gamma index passing rates closer to 90%, we found that the largest discrepancies occurred near the edges of the dose distributions and near sharp changes in the dose distributions. For specific cases, discrepancies also occurred in very low‐density regions (e.g., lungs or airways). We believe that the discrepancies near the dose distribution edges arise from the oversimplification of the MLC leaf tips in our MC framework.^27^ Further, the discrepancies at sharp changes in the dose distributions could arise from how we model primary and scattered photons. It should be noted that there are still some primary photons outside the 3σ window at the treatment head (although <0.3%), and there are some scattered photons within the 3σ window (<2% based on the fits shown in Figure 3). The model can be tuned by choosing a different window (e.g., 2.5σ), but we have found that 3σ window produced the best results. Future studies will be directed toward improving the primary photon classification of our VSM. MC dose distributions overlaid with patient CT, and 1D dose profiles for all clinical test cases are available in the supplemental materials.

## 4 CONCLUSIONS

We have developed a new method for deriving VSM from IAEA PSF for Varian TrueBeam Linacs. This method is easy to implement and replace preexisting code that utilizes PSF. Extensive validation shows that the VSM sampling method, and the MC doses that arise from sampled particles, is robust and provides significant savings in both disk space usage and computation time without loss of quality results. Future studies will be directed toward further optimizing the sampling method and extending the method to more treatment machines, like Elekta (Elekta AB, Stockholm, Sweden) Linacs and Varian Halcyon.

## CONFLICT OF INTEREST

James R. Castle and Xue Feng are employed by Carina Medical LLC. Quan Chen is a shareholder of Carina Medical LLC.

## AUTHOR CONTRIBUTIONS

Xue Feng directed the study. Quan Chen provided water phantom and clinical test cases. Jingwei Duan and Quan Chen performed gamma index analyses and measurements for water phantom test cases. Quan Chen, Xue Feng, and James R. Castle developed and refined the MC dose calculation framework. James R. Castle developed, validated, and implemented the virtual source model. Xue Feng, Quan Chen, and Jingwei Duan provided valuable insight, interpretations, and advice. James R. Castle wrote the manuscript with inputs from the other authors. All authors read and approved the final manuscript.

## Supporting information

Supporting Information
