# Supplementary material for: Development of a virtual source model for Monte Carlo‐based independent dose calculation for varian linac
Source: J Appl Clin Med Phys. 2022 Feb 9;23(5):e13556. doi: 10.1002/acm2.13556 (PMC9121055; doi:10.1002/acm2.13556)
Supplement: Supplementary file 1 — Supporting Information [file ACM2-23-e13556-s001.pdf]

Manipulations

Command	Data Set	Parameters	Value
Set zero	B	LR	-0.5 mm
		TG	-0.7 mm

Gamma 2D - Parameters

3.0 mm Distance- To- Agreement
3.0 % Dose difference with ref. to max. dose of calculated volume
Use increased tolerance of 5.0 % dose diff. for values below 0.1 Gy (or AU)
Suppress dose below 10.0 % of max. dose of calculated volume
Option "Use 2nd and 3rd pass" selected

Statistics

Number of Dose Points	1,405
Evaluated Dose Points	304 (21.6 %)
Passed	304 (100.0 %)
Failed	0 (0.0 %)
Result	100.0 % (Green)

Gamma 2D

Arithmetic Mean	0.300
Min (LR = 50.0 mm; TG = -30.0 mm)	0.001
Max (LR = 75.0 mm; TG = -5.0 mm)	0.961
Median	0.249

Absolute Difference

Arithmetic Mean	0.103 Gy
Min (LR = 50.0 mm; TG = -30.0 mm)	0.000 Gy
Max (LR = 15.0 mm; TG = 25.0 mm)	0.730 Gy
Median	0.072 Gy

Settings

Passing criteria	Gamma ≤ 1.0
Green	90.0 % to 100.0 %
Yellow	75.0 % to 90.0 %
Red	0.0 % to 75.0 %

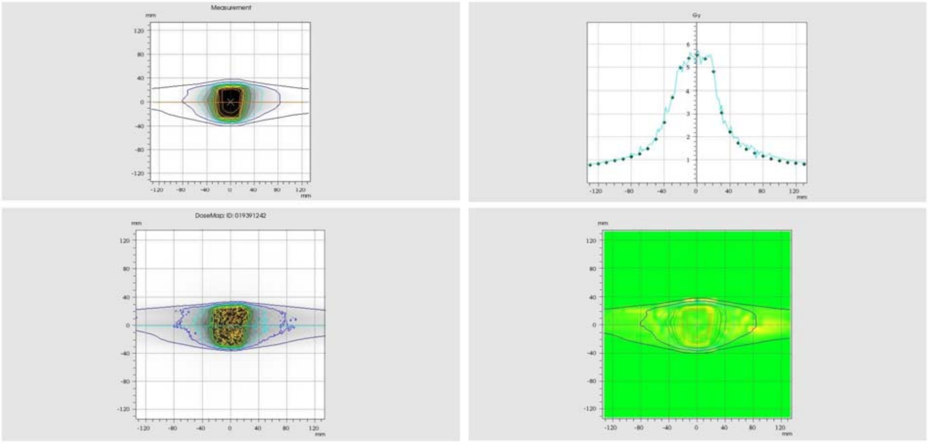

Supplemental Figure 1. Report generated by the MapCheck software comparing the virtual source model Monte Carlo dose to measurements made in a water phantom for the "Phantom 6X-1" test case.

Manipulations

Command	Data Set	Parameters	Value
Set zero	B	LR	-0.8 mm
		TG	-1.0 mm

Gamma 2D - Parameters

3.0 mm Distance- To- Agreement
3.0 % Dose difference with ref. to max. dose of calculated volume
Use increased tolerance of 5.0 % dose diff. for values below 0.1 Gy (or AU)
Suppress dose below 10.0 % of max. dose of calculated volume
Option "Use 2nd and 3rd pass" selected

Statistics

Number of Dose Points	1,405
Evaluated Dose Points	512 (36.4 %)
Passed	508 (99.2 %)
Failed	4 (0.8 %)
Result	99.2 % (Green)

Gamma 2D

Arithmetic Mean	0.315
Min (LR = -20.0 mm; TG = -10.0 mm)	0.003
Max (LR = -25.0 mm; TG = 45.0 mm)	1.358
Median	0.263

Absolute Difference

Arithmetic Mean	0.033 Gy
Min (LR = -20.0 mm; TG = -10.0 mm)	0.000 Gy
Max (LR = -20.0 mm; TG = 50.0 mm)	0.230 Gy
Median	0.020 Gy

Settings

Passing criteria	Gamma ≤ 1.0
Green	90.0 % to 100.0 %
Yellow	75.0 % to 90.0 %
Red	0.0 % to 75.0 %

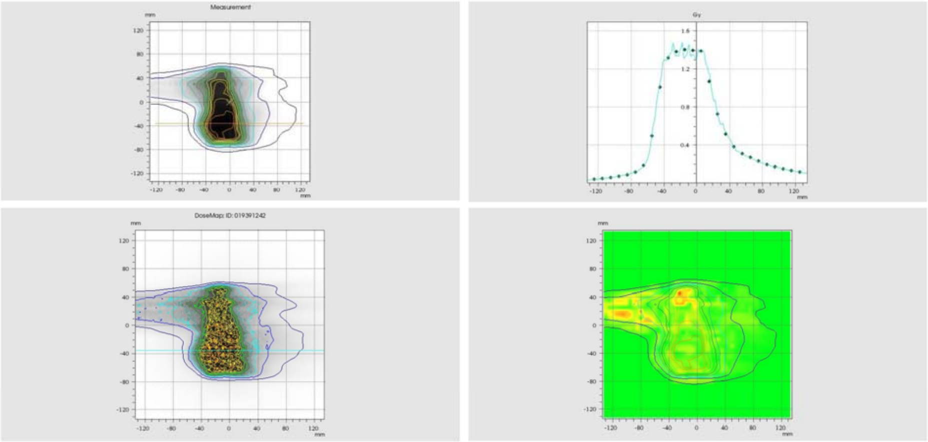

Supplemental Figure 2. Report generated by the MapCheck software comparing the virtual source model Monte Carlo dose to measurements made in a water phantom for the “Phantom 6X-2” test case.

Manipulations

Command	Data Set	Parameters	Value
Set zero	B	LR	-1.1 mm
		TG	-0.4 mm

Gamma 2D - Parameters

3.0 mm Distance- To- Agreement
3.0 % Dose difference with ref. to max. dose of calculated volume
Use increased tolerance of 5.0 % dose diff. for values below 0.1 Gy (or AU)
Suppress dose below 10.0 % of max. dose of calculated volume
Option "Use 2nd and 3rd pass" selected

Statistics

Number of Dose Points	1,405
Evaluated Dose Points	570 (40.6 %)
Passed	564 (98.9 %)
Failed	6 (1.1 %)
Result	98.9 % (Green)

Gamma 2D

Arithmetic Mean	0.326
Min (LR = 125.0 mm; TG = 35.0 mm)	0.002
Max (LR = 80.0 mm; TG = -90.0 mm)	3.167
Median	0.258

Absolute Difference

Arithmetic Mean	0.038 Gy
Min (LR = 125.0 mm; TG = 35.0 mm)	0.000 Gy
Max (LR = 5.0 mm; TG = 45.0 mm)	0.194 Gy
Median	0.030 Gy

Settings

Passing criteria	Gamma ≤ 1.0
Green	90.0 % to 100.0 %
Yellow	75.0 % to 90.0 %
Red	0.0 % to 75.0 %

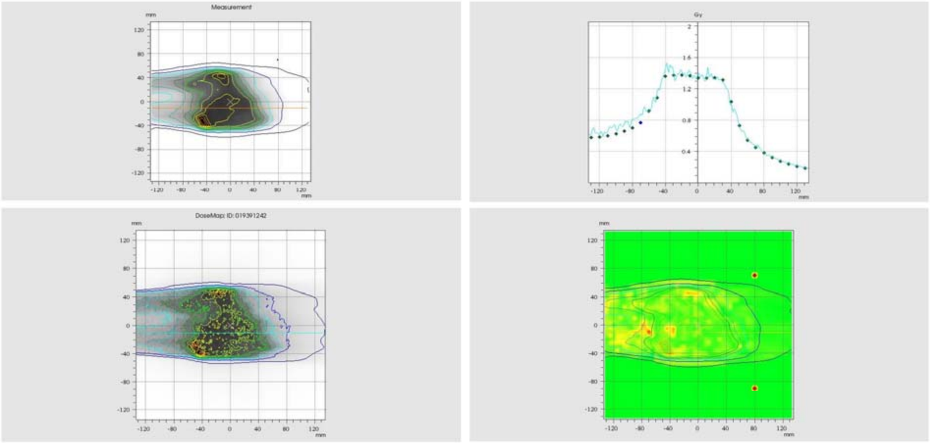

Supplemental Figure 3. Report generated by the MapCheck software comparing the virtual source model Monte Carlo dose to measurements made in a water phantom for the “Phantom 6X-3” test case.

Manipulations

Command	Data Set	Parameters	Value
Set zero	B	LR	0.5 mm
		TG	-1.0 mm

Gamma 2D - Parameters

3.0 mm Distance- To- Agreement
3.0 % Dose difference with ref. to max. dose of calculated volume
Use increased tolerance of 5.0 % dose diff. for values below 0.1 Gy (or AU)
Suppress dose below 10.0 % of max. dose of calculated volume
Option "Use 2nd and 3rd pass" selected

Statistics

Number of Dose Points	1,405
Evaluated Dose Points	276 (19.6 %)
Passed	275 (99.6 %)
Failed	1 (0.4 %)
Result	99.6 % (Green)

Gamma 2D

Arithmetic Mean	0.351
Min (LR = 75.0 mm; TG = 25.0 mm)	0.002
Max (LR = -75.1 mm; TG = 5.0 mm)	1.023
Median	0.335

Absolute Difference

Arithmetic Mean	0.190 Gy
Min (LR = 75.0 mm; TG = 25.0 mm)	0.000 Gy
Max (LR = 35.0 mm; TG = 5.0 mm)	0.858 Gy
Median	0.145 Gy

Settings

Passing criteria	Gamma ≤ 1.0
Green	90.0 % to 100.0 %
Yellow	75.0 % to 90.0 %
Red	0.0 % to 75.0 %

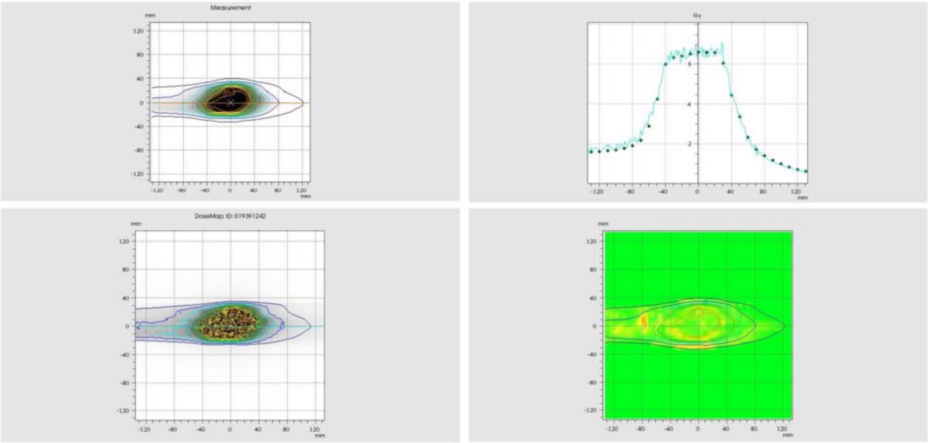

Supplemental Figure 4. Report generated by the MapCheck software comparing the virtual source model Monte Carlo dose to measurements made in a water phantom for the "Phantom 6X-4" test case.

Manipulations

Command	Data Set	Parameters	Value
Set zero	B	LR	-0.8 mm
		TG	-0.6 mm

Gamma 2D - Parameters

3.0 mm Distance- To- Agreement
3.0 % Dose difference with ref. to max. dose of calculated volume
Use increased tolerance of 5.0 % dose diff. for values below 0.1 Gy (or AU)
Suppress dose below 10.0 % of max. dose of calculated volume
Option "Use 2nd and 3rd pass" selected

Statistics

Number of Dose Points	1,405
Evaluated Dose Points	531 (37.8 %)
Passed	531 (100.0 %)
Failed	0 (0.0 %)
Result	100.0 % (Green)

Gamma 2D

Arithmetic Mean	0.258
Min (LR = -95.0 mm; TG = -15.0 mm)	0.002
Max (LR = -5.0 mm; TG = 55.0 mm)	0.881
Median	0.217

Absolute Difference

Arithmetic Mean	0.031 Gy
Min (LR = -95.0 mm; TG = -15.0 mm)	0.000 Gy
Max (LR = 0.0 mm; TG = -50.0 mm)	0.199 Gy
Median	0.022 Gy

Settings

Passing criteria	Gamma ≤ 1.0
Green	90.0 % to 100.0 %
Yellow	75.0 % to 90.0 %
Red	0.0 % to 75.0 %

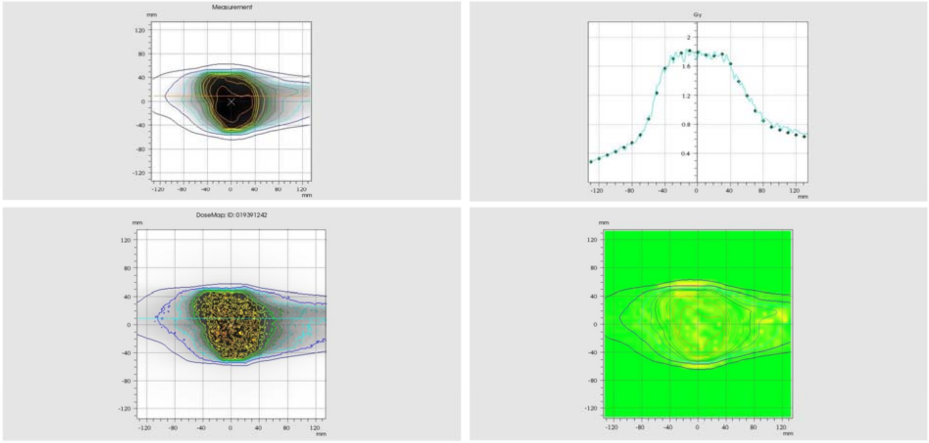

Supplemental Figure 5. Report generated by the MapCheck software comparing the virtual source model Monte Carlo dose to measurements made in a water phantom for the "Phantom 6X-5" test case.

Manipulations

Command	Data Set	Parameters	Value
Set zero	B	LR	-0.3 mm
		TG	-0.7 mm

Gamma 2D - Parameters

3.0 mm Distance- To- Agreement
3.0 % Dose difference with ref. to max. dose of calculated volume
Use increased tolerance of 5.0 % dose diff. for values below 0.1 Gy (or AU)
Suppress dose below 10.0 % of max. dose of calculated volume
Option "Use 2nd and 3rd pass" selected

Statistics

Number of Dose Points	1,405
Evaluated Dose Points	131 (9.3 %)
Passed	130 (99.2 %)
Failed	1 (0.8 %)
Result	99.2 % (Green)

Gamma 2D

Arithmetic Mean	0.321
Min (LR = -60.0 mm; TG = -10.0 mm)	0.000
Max (LR = 50.0 mm; TG = 0.0 mm)	1.032
Median	0.283

Absolute Difference

Arithmetic Mean	0.178 Gy
Min (LR = -60.0 mm; TG = -10.0 mm)	0.000 Gy
Max (LR = -5.0 mm; TG = 15.0 mm)	0.930 Gy
Median	0.127 Gy

Settings

Passing criteria	Gamma ≤ 1.0
Green	90.0 % to 100.0 %
Yellow	75.0 % to 90.0 %
Red	0.0 % to 75.0 %

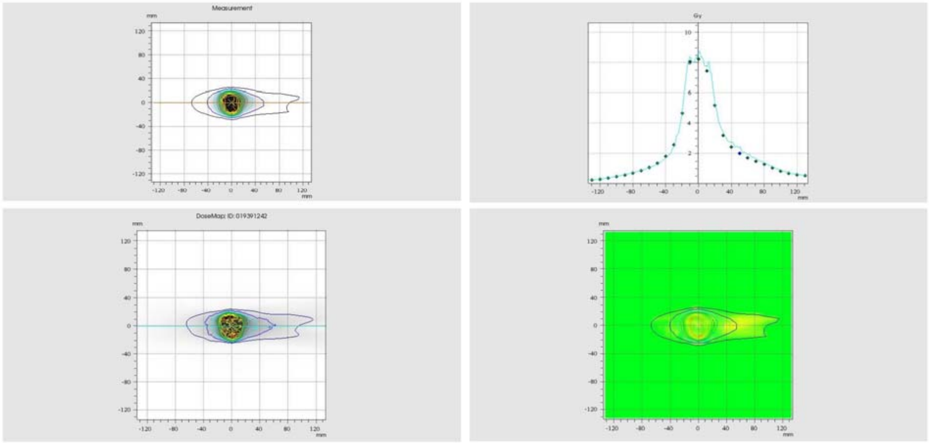

Supplemental Figure 6. Report generated by the MapCheck software comparing the virtual source model Monte Carlo dose to measurements made in a water phantom for the “Phantom 6XFFF-1” test case.

Manipulations

Command	Data Set	Parameters	Value
Set zero	B	LR	-0.9 mm
		TG	-0.6 mm

Gamma 2D - Parameters

3.0 mm Distance- To- Agreement
3.0 % Dose difference with ref. to max. dose of calculated volume
Use increased tolerance of 5.0 % dose diff. for values below 0.1 Gy (or AU)
Suppress dose below 10.0 % of max. dose of calculated volume
Option "Use 2nd and 3rd pass" selected

Statistics

Number of Dose Points	1,405
Evaluated Dose Points	459 (32.7 %)
Passed	456 (99.3 %)
Failed	3 (0.7 %)
Result	99.3 % (Green)

Gamma 2D

Arithmetic Mean	0.344
Min (LR = 30.0 mm; TG = 20.0 mm)	0.000
Max (LR = -55.0 mm; TG = -15.0 mm)	1.117
Median	0.323

Absolute Difference

Arithmetic Mean	0.130 Gy
Min (LR = 30.0 mm; TG = 20.0 mm)	0.000 Gy
Max (LR = -40.0 mm; TG = 30.0 mm)	0.556 Gy
Median	0.114 Gy

Settings

Passing criteria	Gamma ≤ 1.0
Green	90.0 % to 100.0 %
Yellow	75.0 % to 90.0 %
Red	0.0 % to 75.0 %

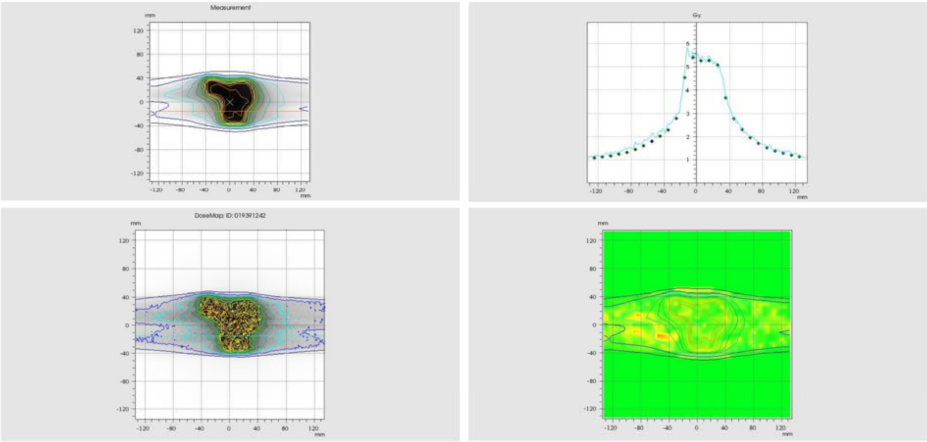

Supplemental Figure 7. Report generated by the MapCheck software comparing the virtual source model Monte Carlo dose to measurements made in a water phantom for the "Phantom 6XFFF-2" test case.

Manipulations

Command	Data Set	Parameters	Value
Set zero	B	LR	0.2 mm
		TG	-0.2 mm

Gamma 2D - Parameters

3.0 mm Distance- To- Agreement
3.0 % Dose difference with ref. to max. dose of calculated volume
Use increased tolerance of 5.0 % dose diff. for values below 0.1 Gy (or AU)
Suppress dose below 10.0 % of max. dose of calculated volume
Option "Use 2nd and 3rd pass" selected

Statistics

Number of Dose Points	1,405
Evaluated Dose Points	226 (16.1 %)
Passed	226 (100.0 %)
Failed	0 (0.0 %)
Result	100.0 % (Green)

Gamma 2D

Arithmetic Mean	0.270
Min (LR = -50.0 mm; TG = -20.0 mm)	0.012
Max (LR = 0.0 mm; TG = 10.0 mm)	0.956
Median	0.234

Absolute Difference

Arithmetic Mean	0.140 Gy
Min (LR = -50.0 mm; TG = -20.0 mm)	0.003 Gy
Max (LR = 0.0 mm; TG = 20.0 mm)	1.126 Gy
Median	0.076 Gy

Settings

Passing criteria	Gamma ≤ 1.0
Green	90.0 % to 100.0 %
Yellow	75.0 % to 90.0 %
Red	0.0 % to 75.0 %

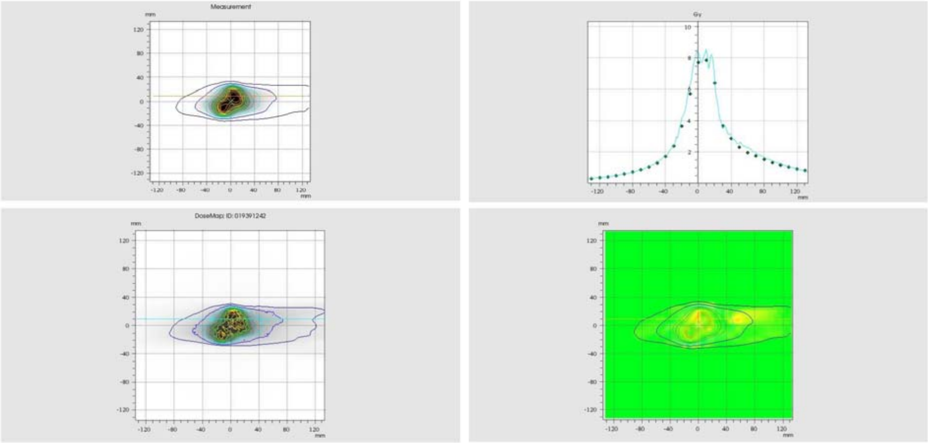

Supplemental Figure 8. Report generated by the MapCheck software comparing the virtual source model Monte Carlo dose to measurements made in a water phantom for the "Phantom 6XFFF-3" test case.

Manipulations

Command	Data Set	Parameters	Value

Gamma 2D - Parameters

3.0 mm Distance- To- Agreement
3.0 % Dose difference with ref. to max. dose of calculated volume
Use increased tolerance of 5.0 % dose diff. for values below 0.1 Gy (or AU)
Suppress dose below 10.0 % of max. dose of calculated volume
Option "Use 2nd and 3rd pass" selected

Statistics

Number of Dose Points	1,405
Evaluated Dose Points	198 (14.1 %)
Passed	193 (97.5 %)
Failed	5 (2.5 %)
Result	97.5 % (Green)

Gamma 2D

Arithmetic Mean	0.308
Min (LR = 105.0 mm; TG = -25.0 mm)	0.001
Max (LR = 5.0 mm; TG = 35.0 mm)	5.105
Median	0.195

Absolute Difference

Arithmetic Mean	0.164 Gy
Min (LR = 105.0 mm; TG = -25.0 mm)	0.000 Gy
Max (LR = 5.0 mm; TG = 35.0 mm)	1.401 Gy
Median	0.067 Gy

Settings

Passing criteria	Gamma ≤ 1.0
Green	90.0 % to 100.0 %
Yellow	75.0 % to 90.0 %
Red	0.0 % to 75.0 %

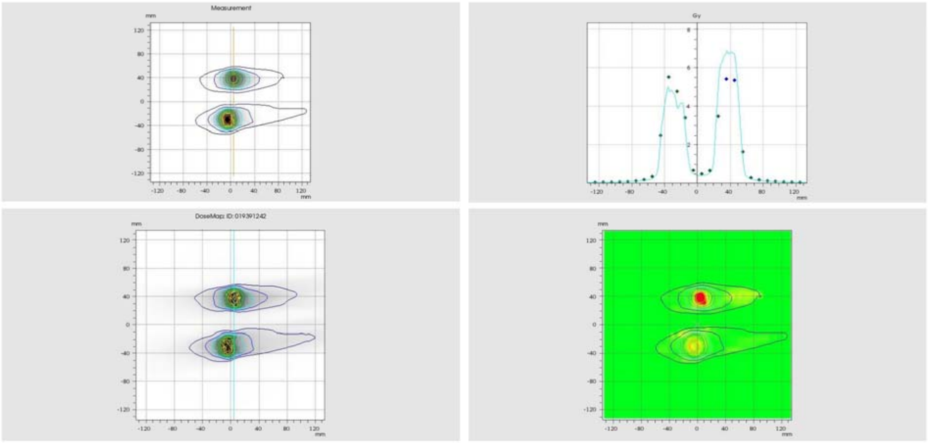

Supplemental Figure 9. Report generated by the MapCheck software comparing the virtual source model Monte Carlo dose to measurements made in a water phantom for the “Phantom 6XFFF-4” test case.

Manipulations

Command	Data Set	Parameters	Value
Set zero	B	LR	0.1 mm
		TG	-1.1 mm

Gamma 2D - Parameters

3.0 mm Distance- To- Agreement
3.0 % Dose difference with ref. to max. dose of calculated volume
Use increased tolerance of 5.0 % dose diff. for values below 0.1 Gy (or AU)
Suppress dose below 10.0 % of max. dose of calculated volume
Option "Use 2nd and 3rd pass" selected

Statistics

Number of Dose Points	1,405
Evaluated Dose Points	291 (20.7 %)
Passed	289 (99.3 %)
Failed	2 (0.7 %)
Result	99.3 % ● (Green)

Gamma 2D

Arithmetic Mean	0.316
Min (LR = -15.0 mm; TG = -5.0 mm)	0.001
Max (LR = -10.0 mm; TG = 20.0 mm)	1.165
Median	0.289

Absolute Difference

Arithmetic Mean	0.235 Gy
Min (LR = -15.0 mm; TG = -5.0 mm)	0.000 Gy
Max (LR = -15.0 mm; TG = -25.0 mm)	1.642 Gy
Median	0.152 Gy

Settings

Passing criteria	Gamma ≤ 1.0
Green	90.0 % to 100.0 %
Yellow	75.0 % to 90.0 %
Red	0.0 % to 75.0 %

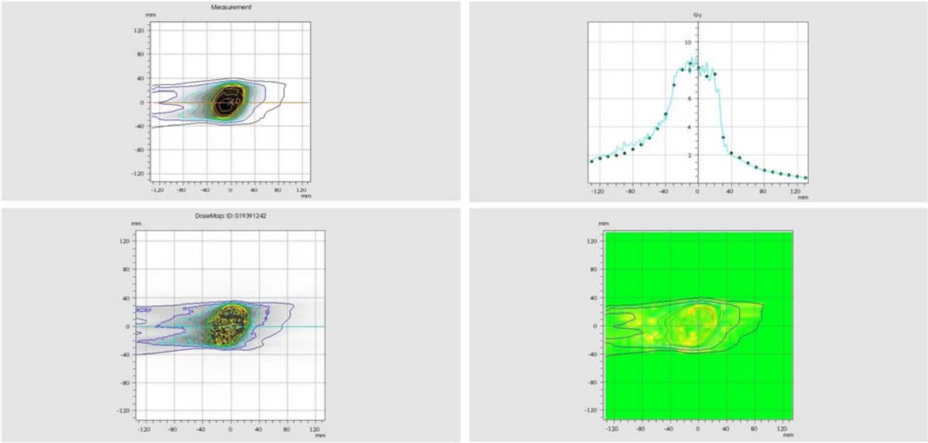

Supplemental Figure 10. Report generated by the MapCheck software comparing the virtual source model Monte Carlo dose to measurements made in a water phantom for the "Phantom 6XFFF-5" test case.

Manipulations

Command	Data Set	Parameters	Value
Set zero	B	LR	-0.2 mm
		TG	-1.0 mm

Gamma 2D - Parameters

3.0 mm Distance- To- Agreement
3.0 % Dose difference with ref. to max. dose of calculated volume
Use increased tolerance of 5.0 % dose diff. for values below 0.1 Gy (or AU)
Suppress dose below 10.0 % of max. dose of calculated volume
Option "Use 2nd and 3rd pass" selected

Statistics

Number of Dose Points	1,405
Evaluated Dose Points	994 (70.7 %)
Passed	981 (98.7 %)
Failed	13 (1.3 %)
Result	98.7 % (Green)

Gamma 2D

Arithmetic Mean	0.309
Min (LR = -10.0 mm; TG = -20.0 mm)	0.000
Max (LR = 130.0 mm; TG = 90.0 mm)	1.685
Median	0.262

Absolute Difference

Arithmetic Mean	0.057 Gy
Min (LR = -10.0 mm; TG = -20.0 mm)	0.000 Gy
Max (LR = -30.0 mm; TG = -80.0 mm)	0.422 Gy
Median	0.040 Gy

Settings

Passing criteria	Gamma ≤ 1.0
Green	90.0 % to 100.0 %
Yellow	75.0 % to 90.0 %
Red	0.0 % to 75.0 %

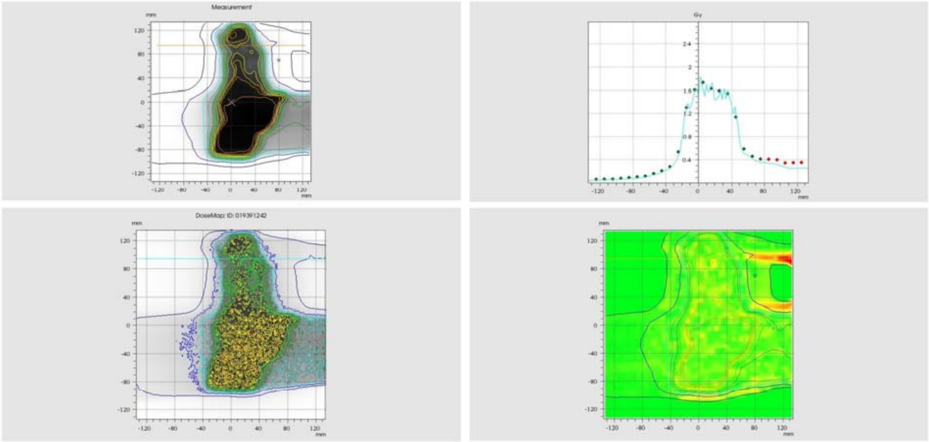

Supplemental Figure 11. Report generated by the MapCheck software comparing the virtual source model Monte Carlo dose to measurements made in a water phantom for the “Phantom 10X-1” test case.

Manipulations

Command	Data Set	Parameters	Value
Set zero	B	LR	-1.9 mm
		TG	-0.3 mm

Gamma 2D - Parameters

3.0 mm Distance- To- Agreement
3.0 % Dose difference with ref. to max. dose of calculated volume
Use increased tolerance of 5.0 % dose diff. for values below 0.1 Gy (or AU)
Suppress dose below 10.0 % of max. dose of calculated volume
Option "Use 2nd and 3rd pass" selected

Statistics

Number of Dose Points	1,405
Evaluated Dose Points	245 (17.4 %)
Passed	244 (99.6 %)
Failed	1 (0.4 %)
Result	99.6 % (Green)

Gamma 2D

Arithmetic Mean	0.319
Min (LR = 105.0 mm; TG = -5.0 mm)	0.010
Max (LR = 80.0 mm; TG = 70.0 mm)	5.829
Median	0.268

Absolute Difference

Arithmetic Mean	0.031 Gy
Min (LR = 105.0 mm; TG = -5.0 mm)	0.000 Gy
Max (LR = 80.0 mm; TG = 70.0 mm)	0.210 Gy
Median	0.021 Gy

Settings

Passing criteria	Gamma ≤ 1.0
Green	90.0 % to 100.0 %
Yellow	75.0 % to 90.0 %
Red	0.0 % to 75.0 %

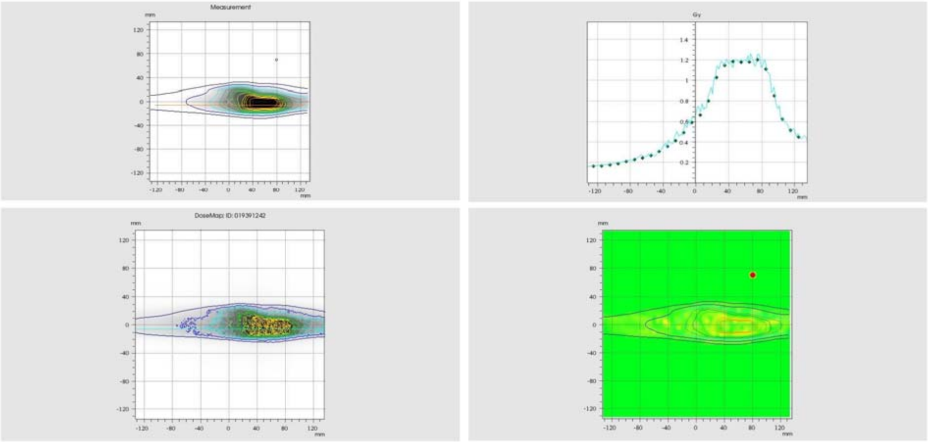

Supplemental Figure 12. Report generated by the MapCheck software comparing the virtual source model Monte Carlo dose to measurements made in a water phantom for the "Phantom 10X-2" test case.

Manipulations

Command	Data Set	Parameters	Value
Set zero	B	LR	-1.3 mm
		TG	-0.9 mm

Gamma 2D - Parameters

3.0 mm Distance- To- Agreement
3.0 % Dose difference with ref. to max. dose of calculated volume
Use increased tolerance of 5.0 % dose diff. for values below 0.1 Gy (or AU)
Suppress dose below 10.0 % of max. dose of calculated volume
Option "Use 2nd and 3rd pass" selected

Statistics

Number of Dose Points	1,405
Evaluated Dose Points	1,009 (71.8 %)
Passed	1,002 (99.3 %)
Failed	7 (0.7 %)
Result	99.3 % (Green)

Gamma 2D

Arithmetic Mean	0.270
Min (LR = 90.0 mm; TG = -80.0 mm)	0.000
Max (LR = -10.0 mm; TG = 100.0 mm)	1.662
Median	0.221

Absolute Difference

Arithmetic Mean	0.040 Gy
Min (LR = 90.0 mm; TG = -80.0 mm)	0.000 Gy
Max (LR = -15.0 mm; TG = 105.0 mm)	0.261 Gy
Median	0.027 Gy

Settings

Passing criteria	Gamma ≤ 1.0
Green	90.0 % to 100.0 %
Yellow	75.0 % to 90.0 %
Red	0.0 % to 75.0 %

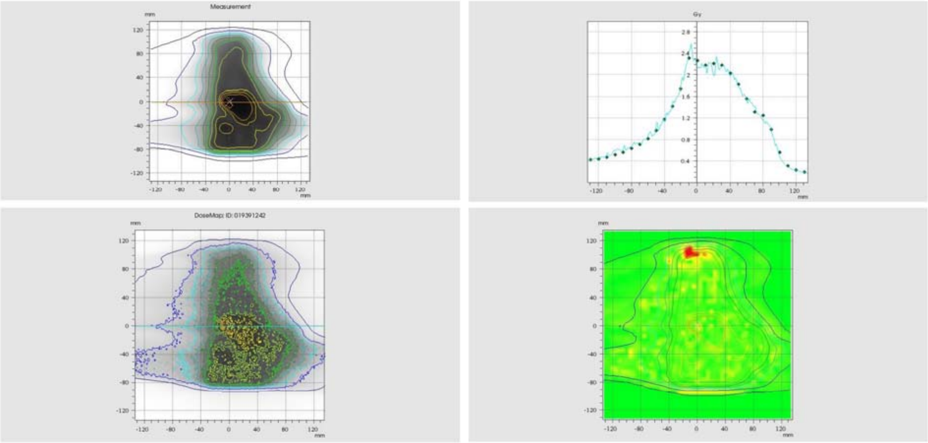

Supplemental Figure 13. Report generated by the MapCheck software comparing the virtual source model Monte Carlo dose to measurements made in a water phantom for the “Phantom 10X-3” test case.

Manipulations

Command	Data Set	Parameters	Value
Set zero	B	LR	-1.4 mm
		TG	-1.3 mm

Gamma 2D - Parameters

3.0 mm Distance- To- Agreement
3.0 % Dose difference with ref. to max. dose of calculated volume
Use increased tolerance of 5.0 % dose diff. for values below 0.1 Gy (or AU)
Suppress dose below 10.0 % of max. dose of calculated volume
Option "Use 2nd and 3rd pass" selected

Statistics

Number of Dose Points	1,405
Evaluated Dose Points	412 (29.3 %)
Passed	412 (100.0 %)
Failed	0 (0.0 %)
Result	100.0 % (Green)

Gamma 2D

Arithmetic Mean	0.241
Min (LR = 65.0 mm; TG = 15.0 mm)	0.001
Max (LR = -120.0 mm; TG = 10.0 mm)	0.931
Median	0.203

Absolute Difference

Arithmetic Mean	0.055 Gy
Min (LR = 65.0 mm; TG = 15.0 mm)	0.000 Gy
Max (LR = 10.0 mm; TG = -20.0 mm)	0.392 Gy
Median	0.035 Gy

Settings

Passing criteria	Gamma ≤ 1.0
Green	90.0 % to 100.0 %
Yellow	75.0 % to 90.0 %
Red	0.0 % to 75.0 %

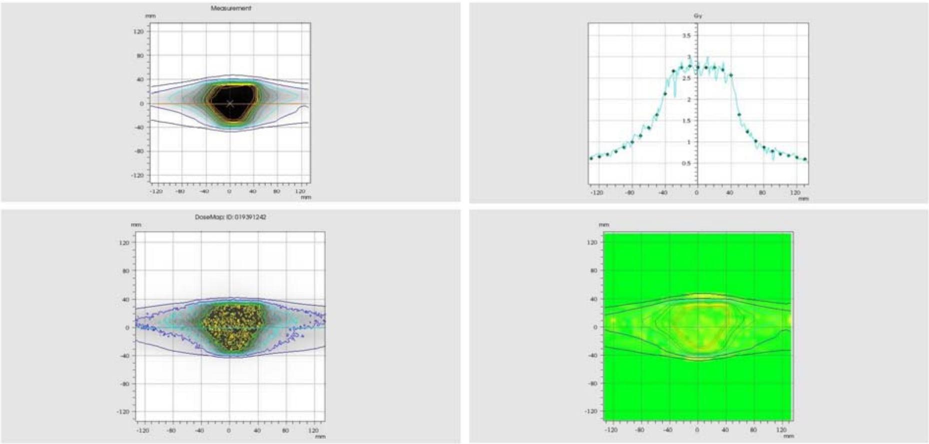

Supplemental Figure 14. Report generated by the MapCheck software comparing the virtual source model Monte Carlo dose to measurements made in a water phantom for the "Phantom 10X-4" test case.

Manipulations

Command	Data Set	Parameters	Value

Gamma 2D - Parameters

3.0 mm Distance- To- Agreement
3.0 % Dose difference with ref. to max. dose of calculated volume
Use increased tolerance of 5.0 % dose diff. for values below 0.1 Gy (or AU)
Suppress dose below 10.0 % of max. dose of calculated volume
Option "Use 2nd and 3rd pass" selected

Statistics

Number of Dose Points	1,405
Evaluated Dose Points	1,185 (84.3 %)
Passed	1,185 (100.0 %)
Failed	0 (0.0 %)
Result	100.0 % (Green)

Gamma 2D

Arithmetic Mean	0.265
Min (LR = -15.0 mm; TG = -5.0 mm)	0.000
Max (LR = 30.0 mm; TG = -60.0 mm)	0.813
Median	0.237

Absolute Difference

Arithmetic Mean	0.035 Gy
Min (LR = -15.0 mm; TG = -5.0 mm)	0.000 Gy
Max (LR = 0.0 mm; TG = 50.0 mm)	0.207 Gy
Median	0.027 Gy

Settings

Passing criteria	Gamma ≤ 1.0
Green	90.0 % to 100.0 %
Yellow	75.0 % to 90.0 %
Red	0.0 % to 75.0 %

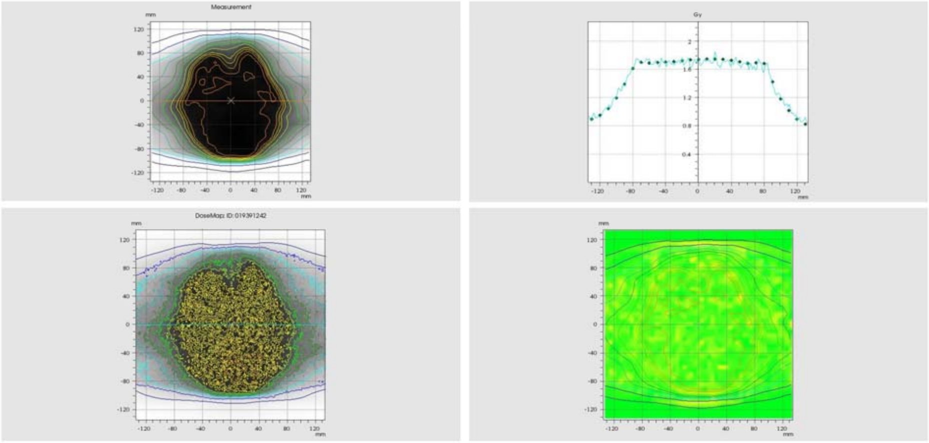

Supplemental Figure 15. Report generated by the MapCheck software comparing the virtual source model Monte Carlo dose to measurements made in a water phantom for the “Phantom 10X-5” test case.

Manipulations<br>Command	Data Set	Parameters	Value
Set zero	B	LR	-0.5 mm
		TG	0.3 mm

Gamma 2D - Parameters

3.0 mm Distance- To- Agreement
3.0 % Dose difference with ref. to max. dose of calculated volume
Use increased tolerance of 5.0 % dose diff. for values below 0.1 Gy (or AU)
Suppress dose below 10.0 % of max. dose of calculated volume
Option "Use 2nd and 3rd pass" selected

Statistics	
Number of Dose Points	1,405
Evaluated Dose Points	1,400 (99.6 %)
Passed	1,360 (97.1 %)
Failed	40 (2.9 %)
Result	97.1 % (Green)

Gamma 2D	
Arithmetic Mean	0.437
Min (LR = -95.0 mm; TG = -125.0 mm)	0.000
Max (LR = 120.0 mm; TG = 110.0 mm)	1.849
Median	0.391

Absolute Difference	
Arithmetic Mean	0.072 Gy
Min (LR = -95.0 mm; TG = -125.0 mm)	0.000 Gy
Max (LR = 40.0 mm; TG = 0.0 mm)	0.329 Gy
Median	0.064 Gy

Settings	
Passing criteria	Gamma ≤ 1.0
Green	90.0 % to 100.0 %
Yellow	75.0 % to 90.0 %
Red	0.0 % to 75.0 %

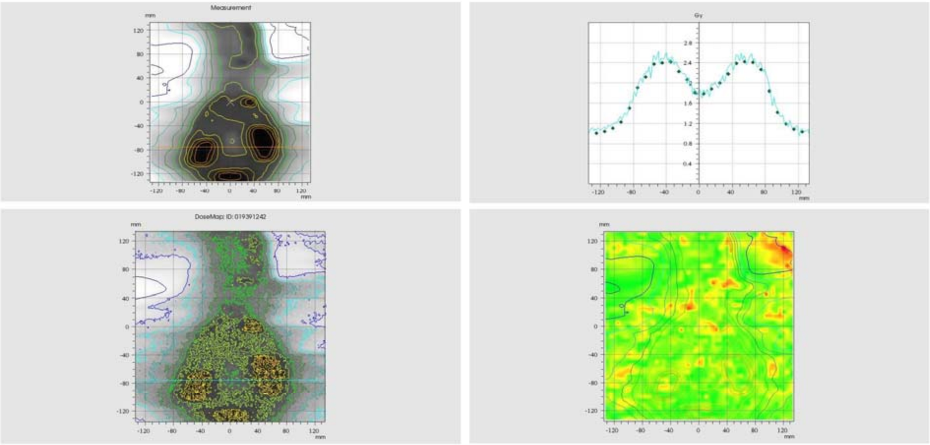

Supplemental Figure 16. Report generated by the MapCheck software comparing the virtual source model Monte Carlo dose to measurements made in a water phantom for the “Phantom 10XFFF-1” test case.

Manipulations

Command	Data Set	Parameters	Value
Set zero	B	LR	-0.7 mm
		TG	-0.7 mm

Gamma 2D - Parameters

3.0 mm Distance- To- Agreement
3.0 % Dose difference with ref. to max. dose of calculated volume
Use increased tolerance of 5.0 % dose diff. for values below 0.1 Gy (or AU)
Suppress dose below 10.0 % of max. dose of calculated volume
Option "Use 2nd and 3rd pass" selected

Statistics

Number of Dose Points	1,405
Evaluated Dose Points	494 (35.2 %)
Passed	485 (98.2 %)
Failed	9 (1.8 %)
Result	98.2 % (Green)

Gamma 2D

Arithmetic Mean	0.331
Min (LR = -130.0 mm; TG = -20.0 mm)	0.000
Max (LR = -45.0 mm; TG = -5.0 mm)	2.667
Median	0.269

Absolute Difference

Arithmetic Mean	0.046 Gy
Min (LR = -130.0 mm; TG = -20.0 mm)	0.000 Gy
Max (LR = -45.0 mm; TG = -5.0 mm)	0.285 Gy
Median	0.023 Gy

Settings

Passing criteria	Gamma ≤ 1.0
Green	90.0 % to 100.0 %
Yellow	75.0 % to 90.0 %
Red	0.0 % to 75.0 %

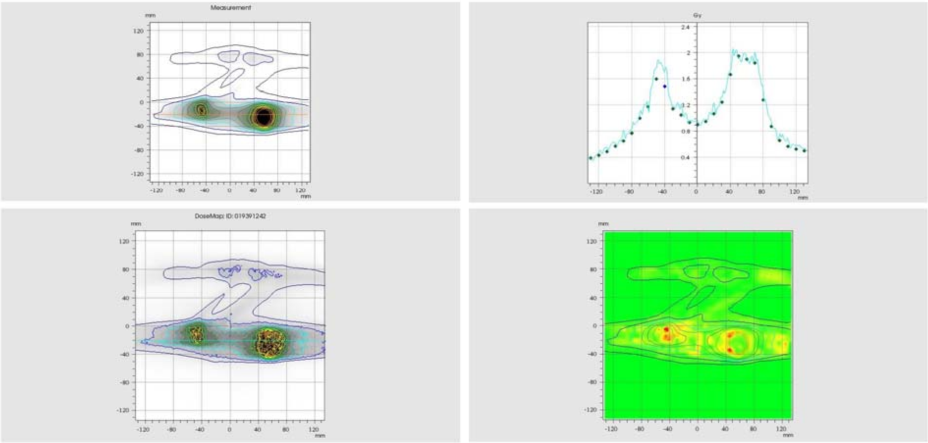

Supplemental Figure 17. Report generated by the MapCheck software comparing the virtual source model Monte Carlo dose to measurements made in a water phantom for the “Phantom 10XFFF-2” test case.

Manipulations

Command	Data Set	Parameters	Value
Set zero	B	LR	-0.5 mm
		TG	-1.1 mm

Gamma 2D - Parameters

3.0 mm Distance- To- Agreement
3.0 % Dose difference with ref. to max. dose of calculated volume
Use increased tolerance of 5.0 % dose diff. for values below 0.1 Gy (or AU)
Suppress dose below 10.0 % of max. dose of calculated volume
Option "Use 2nd and 3rd pass" selected

Statistics

Number of Dose Points	1,405
Evaluated Dose Points	185 (13.2 %)
Passed	184 (99.5 %)
Failed	1 (0.5 %)
Result	99.5 % (Green)

Gamma 2D

Arithmetic Mean	0.317
Min (LR = -70.0 mm; TG = -10.0 mm)	0.008
Max (LR = 55.0 mm; TG = -5.0 mm)	1.052
Median	0.309

Absolute Difference

Arithmetic Mean	0.120 Gy
Min (LR = -70.0 mm; TG = -10.0 mm)	0.001 Gy
Max (LR = -25.0 mm; TG = 15.0 mm)	0.535 Gy
Median	0.091 Gy

Settings

Passing criteria	Gamma ≤ 1.0
Green	90.0 % to 100.0 %
Yellow	75.0 % to 90.0 %
Red	0.0 % to 75.0 %

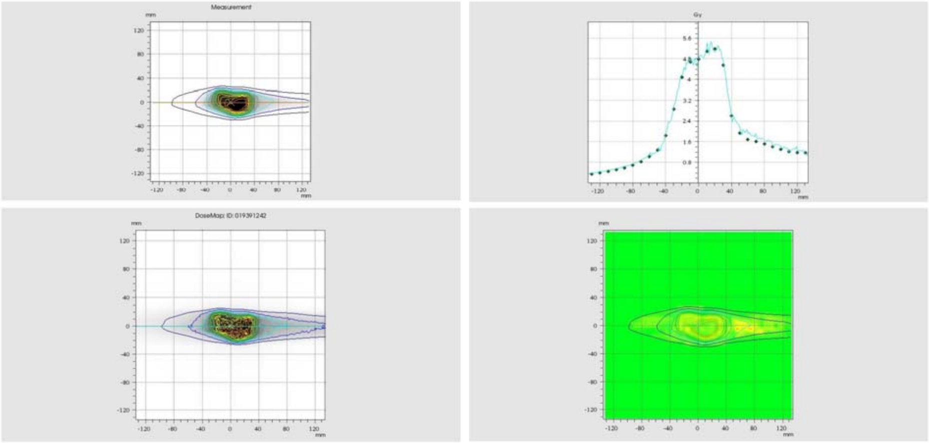

Supplemental Figure 18. Report generated by the MapCheck software comparing the virtual source model Monte Carlo dose to measurements made in a water phantom for the “Phantom 10XFFF-3” test case.

Manipulations

Command	Data Set	Parameters	Value
Set zero	B	LR	-0.4 mm
		TG	-1.3 mm

Gamma 2D - Parameters

3.0 mm Distance- To- Agreement
3.0 % Dose difference with ref. to max. dose of calculated volume
Use increased tolerance of 5.0 % dose diff. for values below 0.1 Gy (or AU)
Suppress dose below 10.0 % of max. dose of calculated volume
Option "Use 2nd and 3rd pass" selected

Statistics

Number of Dose Points	1,405
Evaluated Dose Points	223 (15.9 %)
Passed	223 (100.0 %)
Failed	0 (0.0 %)
Result	100.0 % (Green)

Gamma 2D

Arithmetic Mean	0.215
Min (LR = 95.0 mm; TG = -5.0 mm)	0.000
Max (LR = 10.0 mm; TG = -10.0 mm)	0.761
Median	0.185

Absolute Difference

Arithmetic Mean	0.180 Gy
Min (LR = 95.0 mm; TG = -5.0 mm)	0.000 Gy
Max (LR = 10.0 mm; TG = -20.0 mm)	1.173 Gy
Median	0.096 Gy

Settings

Passing criteria	Gamma ≤ 1.0
Green	90.0 % to 100.0 %
Yellow	75.0 % to 90.0 %
Red	0.0 % to 75.0 %

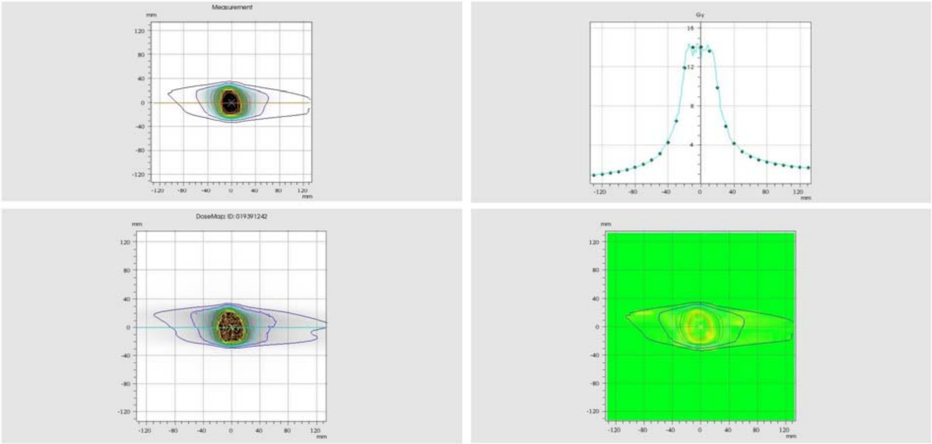

Supplemental Figure 19. Report generated by the MapCheck software comparing the virtual source model Monte Carlo dose to measurements made in a water phantom for the “Phantom 10XFFF-4” test case.

Manipulations

Command	Data Set	Parameters	Value
Set zero	B	LR	0.3 mm
		TG	-0.6 mm

Gamma 2D - Parameters

3.0 mm Distance- To- Agreement
3.0 % Dose difference with ref. to max. dose of calculated volume
Use increased tolerance of 5.0 % dose diff. for values below 0.1 Gy (or AU)
Suppress dose below 10.0 % of max. dose of calculated volume
Option "Use 2nd and 3rd pass" selected

Statistics

Number of Dose Points	1,405
Evaluated Dose Points	84 (6.0 %)
Passed	84 (100.0 %)
Failed	0 (0.0 %)
Result	100.0 % (Green)

Gamma 2D

Arithmetic Mean	0.259
Min (LR = 35.0 mm; TG = 15.0 mm)	0.020
Max (LR = -5.0 mm; TG = 15.0 mm)	0.609
Median	0.243

Absolute Difference

Arithmetic Mean	0.178 Gy
Min (LR = 35.0 mm; TG = 15.0 mm)	0.006 Gy
Max (LR = 0.0 mm; TG = 10.0 mm)	1.026 Gy
Median	0.112 Gy

Settings

Passing criteria	Gamma ≤ 1.0
Green	90.0 % to 100.0 %
Yellow	75.0 % to 90.0 %
Red	0.0 % to 75.0 %

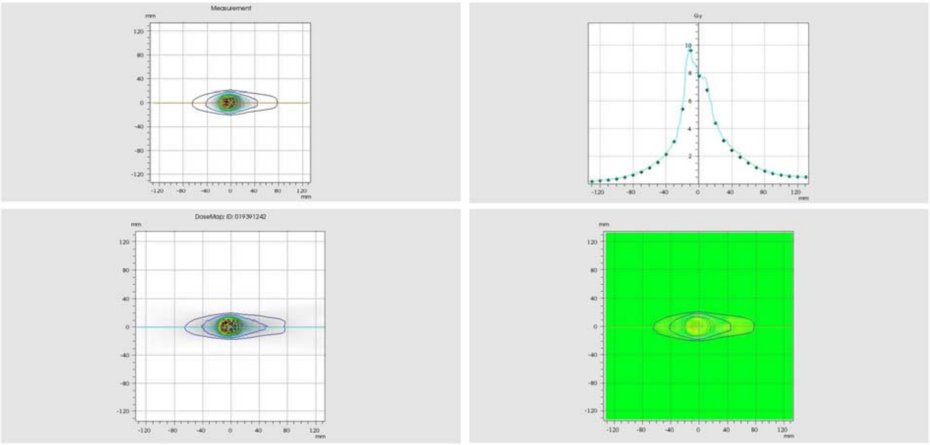

Supplemental Figure 20. Report generated by the MapCheck software comparing the virtual source model Monte Carlo dose to measurements made in a water phantom for the “Phantom 10XFFF-5” test case.

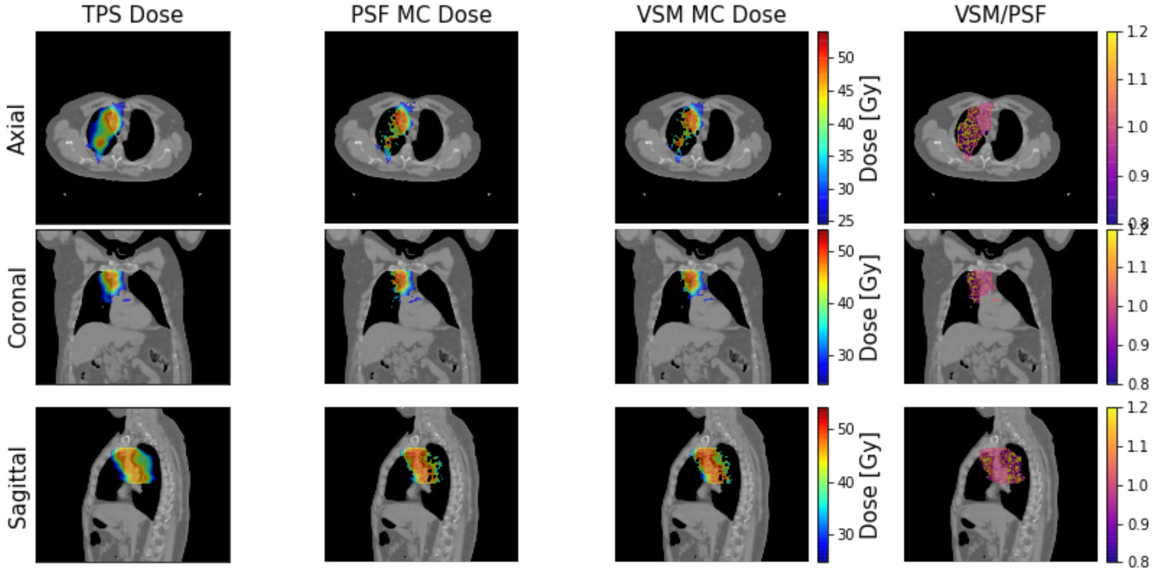

Supplemental Figure 21. Axial, coronal, and sagittal Monte Carlo dose profiles for the "Clinical 6X-1" test case. The first column shows the profiles for the planned (TPS) dose, the second column shows the profiles for the phase-space file (PSF) Monte Carlo dose, the third column shows the profiles for the virtual source model (VSM) Monte Carlo dose, and the fourth column shows the ratio of the VSM to the PSF dose.

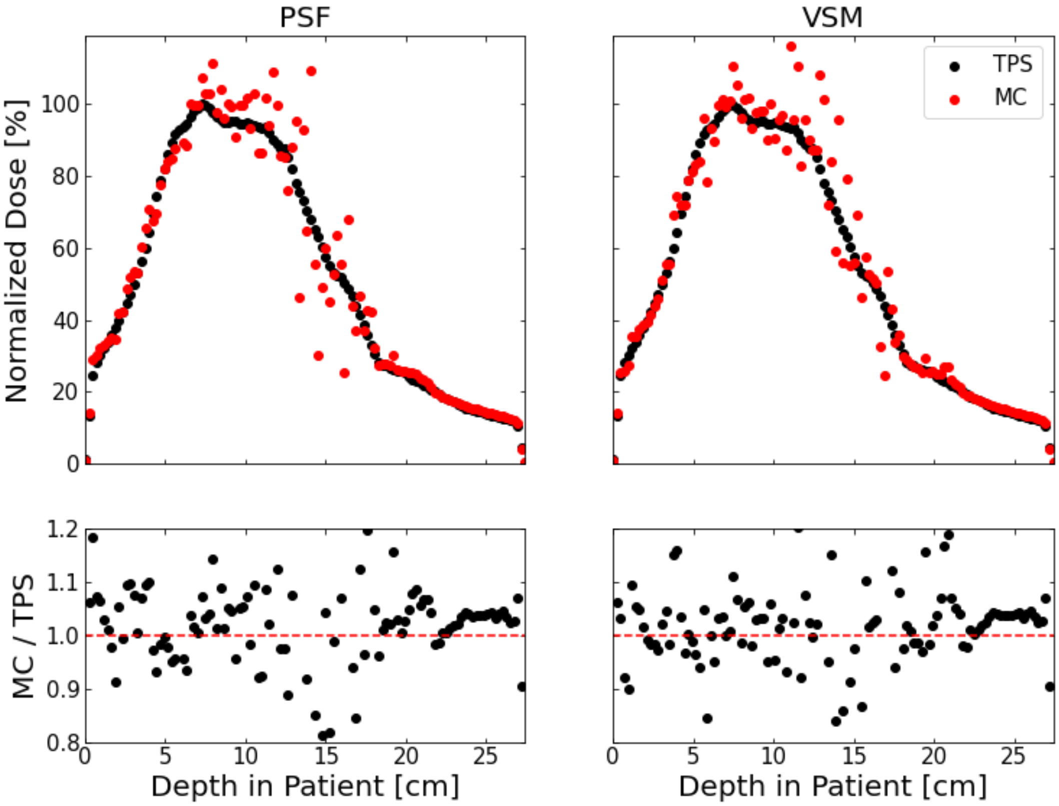

Supplemental Figure 22. Normalized 1D dose profiles for Monte Carlo (MC) doses obtained using a phase-space file (PSF, left) and a virtual source model (VSM, right) for the "Clinical 6X-1" test case. Dose profiles are presented for both the planned dose (black dots) as well as the Monte Carlo doses (red dots).

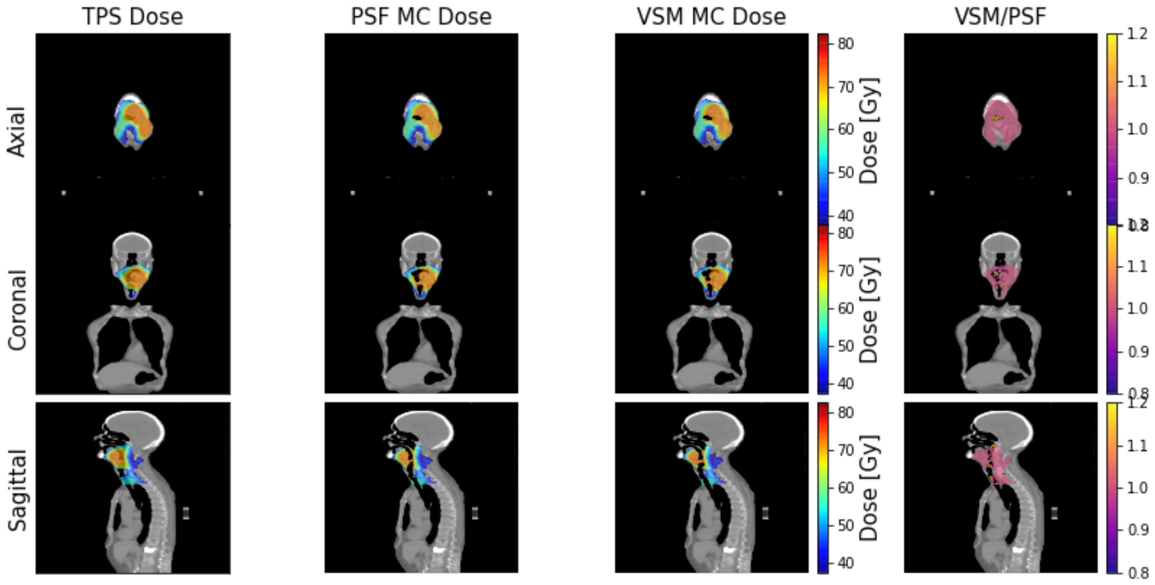

Supplemental Figure 23. Axial, coronal, and sagittal Monte Carlo dose profiles for the “Clinical 6X-2” test case. The first column shows the profiles for the planned (TPS) dose, the second column shows the profiles for the phase-space file (PSF) Monte Carlo dose, the third column shows the profiles for the virtual source model (VSM) Monte Carlo dose, and the fourth column shows the ratio of the VSM to the PSF dose.

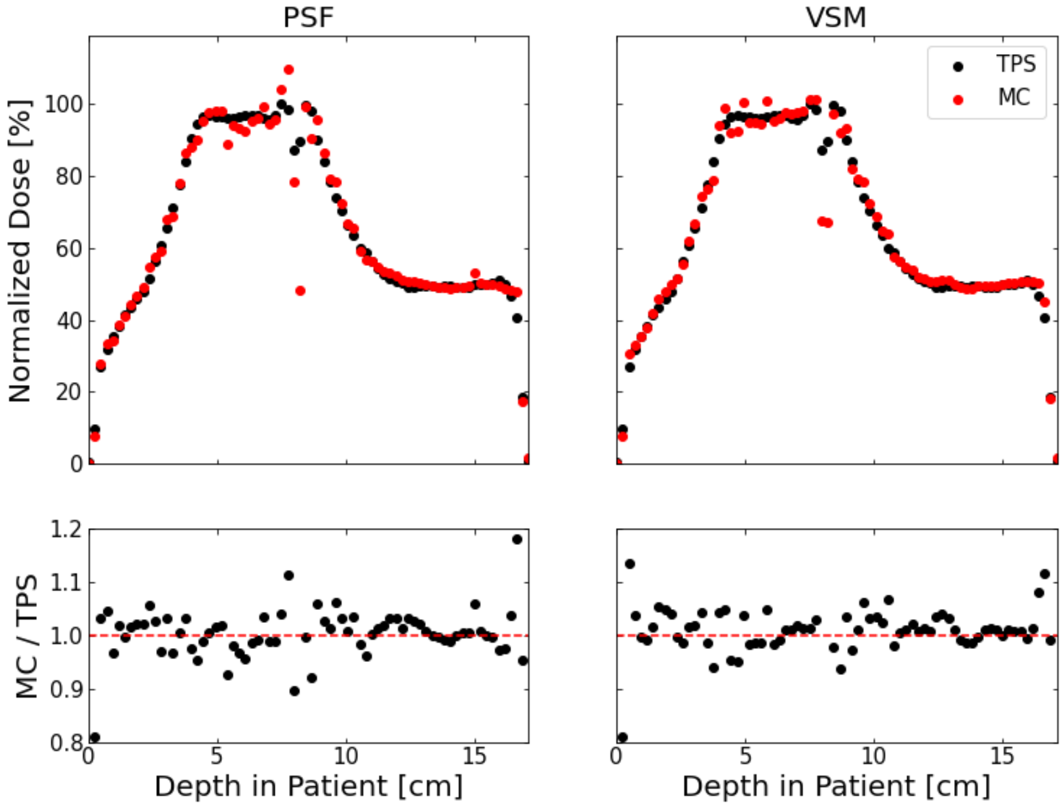

Supplemental Figure 24. Normalized 1D dose profiles for Monte Carlo (MC) doses obtained using a phase-space file (PSF, left) and a virtual source model (VSM, right) for the "Clinical 6X-2" test case. Dose profiles are presented for both the planned dose (black dots) as well as the Monte Carlo doses (red dots).

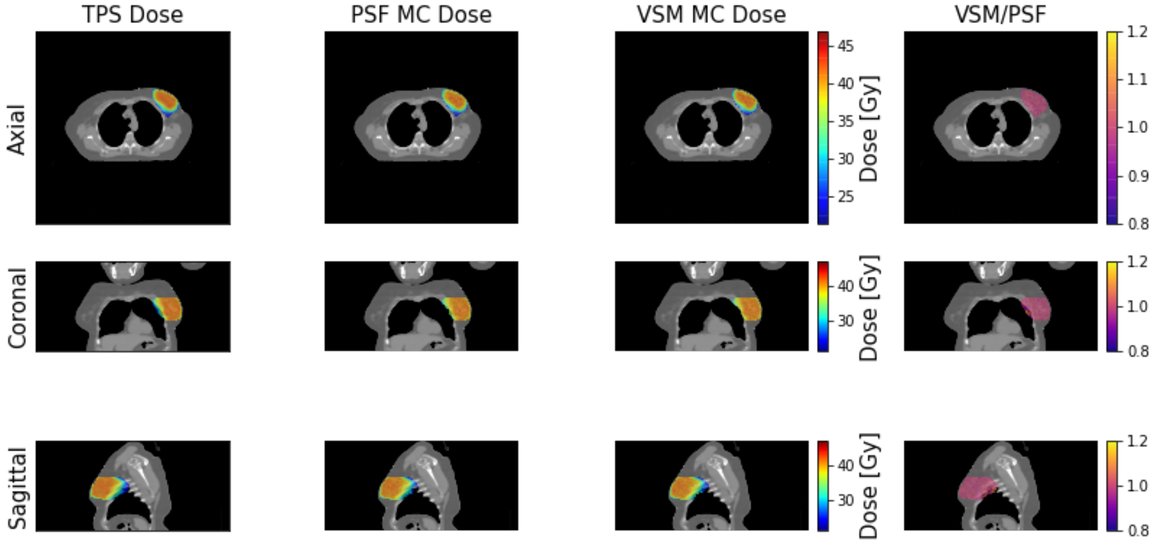

Supplemental Figure 25. Axial, coronal, and sagittal Monte Carlo dose profiles for the "Clinical 6X-3" test case. The first column shows the profiles for the planned (TPS) dose, the second column shows the profiles for the phase-space file (PSF) Monte Carlo dose, the third column shows the profiles for the virtual source model (VSM) Monte Carlo dose, and the fourth column shows the ratio of the VSM to the PSF dose.

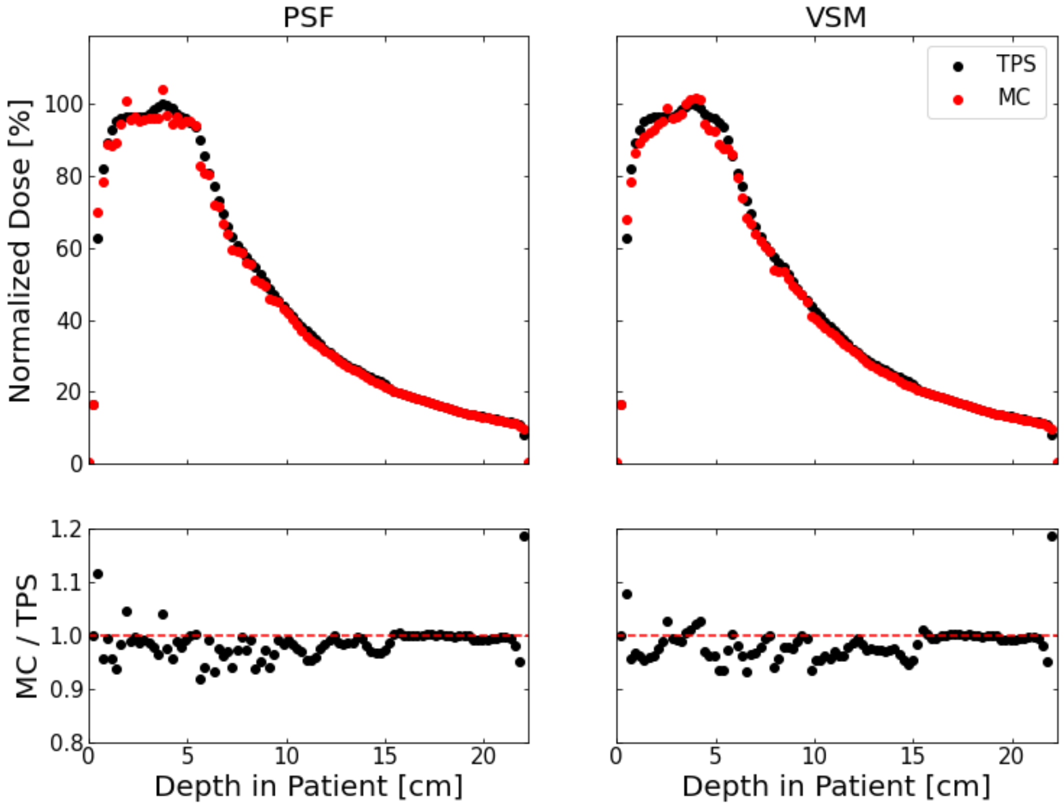

Supplemental Figure 26. Normalized 1D dose profiles for Monte Carlo (MC) doses obtained using a phase-space file (PSF, left) and a virtual source model (VSM, right) for the "Clinical 6X-3" test case. Dose profiles are presented for both the planned dose (black dots) as well as the Monte Carlo doses (red dots).

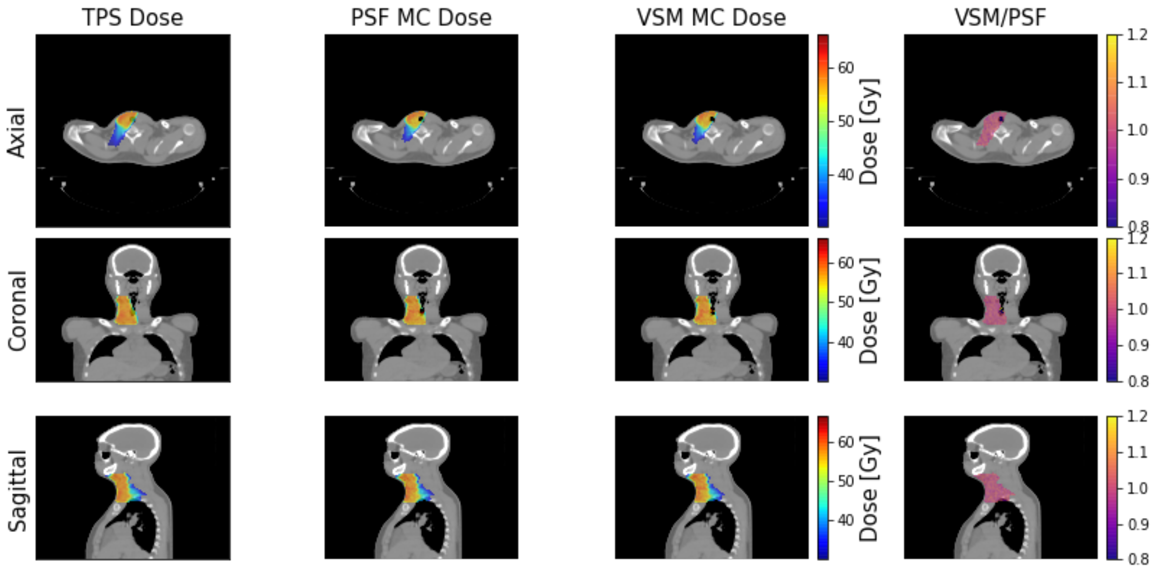

Supplemental Figure 27. Axial, coronal, and sagittal Monte Carlo dose profiles for the “Clinical 6X-4” test case. The first column shows the profiles for the planned (TPS) dose, the second column shows the profiles for the phase-space file (PSF) Monte Carlo dose, the third column shows the profiles for the virtual source model (VSM) Monte Carlo dose, and the fourth column shows the ratio of the VSM to the PSF dose.

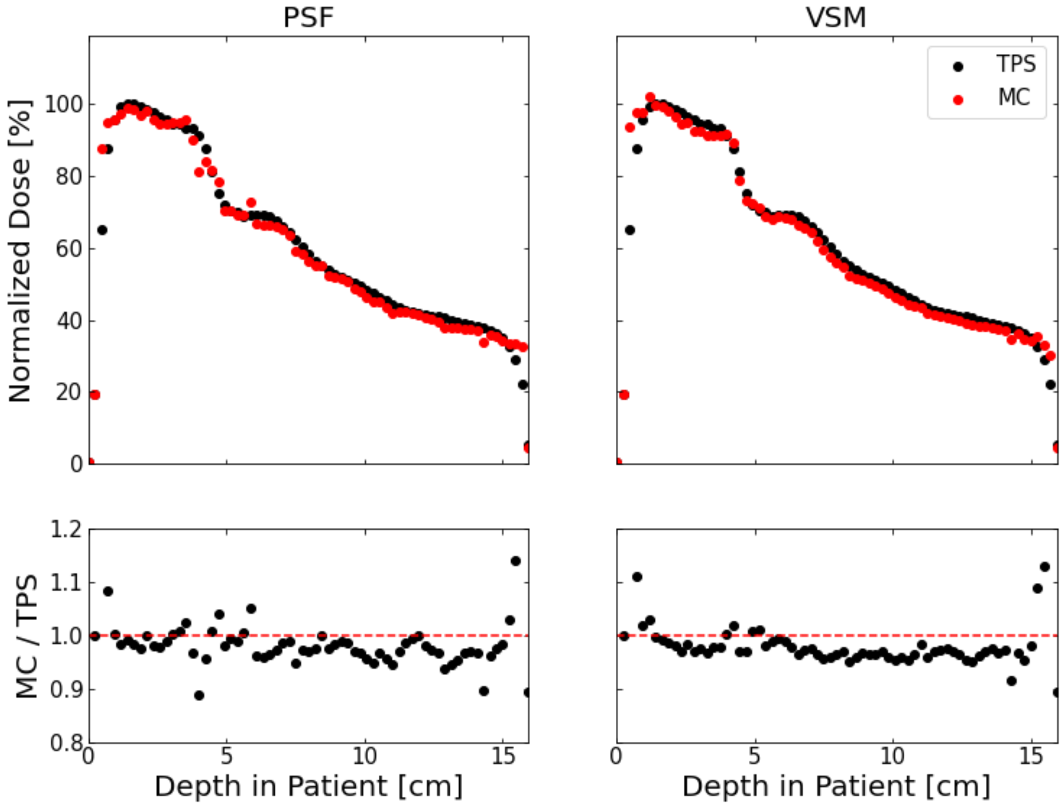

Supplemental Figure 28. Normalized 1D dose profiles for Monte Carlo (MC) doses obtained using a phase-space file (PSF, left) and a virtual source model (VSM, right) for the "Clinical 6X-4" test case. Dose profiles are presented for both the planned dose (black dots) as well as the Monte Carlo doses (red dots).

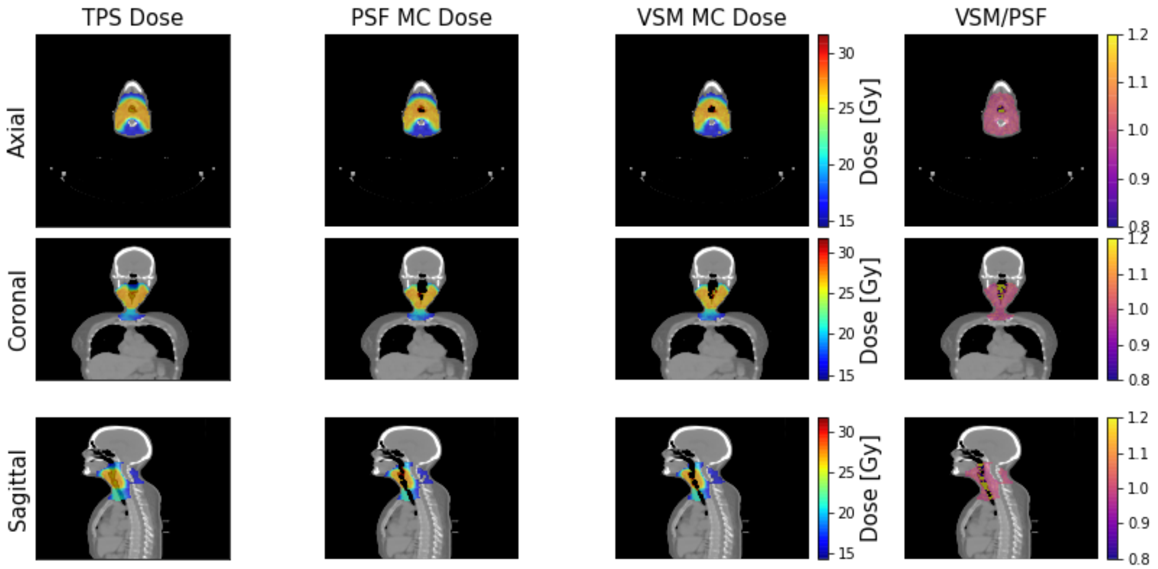

Supplemental Figure 29. Axial, coronal, and sagittal Monte Carlo dose profiles for the “Clinical 6X-5” test case. The first column shows the profiles for the planned (TPS) dose, the second column shows the profiles for the phase-space file (PSF) Monte Carlo dose, the third column shows the profiles for the virtual source model (VSM) Monte Carlo dose, and the fourth column shows the ratio of the VSM to the PSF dose.

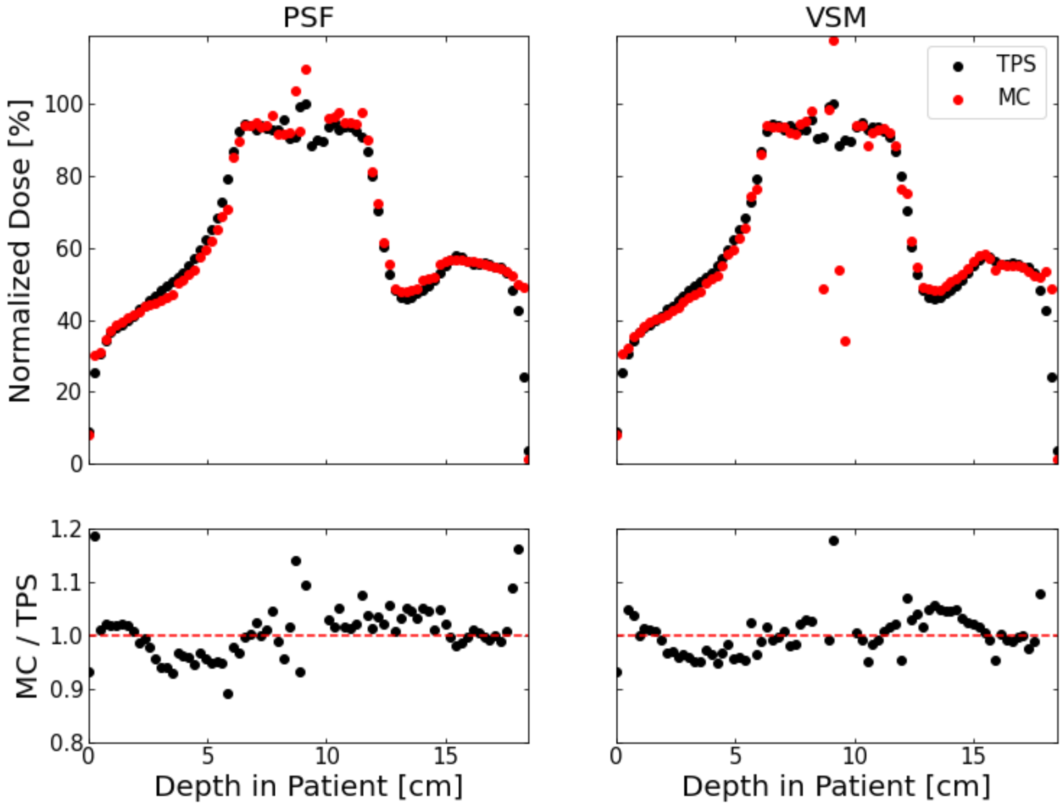

Supplemental Figure 30. Normalized 1D dose profiles for Monte Carlo (MC) doses obtained using a phase-space file (PSF, left) and a virtual source model (VSM, right) for the "Clinical 6X-5" test case. Dose profiles are presented for both the planned dose (black dots) as well as the Monte Carlo doses (red dots).

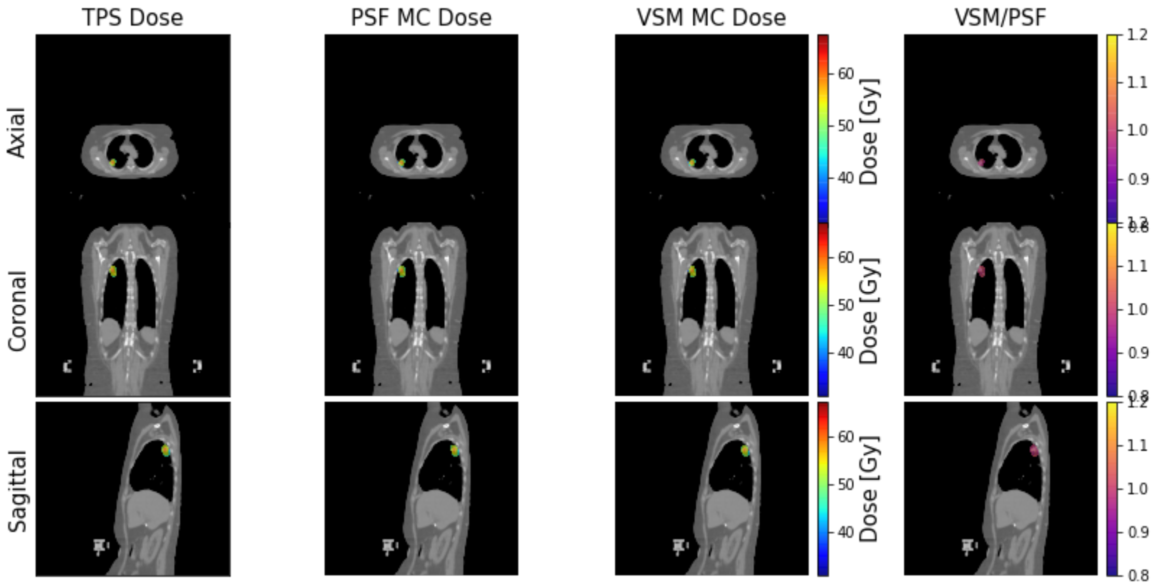

Supplemental Figure 41. Axial, coronal, and sagittal Monte Carlo dose profiles for the "Clinical 6XFFF-1" test case. The first column shows the profiles for the planned (TPS) dose, the second column shows the profiles for the phase-space file (PSF) Monte Carlo dose, the third column shows the profiles for the virtual source model (VSM) Monte Carlo dose, and the fourth column shows the ratio of the VSM to the PSF dose.

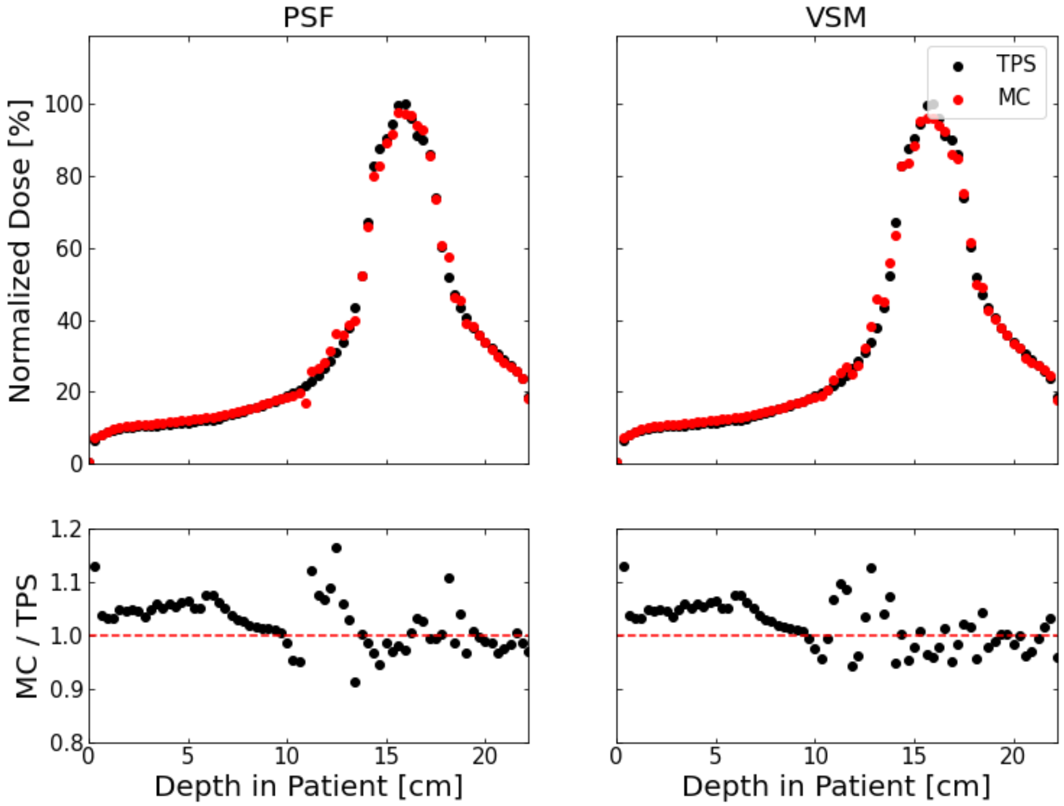

Supplemental Figure 42. Normalized 1D dose profiles for Monte Carlo (MC) doses obtained using a phase-space file (PSF, left) and a virtual source model (VSM, right) for the "Clinical 6XFFF-1" test case. Dose profiles are presented for both the planned dose (black dots) as well as the Monte Carlo doses (red dots).

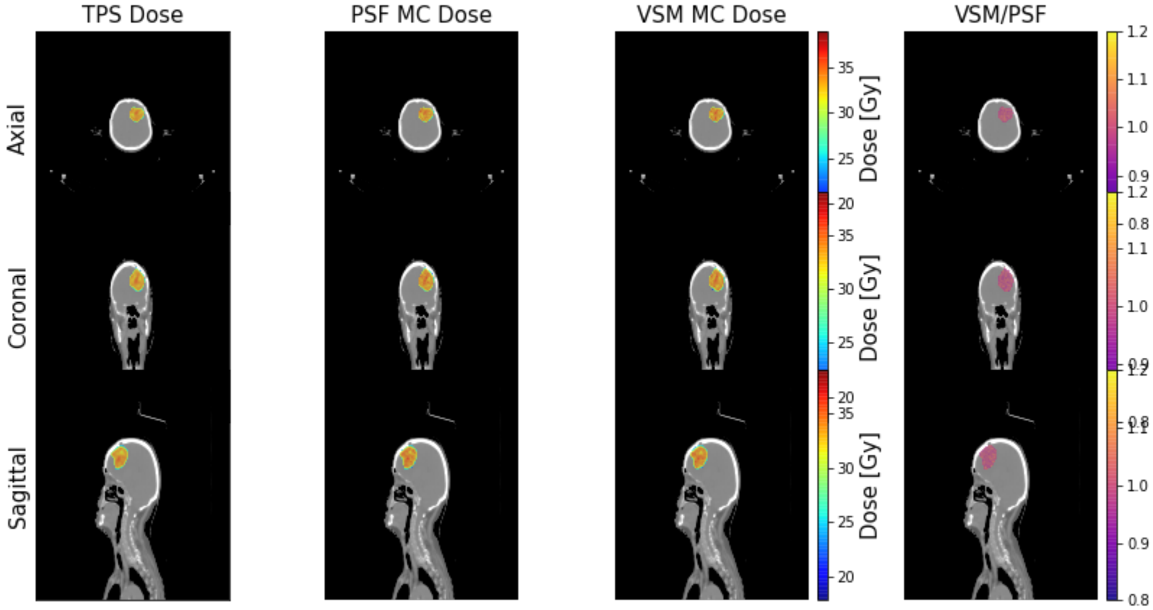

Supplemental Figure 43. Axial, coronal, and sagittal Monte Carlo dose profiles for the "Clinical 6XFFF-2" test case. The first column shows the profiles for the planned (TPS) dose, the second column shows the profiles for the phase-space file (PSF) Monte Carlo dose, the third column shows the profiles for the virtual source model (VSM) Monte Carlo dose, and the fourth column shows the ratio of the VSM to the PSF dose.

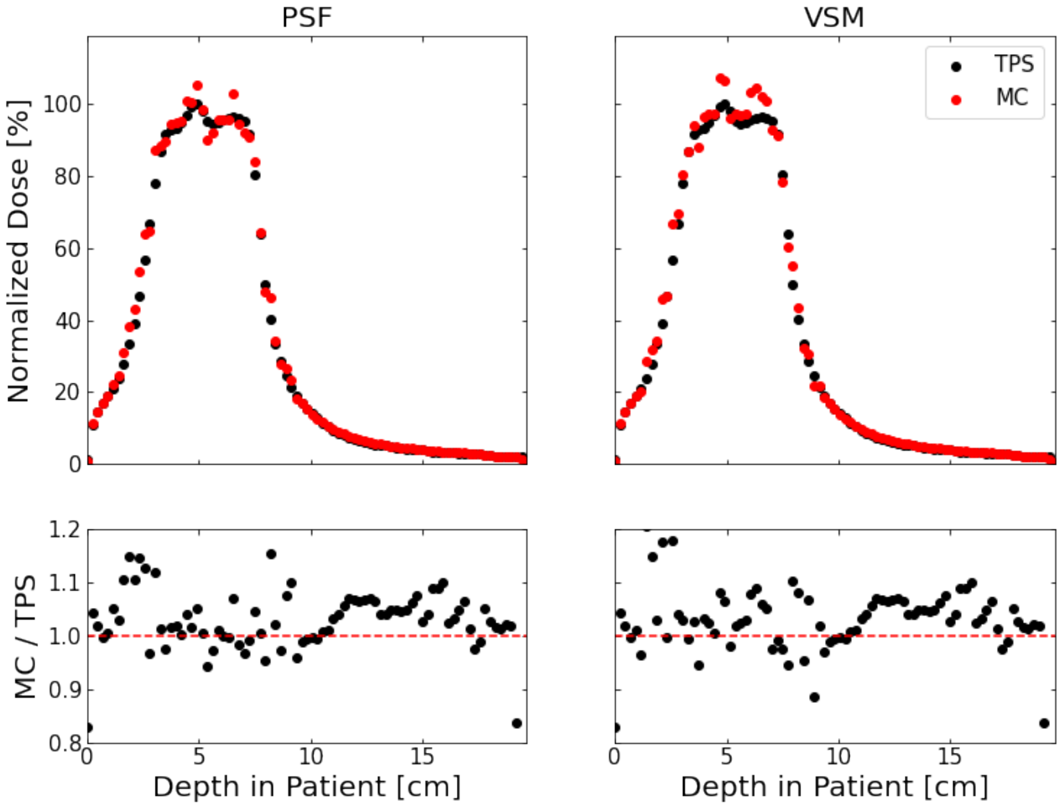

Supplemental Figure 44. Normalized 1D dose profiles for Monte Carlo (MC) doses obtained using a phase-space file (PSF, left) and a virtual source model (VSM, right) for the "Clinical 6XFFF-2" test case. Dose profiles are presented for both the planned dose (black dots) as well as the Monte Carlo doses (red dots).

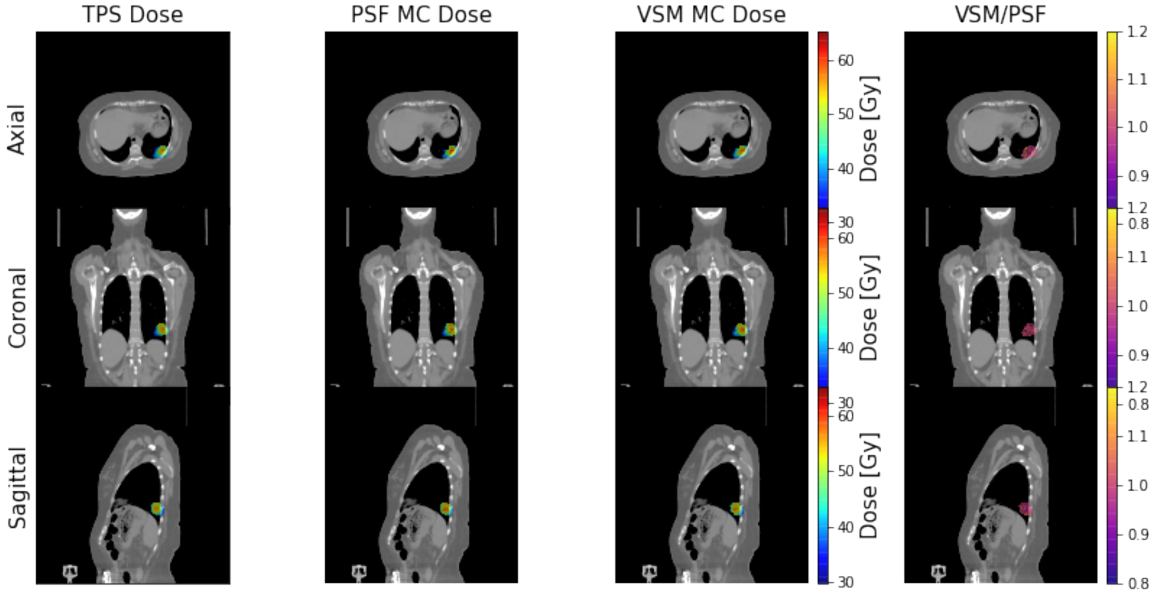

Supplemental Figure 45. Axial, coronal, and sagittal Monte Carlo dose profiles for the “Clinical 6XFFF-3” test case. The first column shows the profiles for the planned (TPS) dose, the second column shows the profiles for the phase-space file (PSF) Monte Carlo dose, the third column shows the profiles for the virtual source model (VSM) Monte Carlo dose, and the fourth column shows the ratio of the VSM to the PSF dose.

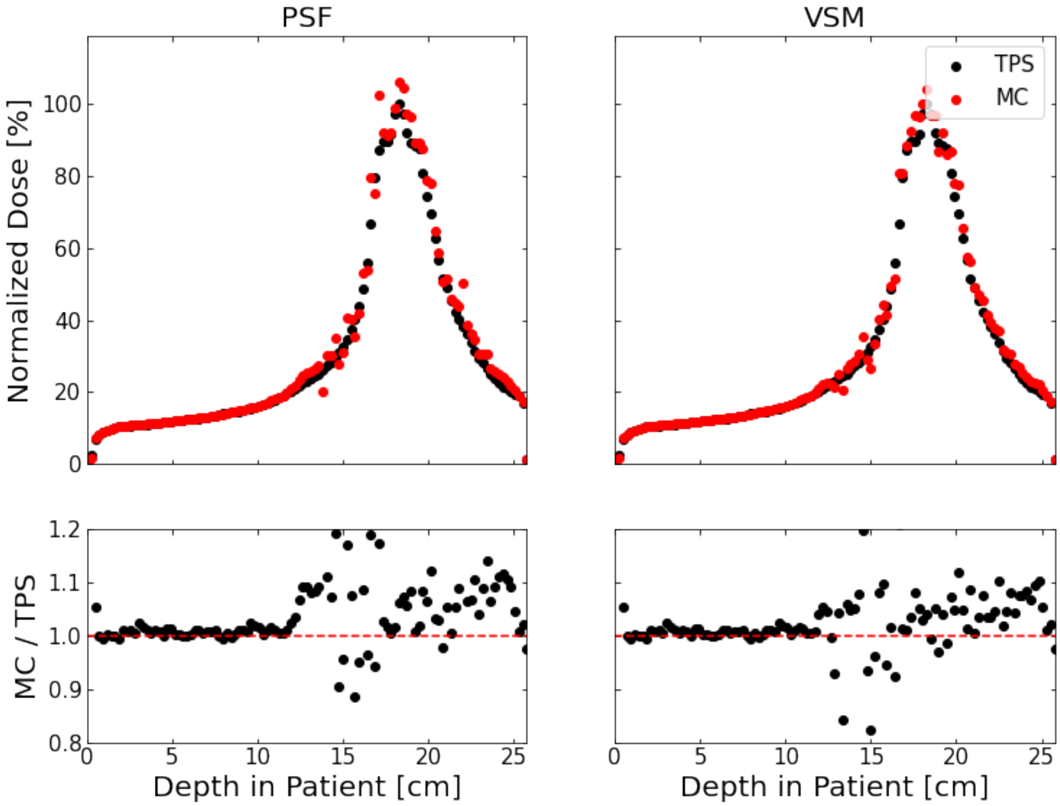

Supplemental Figure 46. Normalized 1D dose profiles for Monte Carlo (MC) doses obtained using a phase-space file (PSF, left) and a virtual source model (VSM, right) for the "Clinical 6XFFF-3" test case. Dose profiles are presented for both the planned dose (black dots) as well as the Monte Carlo doses (red dots).

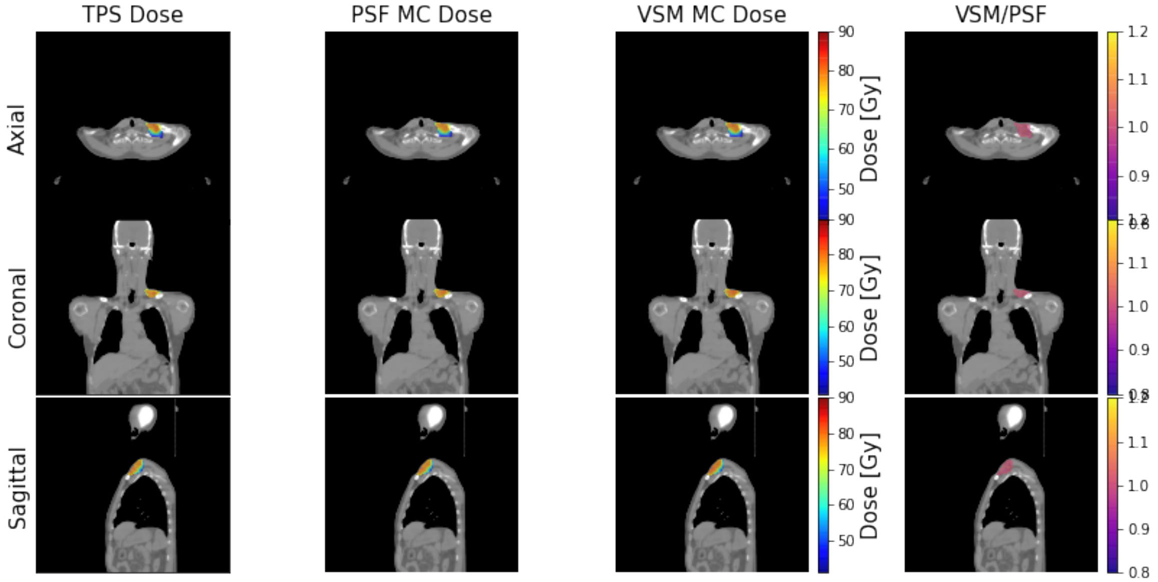

Supplemental Figure 47. Axial, coronal, and sagittal Monte Carlo dose profiles for the "Clinical 6XFFF-4" test case. The first column shows the profiles for the planned (TPS) dose, the second column shows the profiles for the phase-space file (PSF) Monte Carlo dose, the third column shows the profiles for the virtual source model (VSM) Monte Carlo dose, and the fourth column shows the ratio of the VSM to the PSF dose.

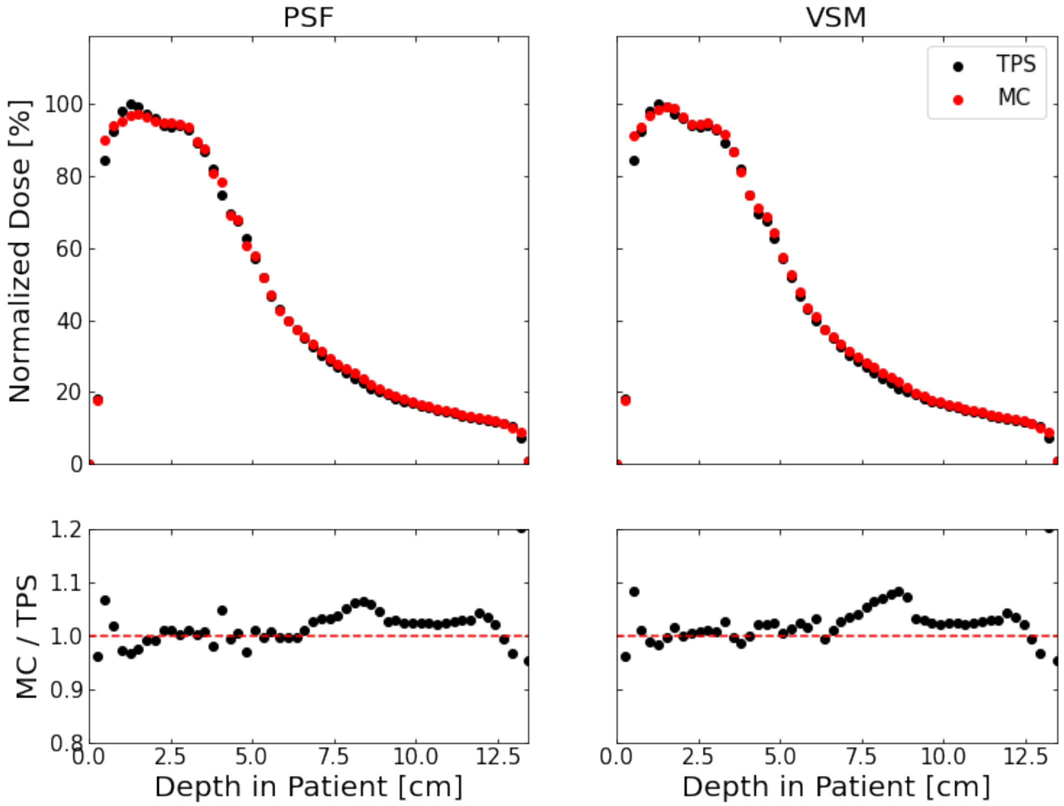

Supplemental Figure 48. Normalized 1D dose profiles for Monte Carlo (MC) doses obtained using a phase-space file (PSF, left) and a virtual source model (VSM, right) for the "Clinical 6XFFF-4" test case. Dose profiles are presented for both the planned dose (black dots) as well as the Monte Carlo doses (red dots).

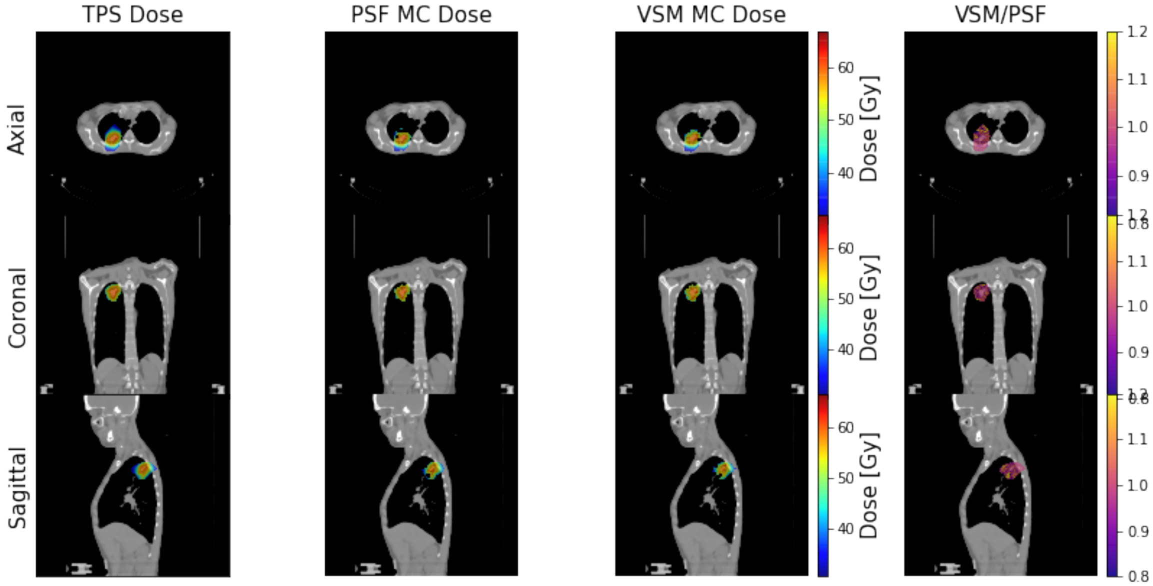

Supplemental Figure 49. Axial, coronal, and sagittal Monte Carlo dose profiles for the "Clinical 6XFFF-5" test case. The first column shows the profiles for the planned (TPS) dose, the second column shows the profiles for the phase-space file (PSF) Monte Carlo dose, the third column shows the profiles for the virtual source model (VSM) Monte Carlo dose, and the fourth column shows the ratio of the VSM to the PSF dose.

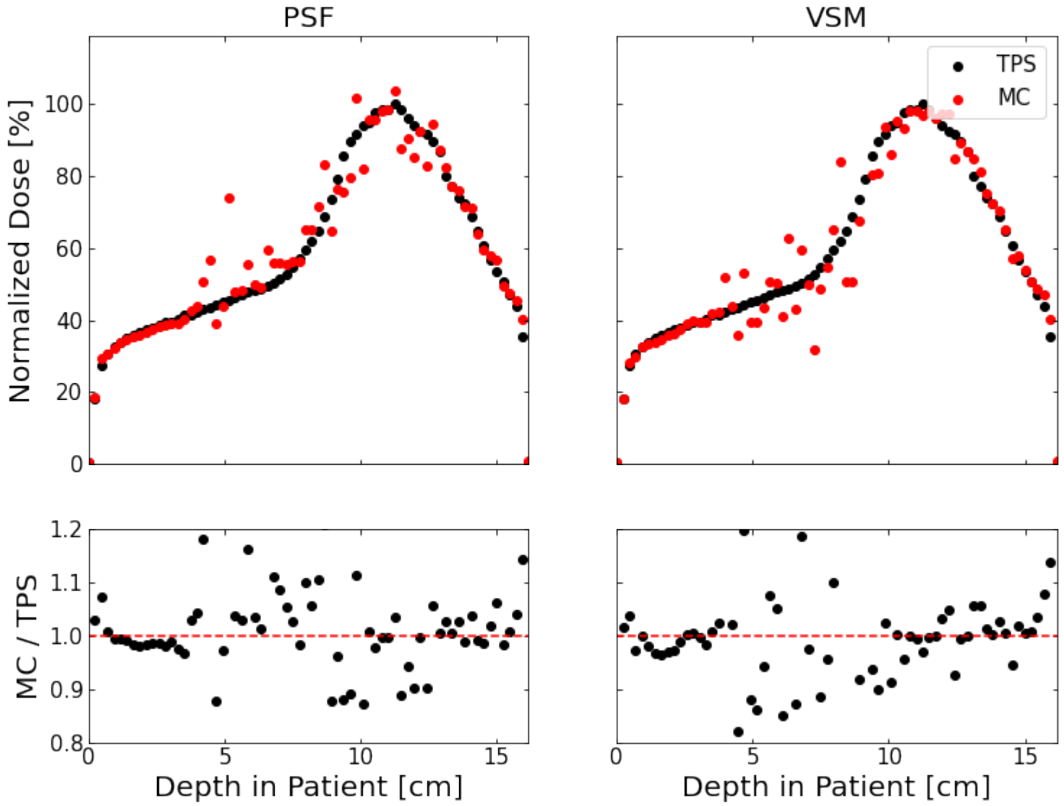

Supplemental Figure 50. Normalized 1D dose profiles for Monte Carlo (MC) doses obtained using a phase-space file (PSF, left) and a virtual source model (VSM, right) for the "Clinical 6XFFF-5" test case. Dose profiles are presented for both the planned dose (black dots) as well as the Monte Carlo doses (red dots).

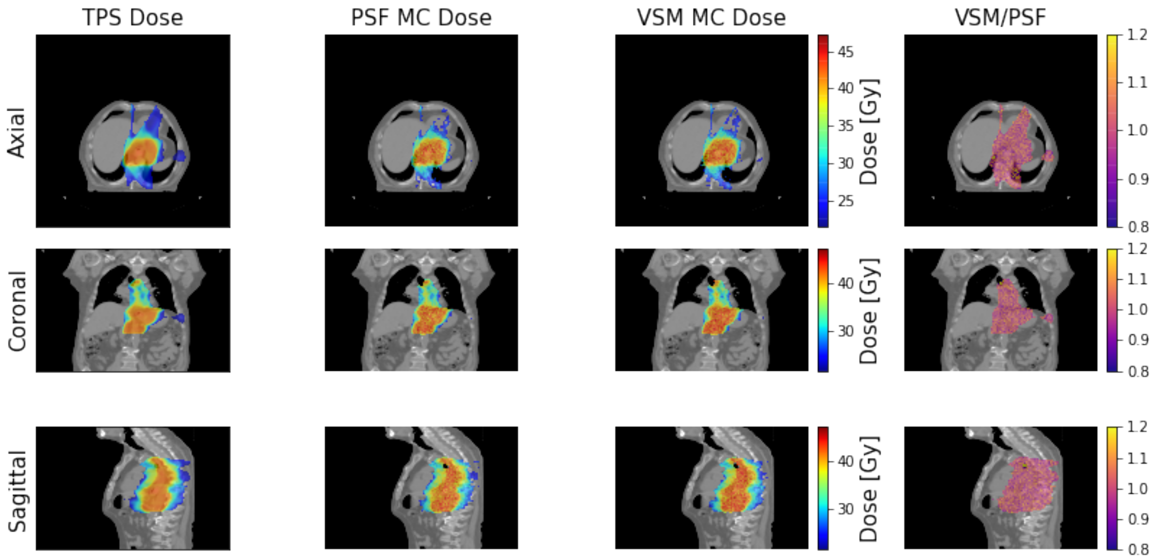

Supplemental Figure 51. Axial, coronal, and sagittal Monte Carlo dose profiles for the "Clinical 10X-1" test case. The first column shows the profiles for the planned (TPS) dose, the second column shows the profiles for the phase-space file (PSF) Monte Carlo dose, the third column shows the profiles for the virtual source model (VSM) Monte Carlo dose, and the fourth column shows the ratio of the VSM to the PSF dose.

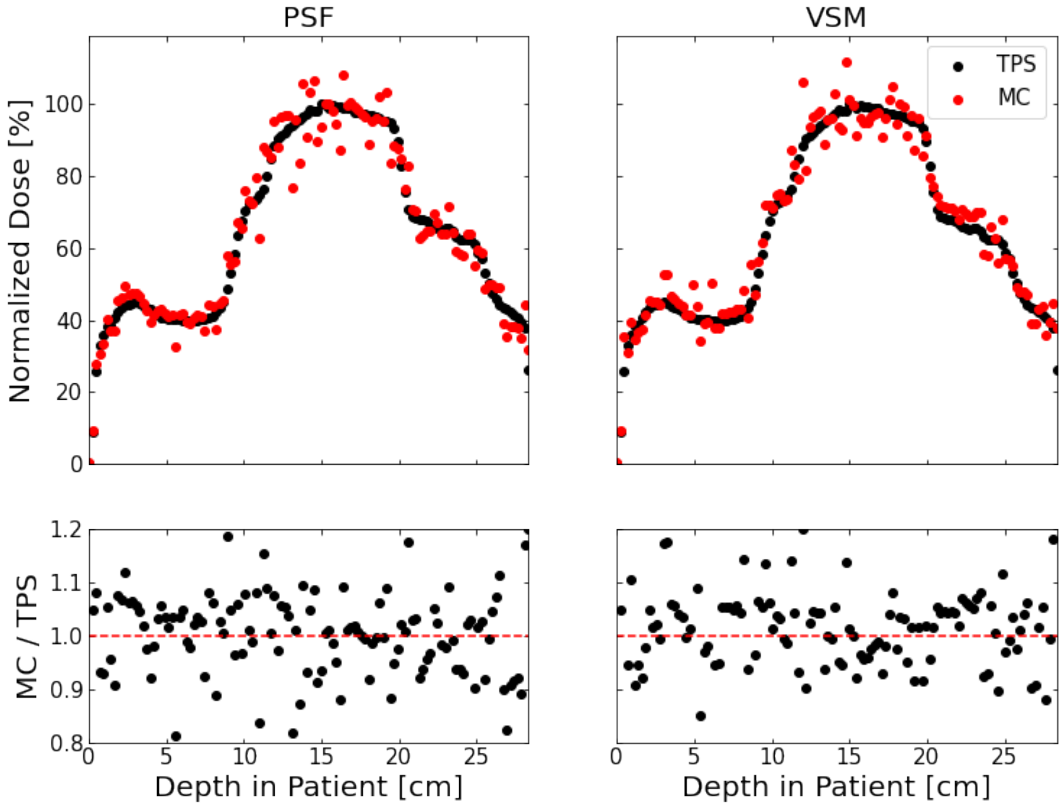

Supplemental Figure 52. Normalized 1D dose profiles for Monte Carlo (MC) doses obtained using a phase-space file (PSF, left) and a virtual source model (VSM, right) for the "Clinical 10X-1" test case. Dose profiles are presented for both the planned dose (black dots) as well as the Monte Carlo doses (red dots).

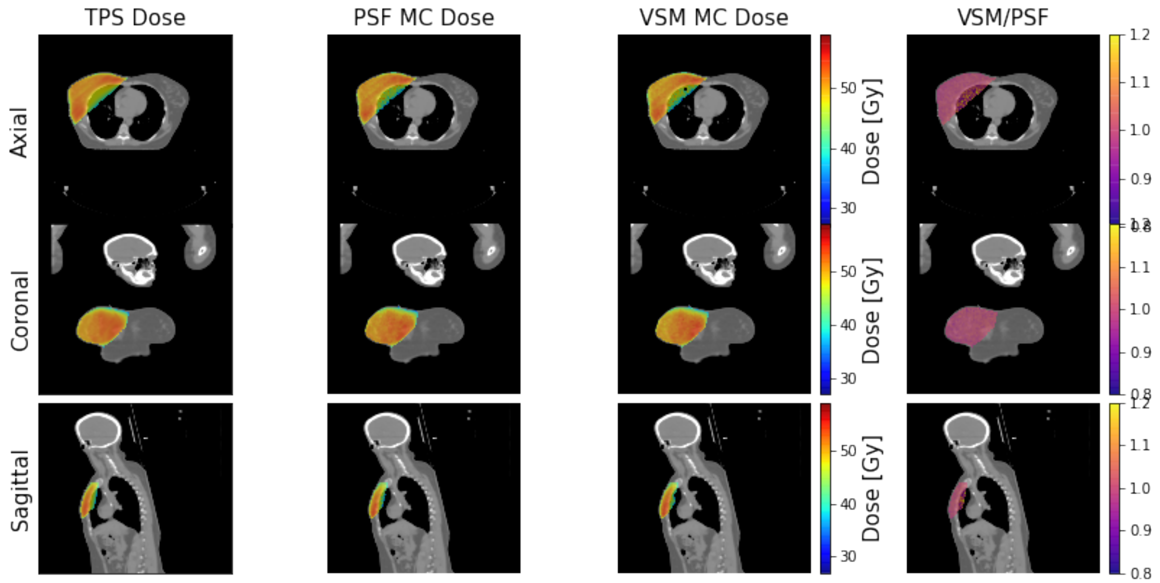

Supplemental Figure 53. Axial, coronal, and sagittal Monte Carlo dose profiles for the "Clinical 10X-2" test case. The first column shows the profiles for the planned (TPS) dose, the second column shows the profiles for the phase-space file (PSF) Monte Carlo dose, the third column shows the profiles for the virtual source model (VSM) Monte Carlo dose, and the fourth column shows the ratio of the VSM to the PSF dose.

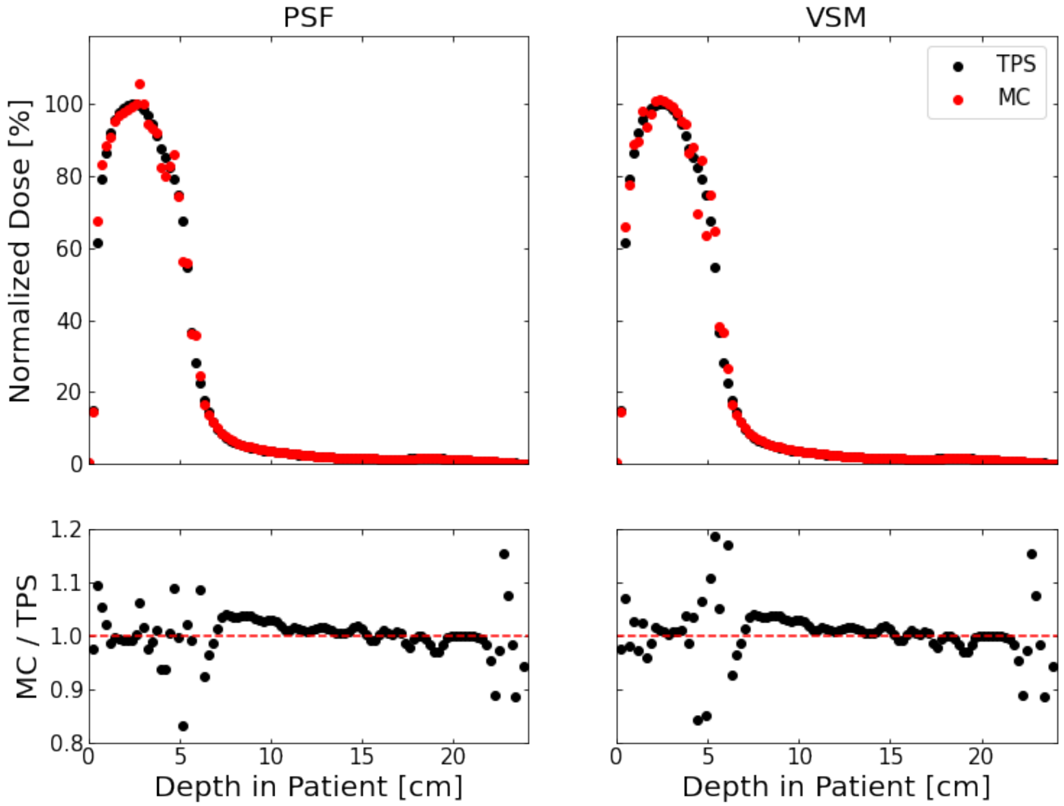

Supplemental Figure 54. Normalized 1D dose profiles for Monte Carlo (MC) doses obtained using a phase-space file (PSF, left) and a virtual source model (VSM, right) for the "Clinical 10X-2" test case. Dose profiles are presented for both the planned dose (black dots) as well as the Monte Carlo doses (red dots).

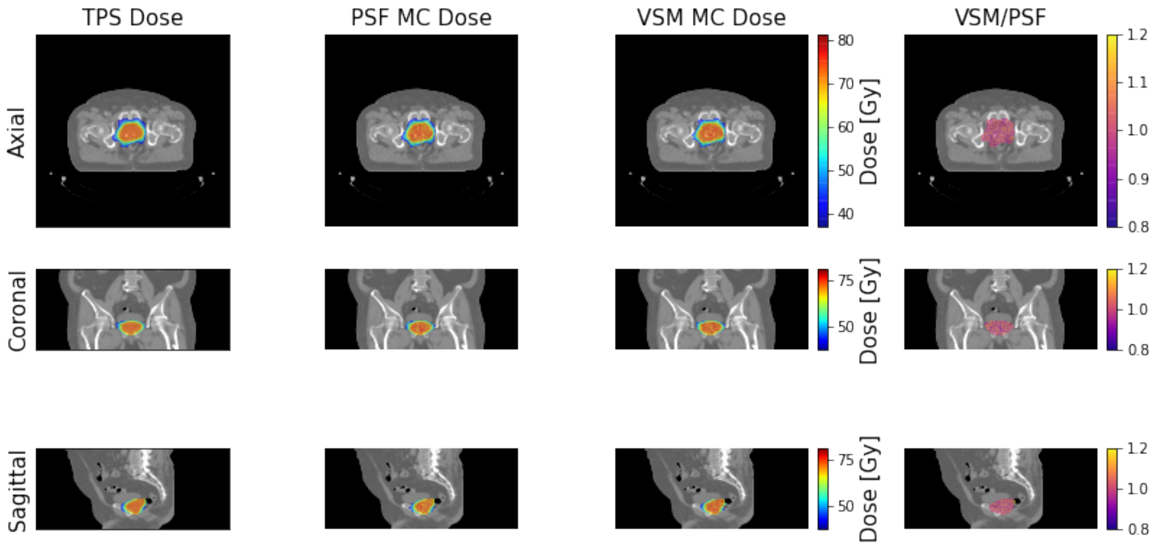

Supplemental Figure 55. Axial, coronal, and sagittal Monte Carlo dose profiles for the "Clinical 10X-3" test case. The first column shows the profiles for the planned (TPS) dose, the second column shows the profiles for the phase-space file (PSF) Monte Carlo dose, the third column shows the profiles for the virtual source model (VSM) Monte Carlo dose, and the fourth column shows the ratio of the VSM to the PSF dose.

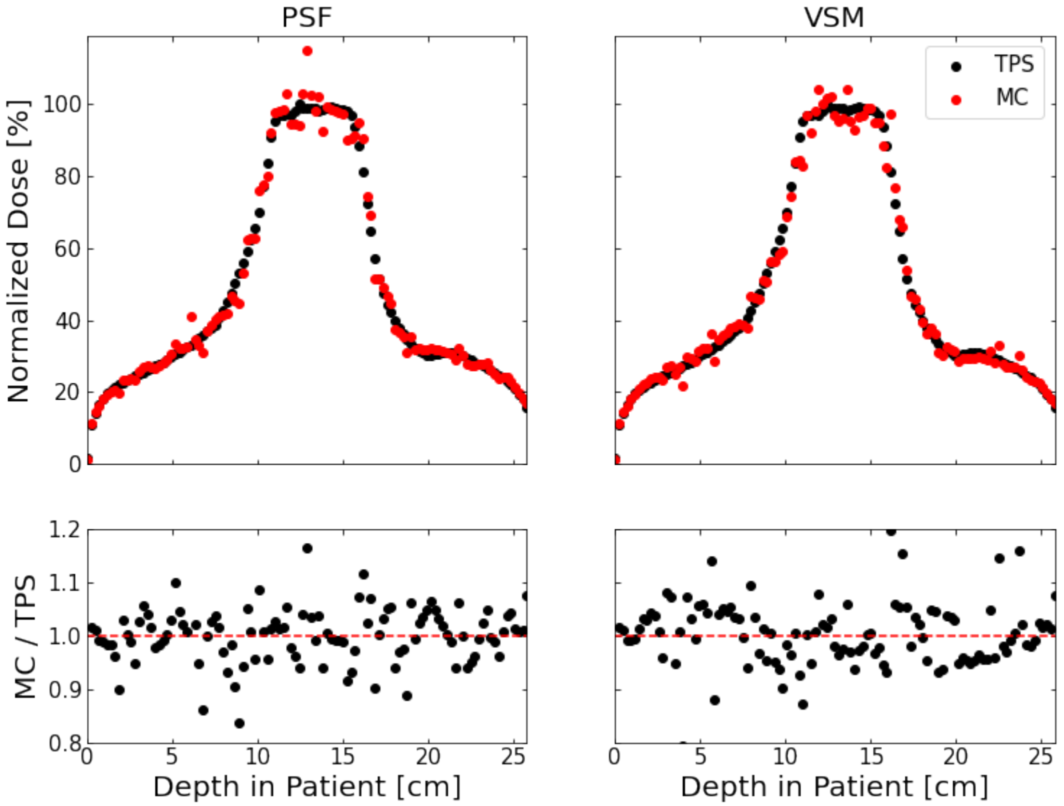

Supplemental Figure 56. Normalized 1D dose profiles for Monte Carlo (MC) doses obtained using a phase-space file (PSF, left) and a virtual source model (VSM, right) for the "Clinical 10X-3" test case. Dose profiles are presented for both the planned dose (black dots) as well as the Monte Carlo doses (red dots).

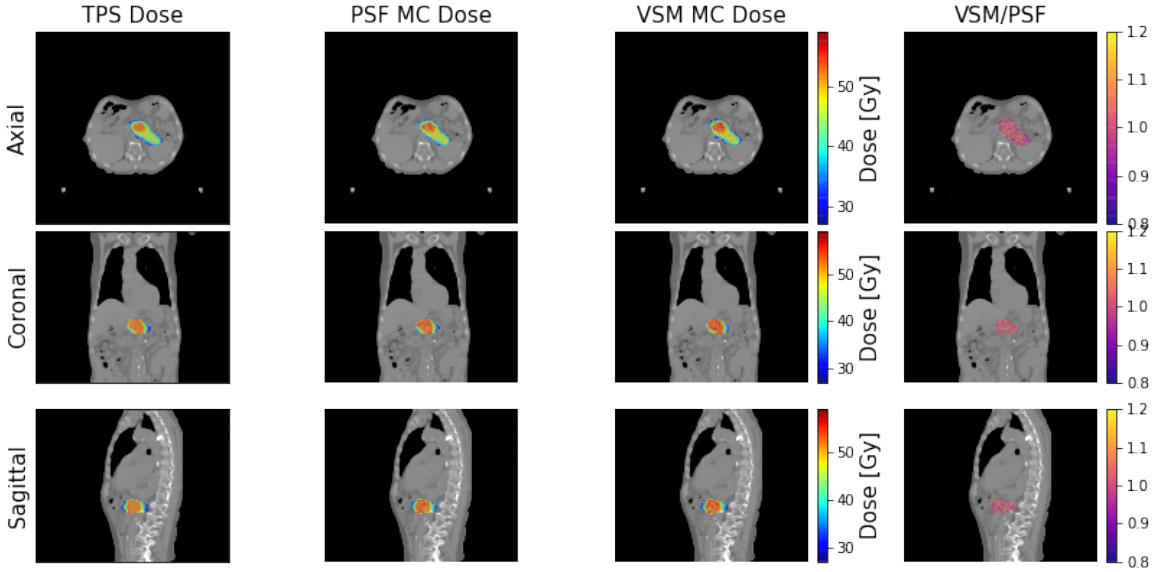

Supplemental Figure 57. Axial, coronal, and sagittal Monte Carlo dose profiles for the "Clinical 10X-4" test case. The first column shows the profiles for the planned (TPS) dose, the second column shows the profiles for the phase-space file (PSF) Monte Carlo dose, the third column shows the profiles for the virtual source model (VSM) Monte Carlo dose, and the fourth column shows the ratio of the VSM to the PSF dose.

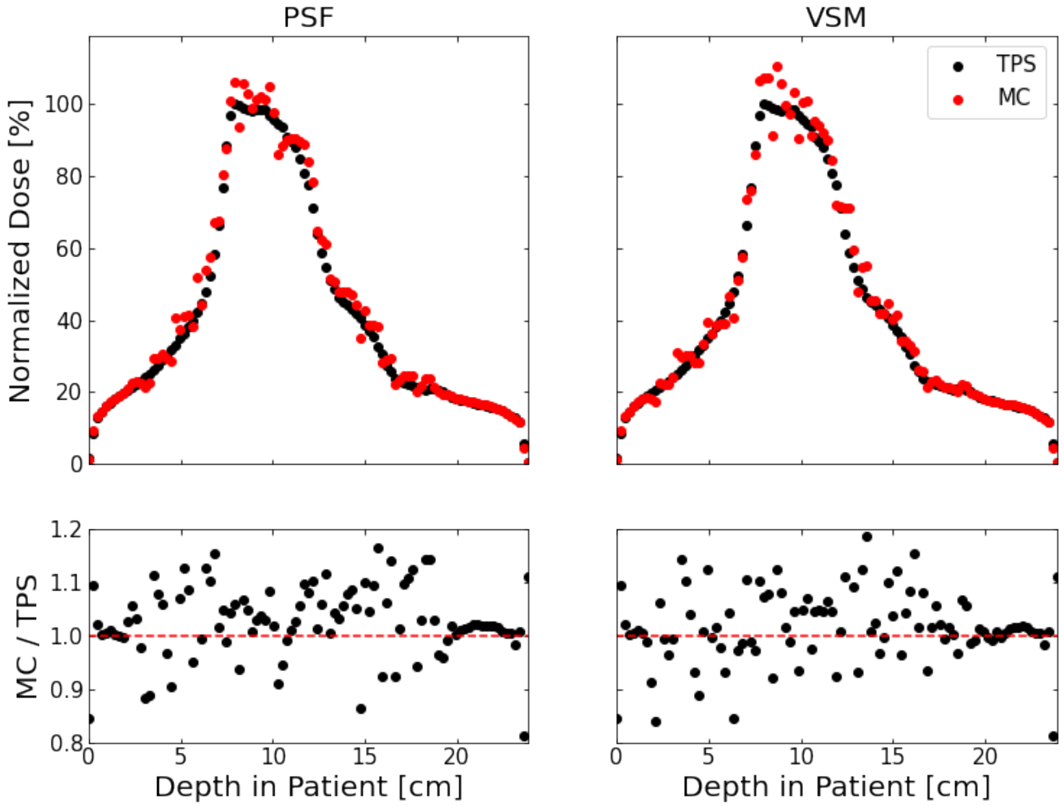

Supplemental Figure 58. Normalized 1D dose profiles for Monte Carlo (MC) doses obtained using a phase-space file (PSF, left) and a virtual source model (VSM, right) for the "Clinical 10X-4" test case. Dose profiles are presented for both the planned dose (black dots) as well as the Monte Carlo doses (red dots).

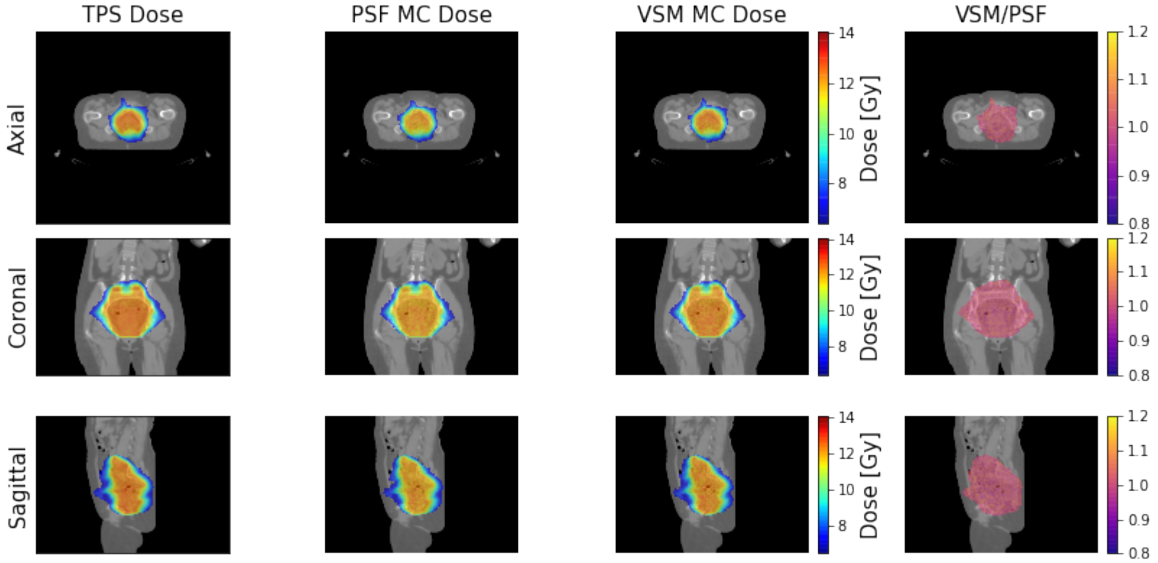

Supplemental Figure 59. Axial, coronal, and sagittal Monte Carlo dose profiles for the "Clinical 10X-5" test case. The first column shows the profiles for the planned (TPS) dose, the second column shows the profiles for the phase-space file (PSF) Monte Carlo dose, the third column shows the profiles for the virtual source model (VSM) Monte Carlo dose, and the fourth column shows the ratio of the VSM to the PSF dose.

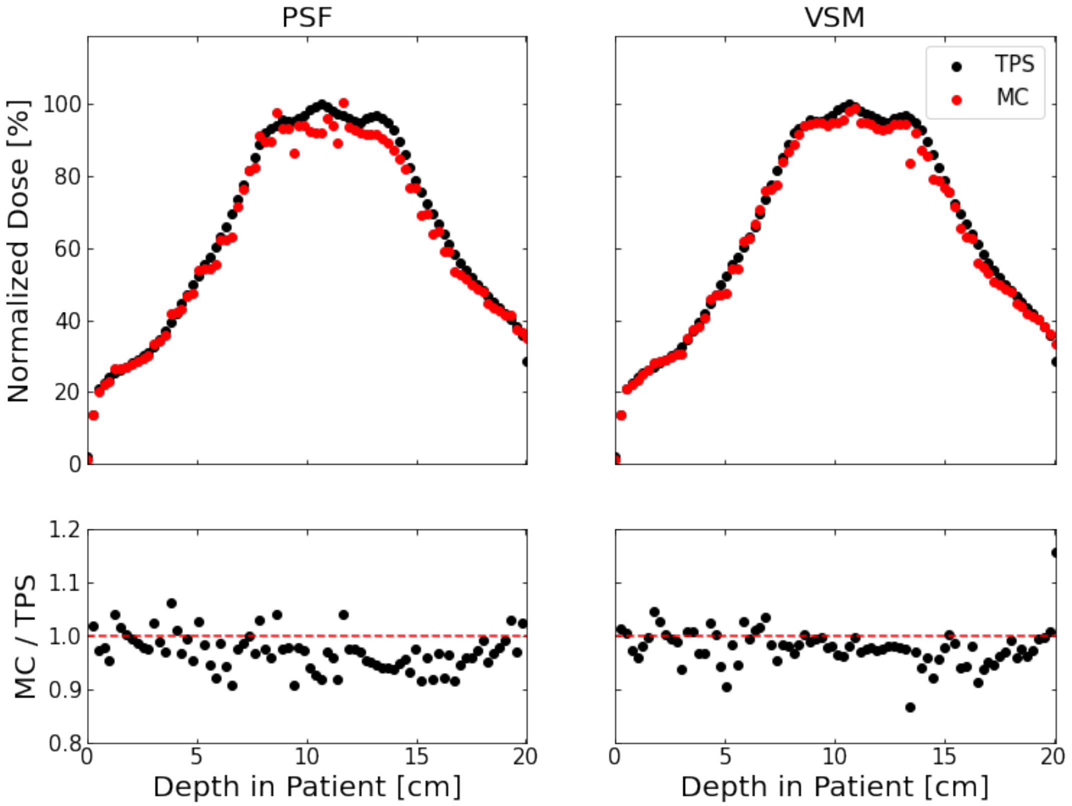

Supplemental Figure 60. Normalized 1D dose profiles for Monte Carlo (MC) doses obtained using a phase-space file (PSF, left) and a virtual source model (VSM, right) for the "Clinical 10X-5" test case. Dose profiles are presented for both the planned dose (black dots) as well as the Monte Carlo doses (red dots).

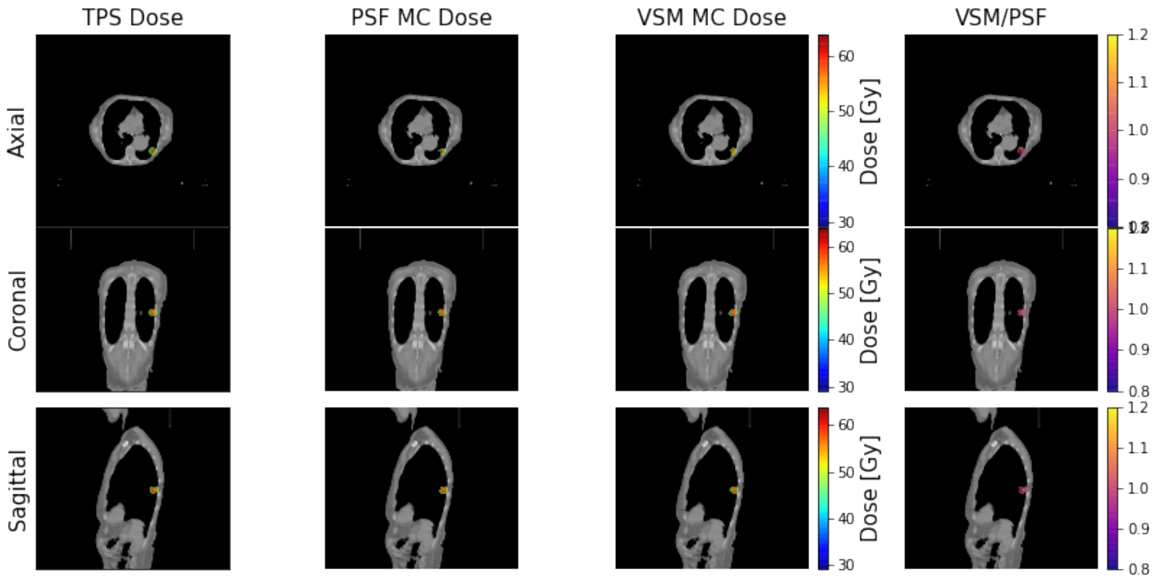

Supplemental Figure 61. Axial, coronal, and sagittal Monte Carlo dose profiles for the "Clinical 10XFFF-1" test case. The first column shows the profiles for the planned (TPS) dose, the second column shows the profiles for the phase-space file (PSF) Monte Carlo dose, the third column shows the profiles for the virtual source model (VSM) Monte Carlo dose, and the fourth column shows the ratio of the VSM to the PSF dose.

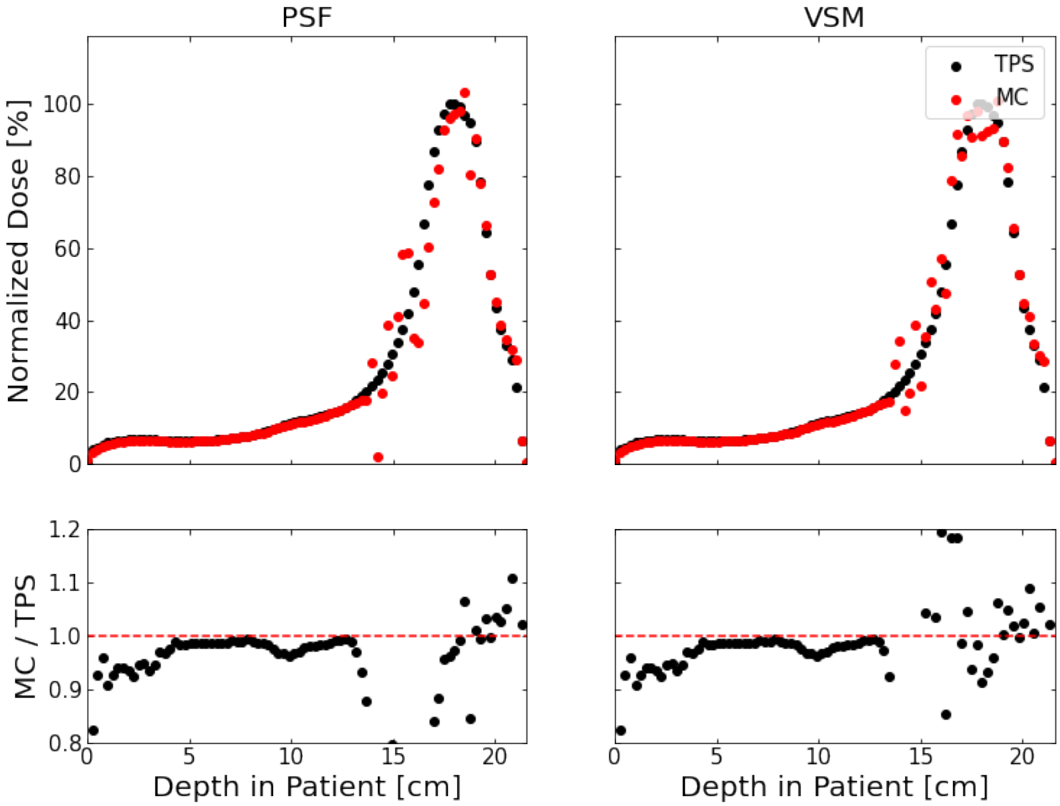

Supplemental Figure 62. Normalized 1D dose profiles for Monte Carlo (MC) doses obtained using a phase-space file (PSF, left) and a virtual source model (VSM, right) for the "Clinical 10XFFF-1" test case. Dose profiles are presented for both the planned dose (black dots) as well as the Monte Carlo doses (red dots).

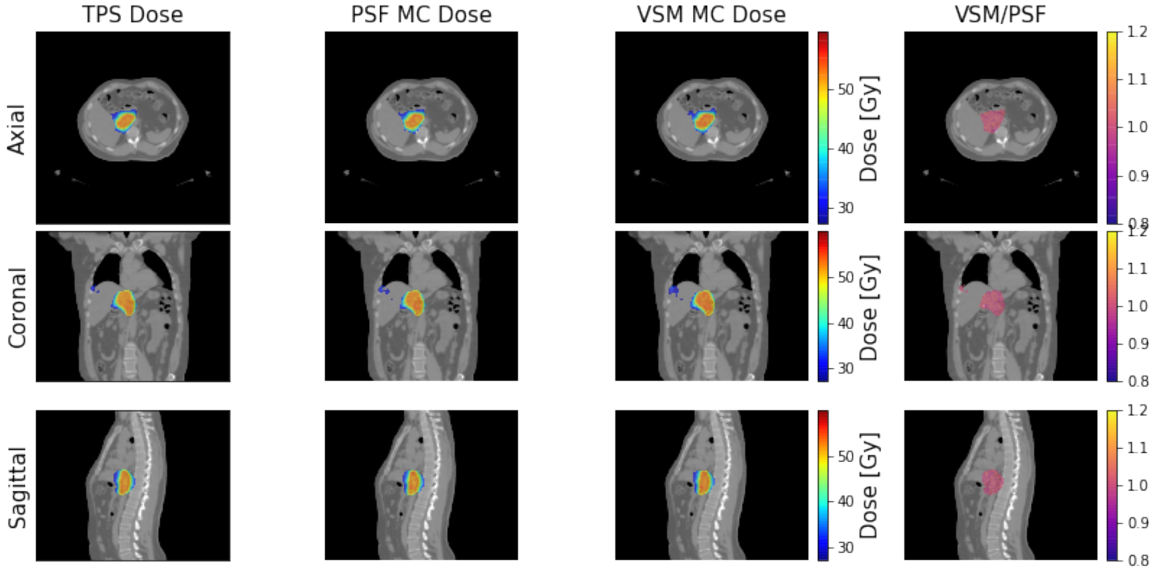

Supplemental Figure 63. Axial, coronal, and sagittal Monte Carlo dose profiles for the "Clinical 10XFFF-2" test case. The first column shows the profiles for the planned (TPS) dose, the second column shows the profiles for the phase-space file (PSF) Monte Carlo dose, the third column shows the profiles for the virtual source model (VSM) Monte Carlo dose, and the fourth column shows the ratio of the VSM to the PSF dose.

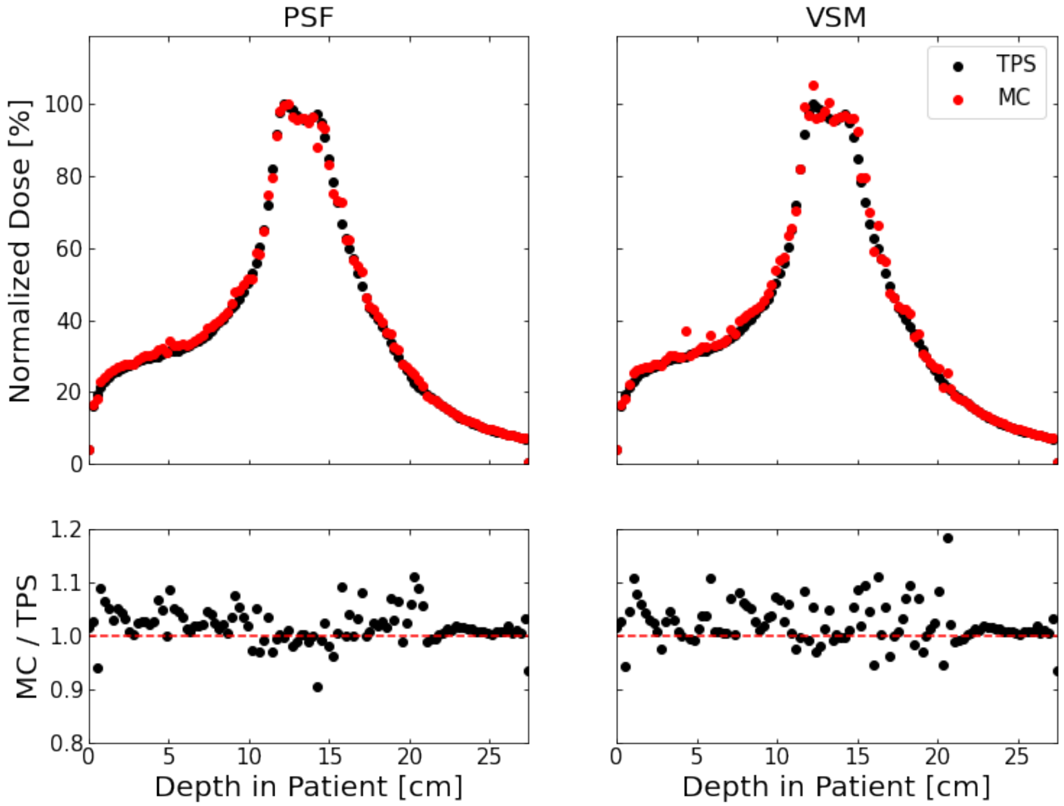

Supplemental Figure 64. Normalized 1D dose profiles for Monte Carlo (MC) doses obtained using a phase-space file (PSF, left) and a virtual source model (VSM, right) for the "Clinical 10XFFF-2" test case. Dose profiles are presented for both the planned dose (black dots) as well as the Monte Carlo doses (red dots).

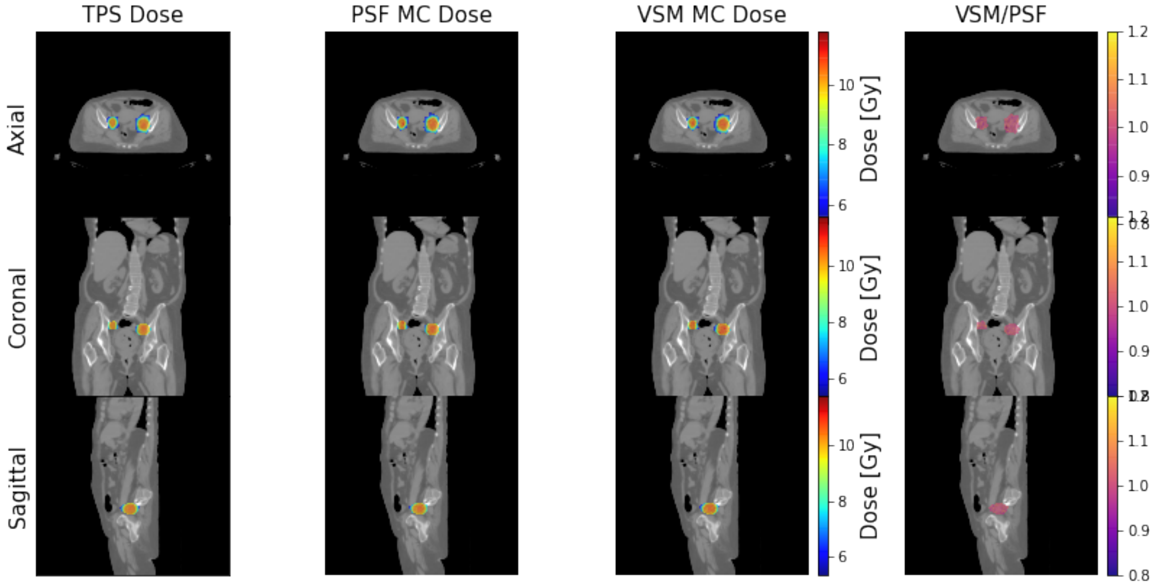

Supplemental Figure 65. Axial, coronal, and sagittal Monte Carlo dose profiles for the "Clinical 10XFFF-3" test case. The first column shows the profiles for the planned (TPS) dose, the second column shows the profiles for the phase-space file (PSF) Monte Carlo dose, the third column shows the profiles for the virtual source model (VSM) Monte Carlo dose, and the fourth column shows the ratio of the VSM to the PSF dose.

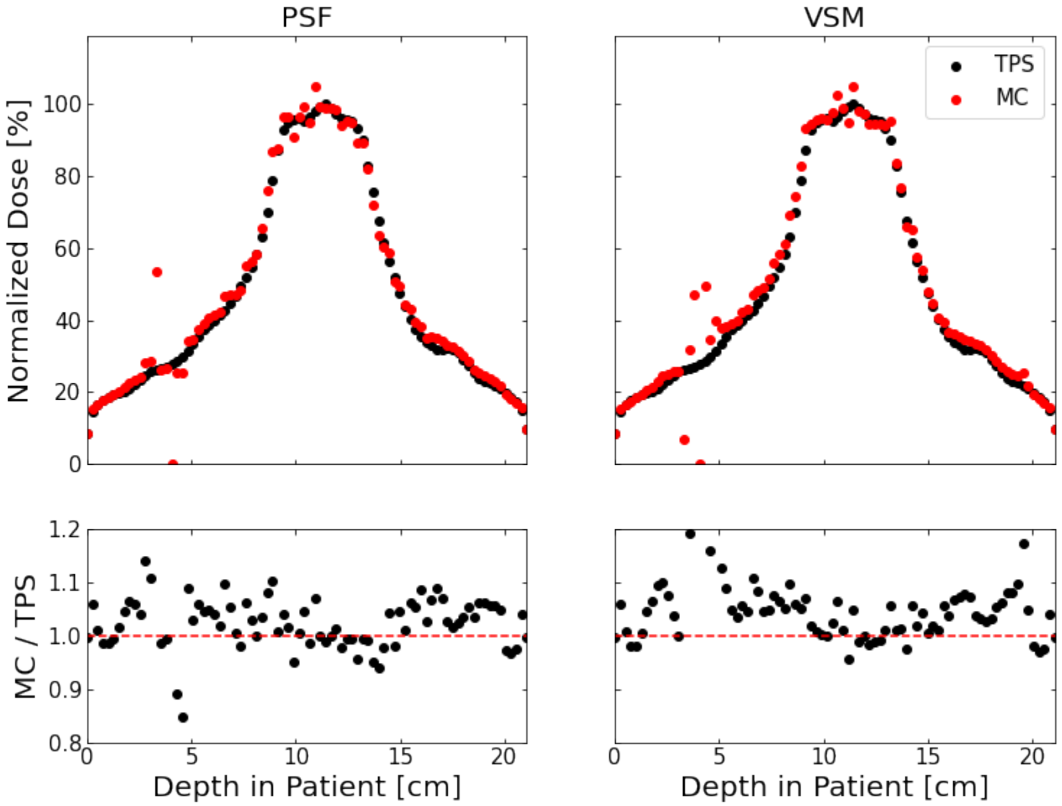

Supplemental Figure 66. Normalized 1D dose profiles for Monte Carlo (MC) doses obtained using a phase-space file (PSF, left) and a virtual source model (VSM, right) for the “Clinical 10XFFF-3” test case. Dose profiles are presented for both the planned dose (black dots) as well as the Monte Carlo doses (red dots).

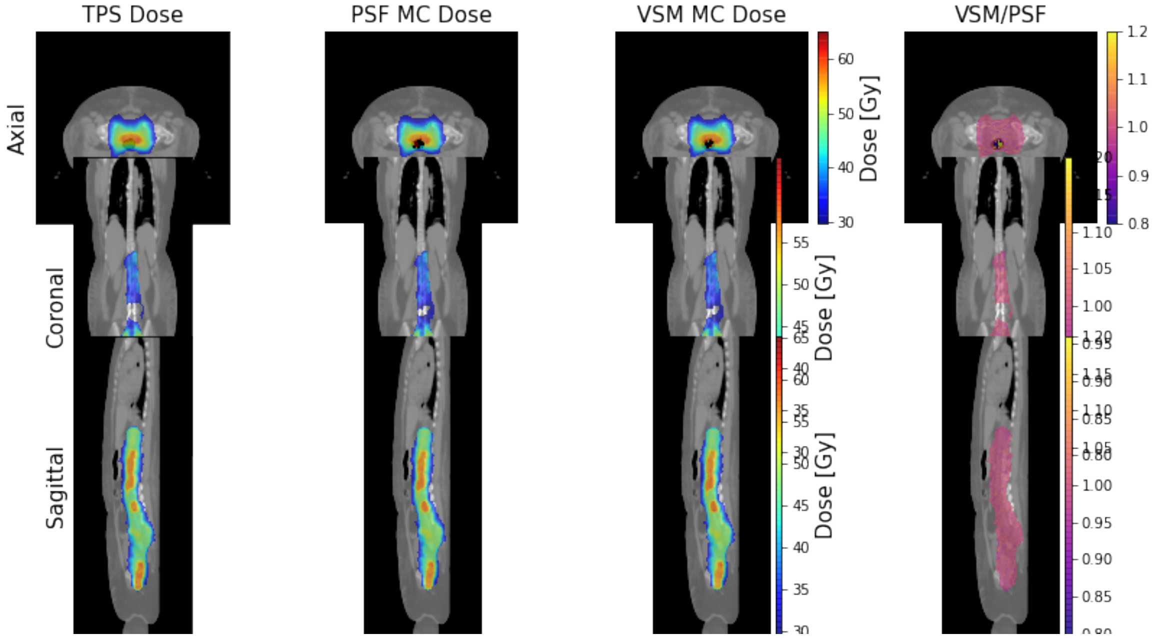

Supplemental Figure 67. Axial, coronal, and sagittal Monte Carlo dose profiles for the "Clinical 10XFFF-4" test case. The first column shows the profiles for the planned (TPS) dose, the second column shows the profiles for the phase-space file (PSF) Monte Carlo dose, the third column shows the profiles for the virtual source model (VSM) Monte Carlo dose, and the fourth column shows the ratio of the VSM to the PSF dose.

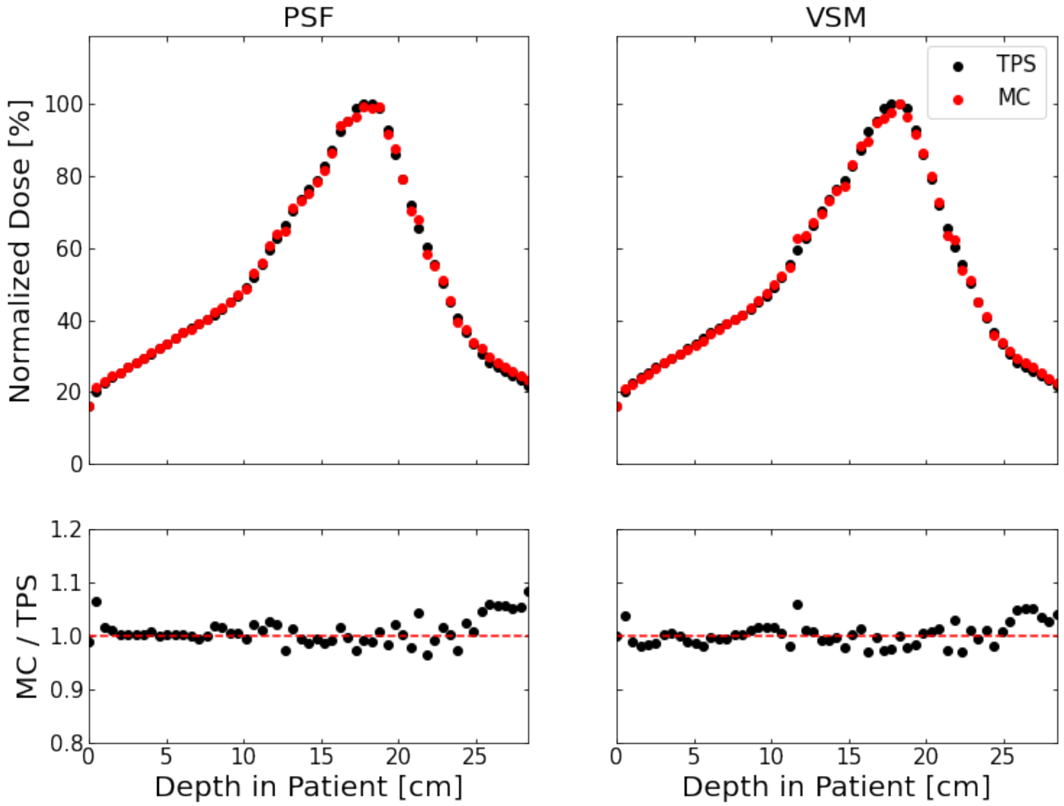

Supplemental Figure 68. Normalized 1D dose profiles for Monte Carlo (MC) doses obtained using a phase-space file (PSF, left) and a virtual source model (VSM, right) for the "Clinical 10XFFF-4" test case. Dose profiles are presented for both the planned dose (black dots) as well as the Monte Carlo doses (red dots).

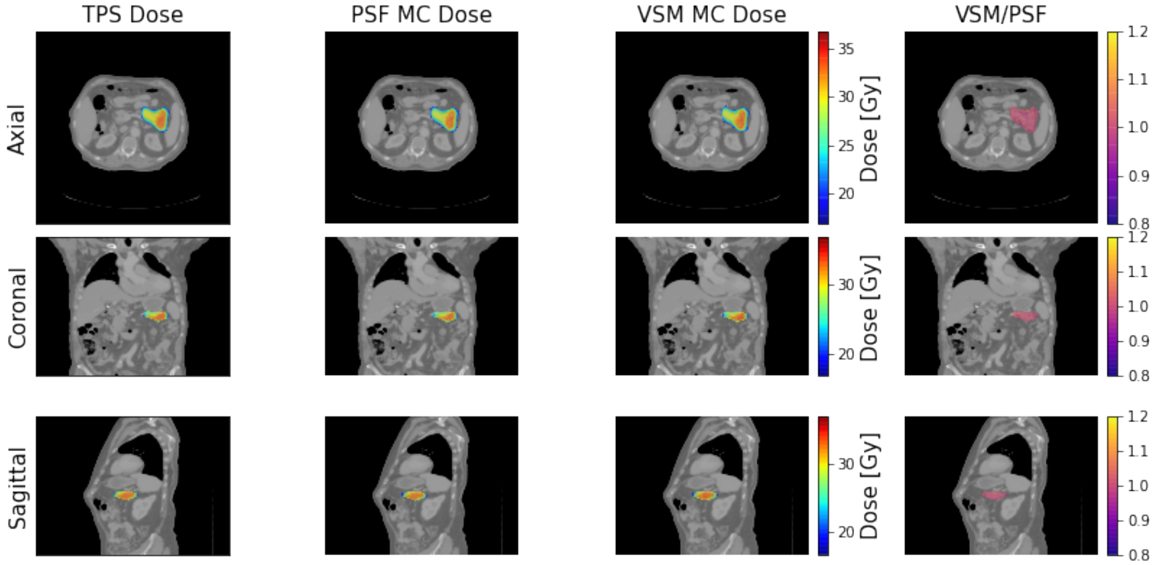

Supplemental Figure 69. Axial, coronal, and sagittal Monte Carlo dose profiles for the "Clinical 10XFFF-5" test case. The first column shows the profiles for the planned (TPS) dose, the second column shows the profiles for the phase-space file (PSF) Monte Carlo dose, the third column shows the profiles for the virtual source model (VSM) Monte Carlo dose, and the fourth column shows the ratio of the VSM to the PSF dose.

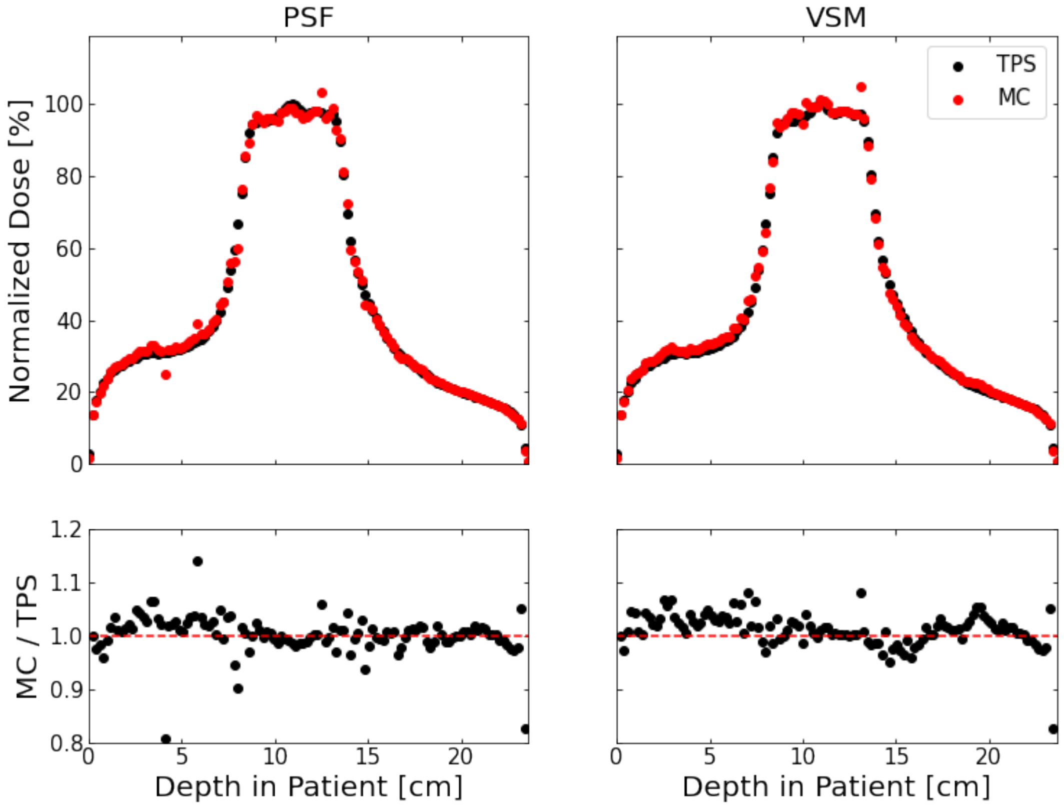

Supplemental Figure 70. Normalized 1D dose profiles for Monte Carlo (MC) doses obtained using a phase-space file (PSF, left) and a virtual source model (VSM, right) for the "Clinical 10XFFF-5" test case. Dose profiles are presented for both the planned dose (black dots) as well as the Monte Carlo doses (red dots).
